# Comparative Study of NOTA-Derived Chelators: Synthesis, Al^18^F/^177^Lu Radiolabeling and Computational Analysis

**DOI:** 10.3390/ijms27167421

**Published:** 2026-08-19

**Authors:** Abir Dhimen, Xavier Assfeld, Gilles Karcher, Charlotte Collet, Samir Acherar

**Affiliations:** 1 Université de Lorraine, CNRS, LRGP, F-54000 Nancy, France; abir.dhimen@univ-lorraine.fr; 2 Nancyclotep, Plateforme d’Imagerie Moléculaire, F-54511 Vandœuvre-lès-Nancy, France; g.karcher@chru-nancy.fr; 3 Université de Lorraine, CNRS, LPCT, F-54000 Nancy, France; xavier.assfeld@univ-lorraine.fr; 4 Université de Lorraine, INSERM, IADI, F-54000 Nancy, France

**Keywords:** bifunctional chelator, hybrid chelator, Al^18^F/^177^Lu theranostic, molecular modeling, radiolabeling, coordination chemistry

## Abstract

The development of hybrid bifunctional chelators (BFCs) capable of efficiently coordinating both diagnostic and therapeutic radionuclides remains a major challenge in theranostic radiopharmaceutical design. Herein, two novel hybrid NOTA-derived chelators, **NO2A-2C-AHM** and **NODAGA-HM**, were developed from the previously reported **NO2A-AHM** BFC to improve the coordination of ^177^Lu while preserving efficient Al^18^F labeling. Both ligands were successfully synthesized and fully characterized by NMR and mass spectrometry analyses. Their coordination properties toward {AlF}^2+^ and Lu^3+^ were thoroughly investigated by molecular modeling, non-radioactive complexation, and radiolabeling studies. Both chelators efficiently formed AlF complexes, exhibiting high complexation yields and successful Al^18^F radiolabeling with good stability. In contrast, only **NODAGA-HM** efficiently coordinated Lu^3+^, whereas **NO2A-2C-AHM** failed to form stable Lu complexes under both non-radioactive and radiolabeling conditions. Optimized radiolabeling of **NODAGA-HM** with ^177^Lu afforded high radiochemical conversion, consistent with the molecular modeling predictions. These results demonstrate that the spatial positioning of the additional donor arm, rather than linker elongation alone, is a key determinant for efficient coordination of larger trivalent radiometals. Thus, **NODAGA-HM** emerged as the most promising candidate for further development of targeted radiopharmaceuticals based on the Al^18^F/^177^Lu theranostic approach.

## 1. Introduction

The development of versatile bifunctional chelators (BFCs) is a major challenge in theranostic radiopharmaceutical design, as these compounds enable the use of the same targeting vector with matched diagnostic and therapeutic radionuclides. This strategy facilitates accurate dosimetry and improves the prediction of tracer biodistribution. Such BFCs must form exceptionally stable radiometal complexes while allowing reliable conjugation to biomolecules, thereby ensuring the selective delivery of diagnostic and therapeutic radionuclides to cancer cells.

This concept lies at the core of the radiotheranostic approach, which generally consists of two complementary phases [1]. The first phase relies on molecular imaging, most commonly positron emission tomography (PET), using a targeting vector radiolabeled with a positron-emitting radionuclide. This step enables visualization of biological target expression and the assessment of radiotracer biodistribution, tumor uptake, and accumulation in healthy organs. Such information is essential for patient selection and for predicting the radiation dose that would be delivered during therapy [2]. If the imaging results are favorable, a second therapeutic phase is initiated. In this phase, the same targeting vector, or a closely related analog, is labeled with a therapeutic radionuclide emitting α- or β^−^-particles. Because the radionuclide is selectively delivered to the tumor cells through the targeting vector, the emitted radiation is deposited closest to the tumor cells, thereby maximizing therapeutic efficacy while minimizing the irradiation of surrounding healthy organs. The combination of diagnostic imaging and targeted radionuclide therapy represents a powerful tool for personalized medicine [3].

**Figure 1:**
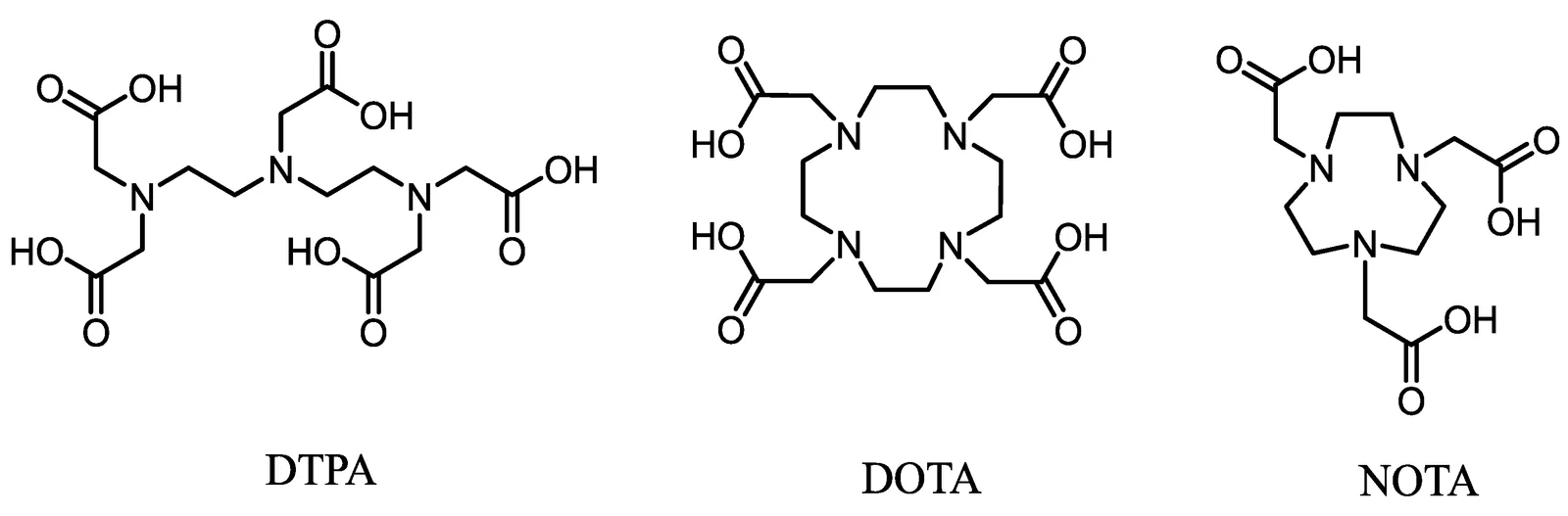
Chemical structures of DTPA, DOTA, and NOTA chelators.

In radiotheranostics, a radiopharmaceutical generally consists of a targeting vector responsible for the selective recognition of a biological target and a radionuclide providing either diagnostic or therapeutic functionality. Depending on the radionuclide, its incorporation may be achieved either through direct covalent attachment or, more commonly for radiometals such as ^68^Ga and ^177^Lu, through coordination chemistry involving an appropriate chelator [4]. Among the numerous chelators described in the literature, polyaminopolycarboxylate ligands are among the most widely used in radiopharmaceutical chemistry. Their main advantage is their ability to form highly stable complexes with a broad range of metal ions, thereby minimizing the risk of metal release under physiological conditions [5]. Two major families of chelators have been reported: acyclic chelators, such as DTPA (diethylenetriaminepentaacetic acid), and macrocyclic chelators, such as NOTA (1,4,7-triazacyclononane-1,4,7-triacetic acid) and DOTA (1,4,7,10-tetraazacyclododecane-1,4,7,10-tetraacetic acid) (Figure 1).

In general, metal complexes formed with macrocyclic chelators exhibit greater thermodynamic stability and kinetic inertness than those formed with acyclic chelators. Consequently, acyclic chelator–metal complexes are generally more susceptible to transmetalation and dissociation in vivo because of competition from endogenous metal ions present in the physiological environment [6]. DOTA is the most widely used macrocyclic chelator in radiopharmaceutical development. It forms highly stable complexes with various trivalent radiometals, including ^177^Lu and ^111^In [6]. A notable example is [^177^Lu]Lu-DOTATATE, which is now routinely used in clinical practice for the treatment of neuroendocrine tumors [7]. In contrast, NOTA has a smaller coordination cavity, making it particularly well suited to smaller radiometals such as ^68^Ga and ^64^Cu. NOTA is commonly regarded as a reference chelator for ^68^Ga-radiolabeling owing to its rapid complexation kinetics and excellent in vivo stability [8].

Among radionuclides used for PET imaging, ^18^F occupies a privileged position because of its favorable physical properties. As a positron emitter with a half-life of 109.8 min, it provides high-resolution PET images while allowing centralized production and distribution to imaging centers. A major advance in ^18^F-radiochemistry was the introduction of the Al^18^F-labeling strategy by McBride and co-workers in 2009 [9]. In this approach, ^18^F first forms a stable complex with Al^3+^, generating the {Al^18^F}^2+^ species, which can subsequently be coordinated by suitable chelators such as NOTA to afford Al^18^F–chelator complexes. This method combines the favorable imaging properties of ^18^F with the operational simplicity of radiometal-based labeling procedures, including rapid radiolabeling and straightforward purification. Furthermore, the relatively rapid in vivo pharmacokinetics of peptide bioconjugates are well matched to the half-life of ^18^F, making this radionuclide particularly suitable for the development of radiopharmaceuticals intended for theranostic applications [10,11].

Among therapeutic radionuclides, ^177^Lu has emerged as one of the most widely used in targeted radionuclide therapy. With a half-life of 6.7 days, it emits β^−^-particles with energies suitable for the irradiation of small to medium-sized tumors. In addition, its decay is accompanied by low abundance γ-photons (113 and 208 keV), enabling post-therapy imaging and dosimetric assessment [12]. These combined therapeutic and imaging properties make ^177^Lu particularly attractive for theranostic applications. Several macrocyclic chelators, including DOTA [13], NOTA [14], and DOTAGA (2-[1,4,7,10-tetraazacyclododecane-4,7,10-tris(*t*-butyl acetate)]-pentanedioic acid-1*t*-butyl ester) [15], have demonstrated an excellent ability to form stable complexes with ^177^Lu, enabling the development of radiopharmaceuticals with high in vivo stability [16].

**Figure 2:**
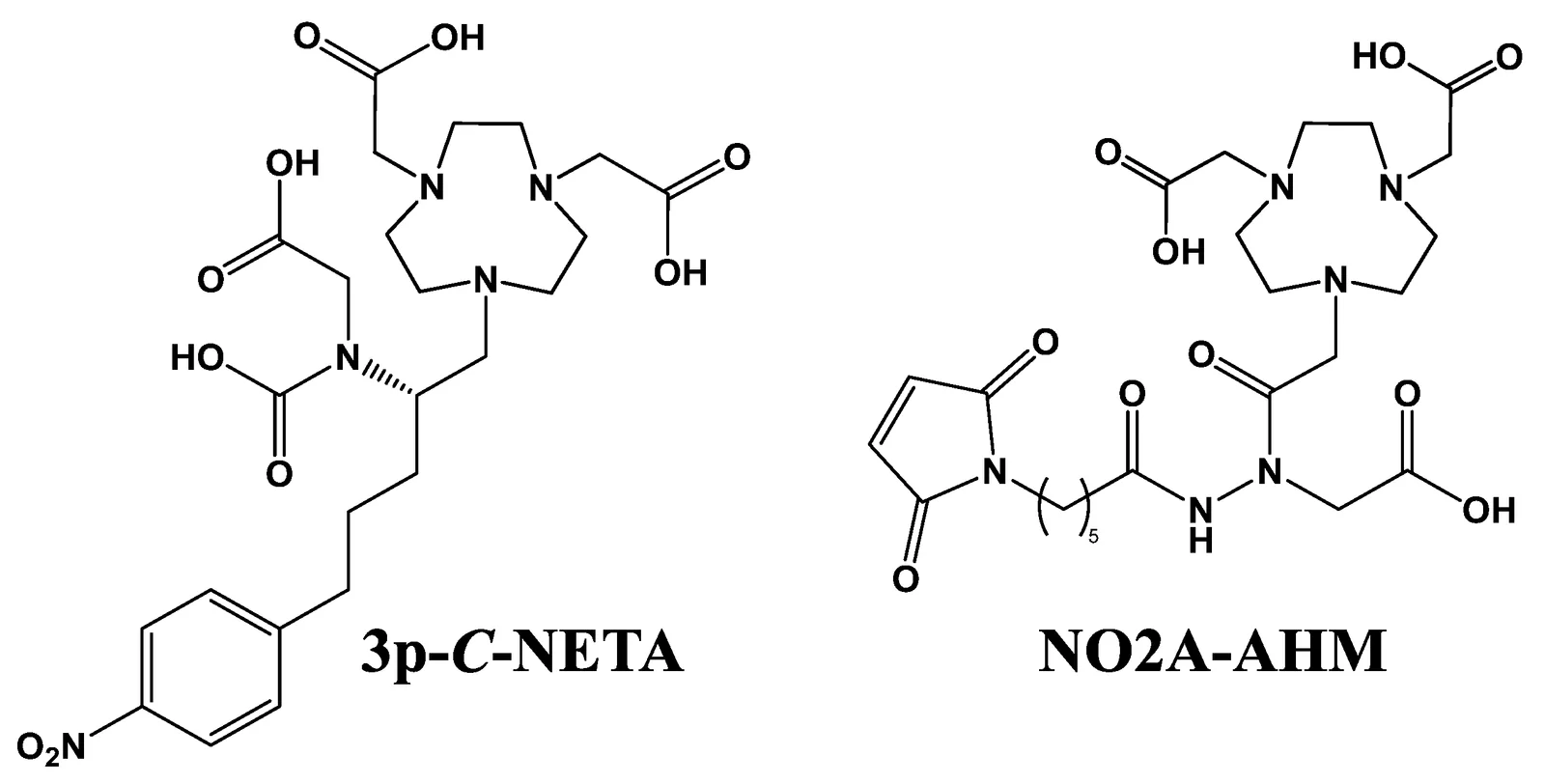
Chemical structures of **3p-*****C*****-NETA** (Chong et al. 2011 [19]) and **NO2A-AHM** (Wagner et al. 2023 [22]).

The combination of Al^18^F for diagnostic imaging and ^177^Lu for radionuclide therapy represents an exceptionally attractive theranostic pair. Such a strategy would allow the same targeting vector to be used for both patient stratification and treatment while preserving comparable pharmacokinetic properties. However, the development of chelators capable of efficiently accommodating both the {Al^18^F}^2+^ moiety and Lu^3+^ remains challenging because of their markedly different coordination requirements. The development of a chelating agent based on the Al^18^F/^177^Lu theranostic pair has been only sparsely explored. Chong and co-workers [17] were among the first to report a BFC, **3p-*****C*****-NETA** (Figure 2), designed for theranostic applications based on NETA-derived structures. This molecule combines a macrocyclic NOTA scaffold with two acyclic donor arms. The incorporation of these additional chelating arms enhances complex stability by better accommodating radionuclides with larger ionic radius, such as ^177^Lu, and by increasing the number of coordination interactions between the metal and the chelator. This chelator demonstrated the ability to complex a broad range of radiometals, including Al^18^F and ^177^Lu, while maintaining good stability, as summarized in Table 1. These results highlighted the versatility of hybrid cyclic–acyclic chelators for accommodating radiometals with diverse coordination requirements. The versatility of **3p-*****C*****-NETA** has been demonstrated through its conjugation to PSMA-targeting and somatostatin receptor-targeting ligands, confirming its potential as a theranostic chelator for both diagnostic and therapeutic radiometals with excellent radiochemical conversion rates of around 80% and quantitative for Al^18^F and ^177^Lu respectively [18].

Building upon this concept, our group recently developed a novel cyclic–acyclic hybrid chelator devoid of any asymmetric carbon center, namely **NO2A-AHM**, for theranostic applications combining Al^18^F chemistry for PET imaging with the complexation of therapeutic radiometals such as ^177^Lu. This chelator consists of a NOTA macrocycle coupled to a hydrazine-functionalized unit through a chelating arm and a maleimide linker, enabling conjugation to thiol-containing biomolecules such as peptides and antibodies.

The architecture of **NO2A-AHM** was designed to increase structural flexibility and allow the formation of five to seven coordination bonds, depending on the metal ion involved. Such flexibility was expected to reconcile the distinct geometric requirements of both the {AlF}^2+^ complex and larger trivalent ions such as Lu^3+^ (Figure 2). From a methodological standpoint, **NO2A-AHM** was conceived as a “mismatched” chelator intended to overcome the conventional limitation of using NOTA derivatives for Al^18^F and DOTA derivatives for ^177^Lu by providing a single chelating platform suitable for both radionuclides. Proof of concept was established through radiolabeling of **NO2A-AHM** with {Al^18^F}^2+^ (radiochemical conversion (RCC) of 76%) and preliminary radiolabeling with ^177^Lu (RCC of 24%), demonstrating the experimental feasibility of this dual-radionuclide strategy [21]. However, the relatively low RCC obtained with ^177^Lu indicated that further optimization of the chelator structure was required. Furthermore, the versatility of the **NO2A-AHM** platform was demonstrated through its successful conjugation to targeting agents directed against PSMA and integrins (RGD), highlighting its potential for the development of targeted theranostic radiopharmaceuticals [22].

Recent advances in molecular design have demonstrated that targeted structural modifications can profoundly influence the physicochemical and functional properties of molecular platforms. Structure-activity relationship studies have shown that variations in substituent identity, incorporation of nitrogen-containing moieties, and alterations in side-chain architecture can markedly affect molecular interactions and biological performance [23,24]. Similarly, precise structural tailoring has been employed to regulate stability, self-assembly, and activation processes in prodrug systems [25]. Although these investigations were conducted outside the field of radiometal chelation, they illustrate a fundamental principle that is highly relevant to bifunctional chelator (BFC) development: even subtle changes in molecular architecture can have a substantial impact on system behavior. In the context of chelator design, this concept underscores the importance of optimizing donor-atom number and arrangement, linker length and flexibility, as well as coordination-cavity geometry to meet the distinct coordination requirements of different radiometals.

**Figure 3:**
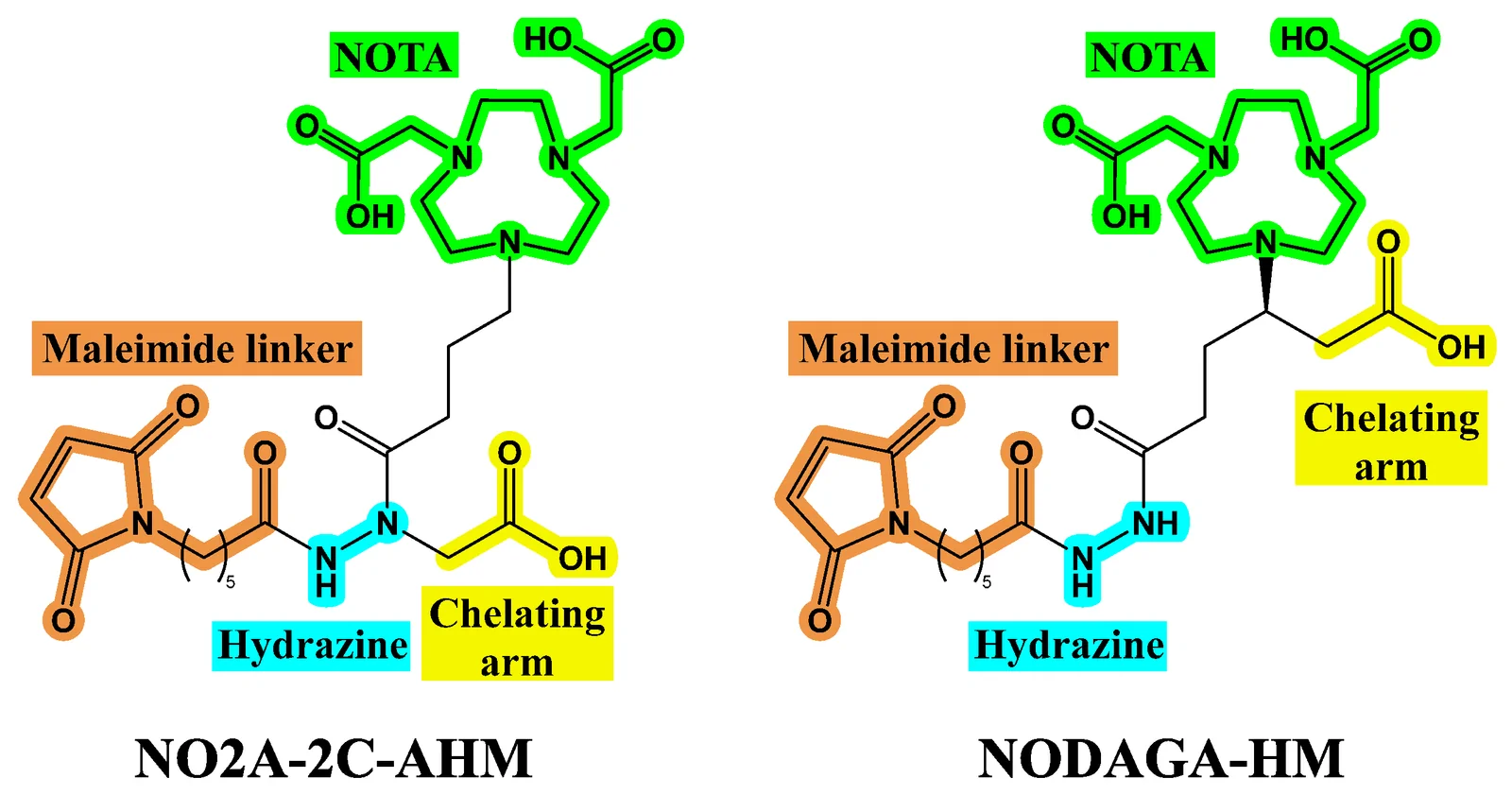
Chemical structures of **NO2A-2C-AHM** and **NODAGA-HM**.

To address these limitations, we designed two new BFCs, **NO2A-2C-AHM** and **NODAGA-HM** (Figure 3), derived from structural modifications of **NO2A-AHM**, with the objective of assessing how linker extension and donor-arm positioning influence ^177^Lu coordination. To elucidate the influence of chelator architecture on metal complexation and radiolabeling efficiency, two complementary structural strategies were investigated. The first strategy consisted of extending the linker connecting the NOTA macrocycle to the hydrazine moiety by two additional carbon atoms while retaining the chelating arm on the hydrazine unit (**NO2A-2C-AHM**). This modification increases the distance between the hydrazide function and the NOTA macrocycle, providing greater spatial separation between these two structural elements. The second strategy also involved introducing two additional carbon atoms into the linker connecting the NOTA macrocycle and the hydrazine moiety. In contrast to **NO2A-2C-AHM**, the chelating arm was positioned closer to the NOTA macrocycle (**NODAGA-HM**), thereby altering the spatial arrangement of the donor groups around the metal-binding site.

**Figure 4:**
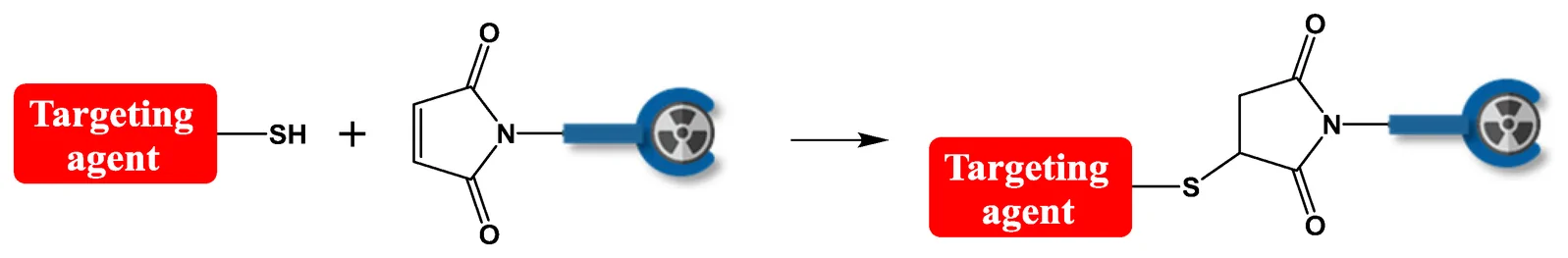
Proposed structure of our theranostic radiopharmaceuticals obtained through thiol–maleimide click chemistry reactions.

These two compounds provide an opportunity to evaluate the influence of chelating-arm positioning on complexation behavior and radiolabeling performance. Owing to the presence of the additional chelating arm, both BFCs are expected to form stable complexes with a variety of radiometals, ranging from small ions such as aluminum to larger radiometals such as lutetium, for which the additional coordination site may contribute to enhanced complex stability. The most promising candidate will subsequently be selected for conjugation to a biological targeting vector. For this purpose, a thiol–maleimide, click chemistry reaction was selected because of its rapid kinetics, high efficiency, and excellent selectivity toward thiol groups. The presence of a maleimide moiety within the BFC structure enables conjugation to a wide range of thiol-functionalized targeting vectors, including peptides, antibodies, and related biomolecules (Figure 4).

In the present study, the synthesis and physicochemical characterization of two novel NOTA-derived BFCs, **NO2A-2C-AHM** and **NODAGA-HM**, are reported. Their coordination properties toward {AlF}^2+^ and Lu^3+^ were investigated through computational analysis and experimental complexation studies, with particular attention devoted to the influence of chelating arm positioning on coordination. Finally, the suitability of these BFCs for radiotheranostic applications was assessed through Al^18^F and ^177^Lu radiolabeling experiments.

## 2. Results and Discussion

### 2.1. Synthesis of BFCs

#### 2.1.1. Synthesis of **NO2A-2C-AHM**

**Scheme 1:**
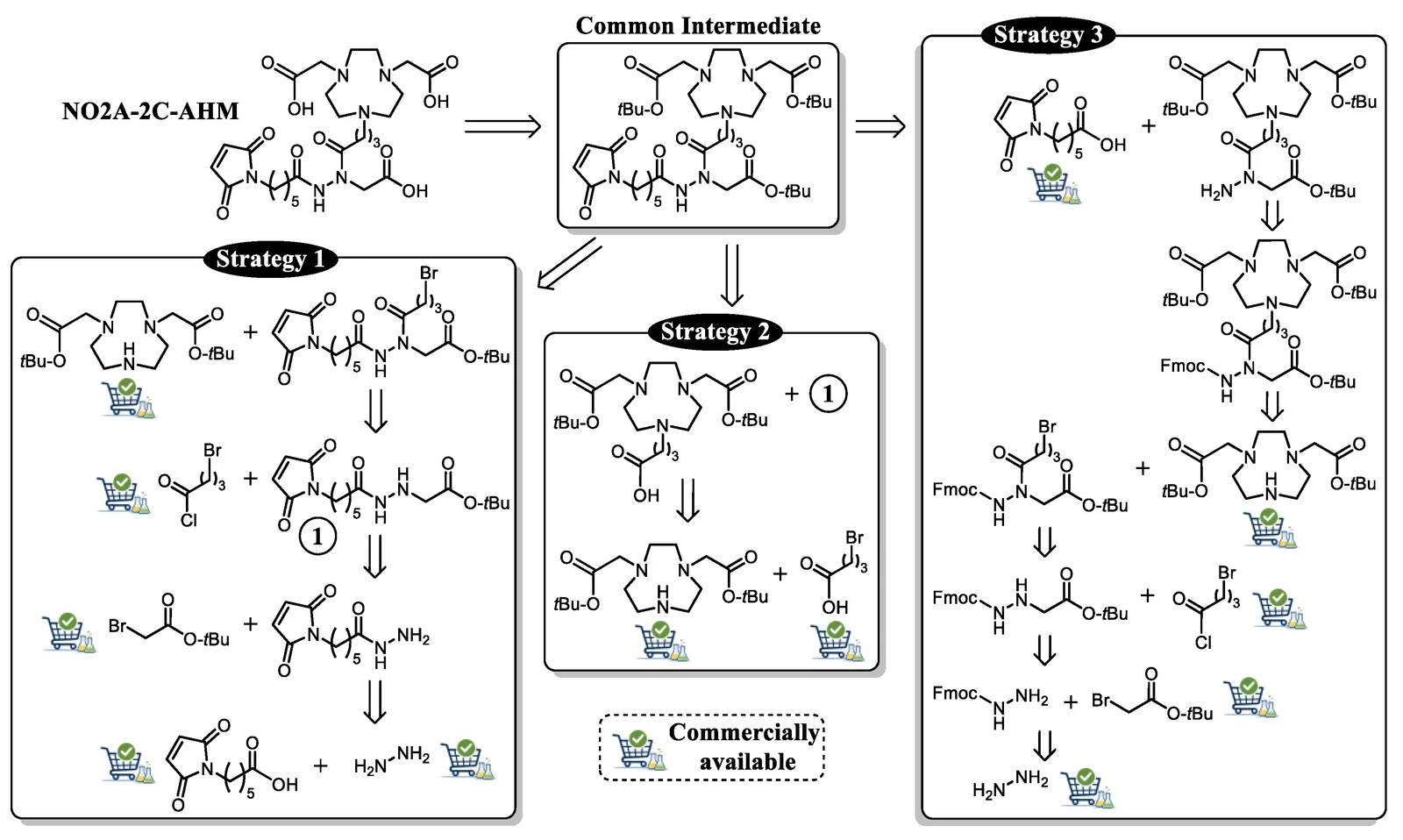
Retrosynthetic analysis of the three synthetic routes investigated toward **NO2A-2C-AHM**.

**Scheme 2:**
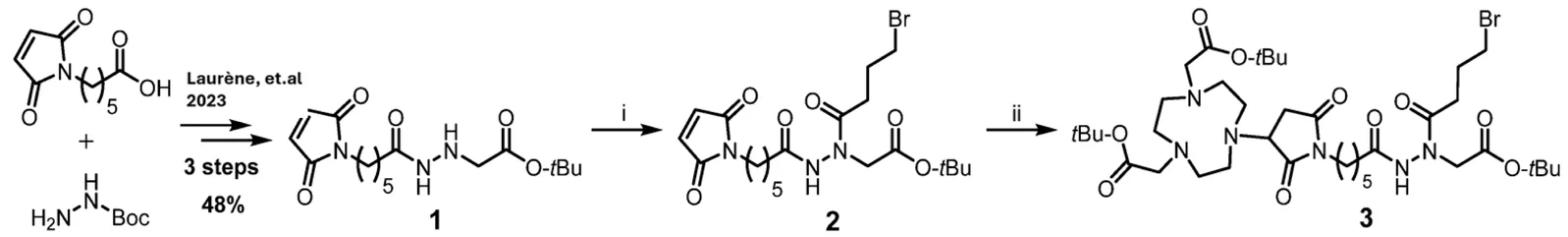
First strategy for the synthesis of **NO2A-2C-AHM**. Reagents and conditions: (i) 4-bromobutyryl chloride, 2,6-lutidine, DCM, r.t., 30 min, 70%; (ii) NO2A-*t*Bu, DIPEA, CH_3_CN, r.t., overnight, 50%. (Laurène et al. 2023 [21]).

The synthesis of **NO2A-2C-AHM** proved more challenging than previously reported for **NO2A-AHM** [21], requiring the evaluation of three successive synthetic strategies (Scheme 1). Compound **1**, a key intermediate previously reported by our group for the synthesis of **NO2A-AHM**, was prepared from *tert*-butyl carbazate and 6-maleimidohexanoic acid according to the published procedure in three steps and with an overall yield of 48% (Scheme 2). At this stage, the synthetic route toward **NO2A-2C-AHM** diverges from that of **NO2A-AHM** through the introduction of an extended linker between the NOTA macrocycle and the hydrazine moiety.

The first synthetic strategy was designed by adapting the route previously developed for **NO2A-AHM** (Scheme 1 and Scheme 2). Starting from compound **1**, the linker connecting the NOTA macrocycle to the hydrazine moiety was extended by two carbon atoms through acylation with 4-bromobutyryl chloride in the presence of 2,6-lutidine, affording the corresponding intermediate **2** in 70% yield (Scheme 2). Introduction of the NO2A-*t*Bu chelator was then attempted under the S_N_2 conditions previously optimized for **NO2A-AHM** using NO2A-*t*Bu (0.9 equiv.) and DIPEA in acetonitrile [21]. However, the desired substitution product was not obtained. Instead, compound **3** was isolated as the major product and was attributed to a Michael addition of NO2A-*t*Bu to the activated maleimide double bond. This outcome indicates that, under the conditions employed, nucleophilic attack on the highly electrophilic maleimide moiety was kinetically favored over the desired S_N_2 displacement of the bromide. Consequently, the synthetic sequence successfully applied to **NO2A-AHM** could not be directly transposed to the synthesis of **NO2A-2C-AHM** (Scheme 2).

**Scheme 3:**
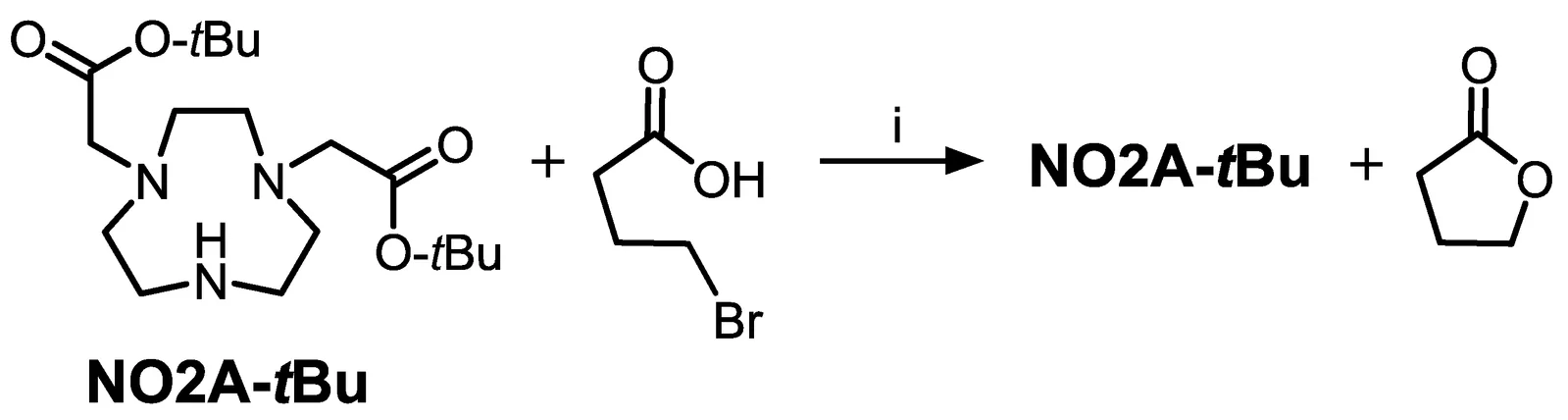
Second strategy for the synthesis of **NO2A-2C-AHM**. Reagents and conditions: (i) 4-bromobutyric acid, base (DIPEA, Et_3_N, or K_2_CO_3_), DCM, r.t., 2–7 days.

To circumvent the undesired Michael addition observed in the first strategy, a second approach was investigated (Scheme 1). The rationale was to introduce the extended linker prior to the installation of the maleimide moiety, thereby avoiding the presence of a highly electrophilic double bond during the nucleophilic substitution step. This approach involved the functionalization of NO2A-*t*Bu with 4-bromobutyric acid [26] before coupling with compound **1**. However, no conversion was observed despite screening several bases, including DIPEA, Et_3_N, and K_2_CO_3_ (Scheme 3). Analysis of the reaction mixtures by ^1^H NMR spectroscopy revealed the spontaneous cyclization of 4-bromobutyric acid to the corresponding lactone, dihydrofuran-2(*3H*)-one (Scheme 3). This intramolecular cyclization consumed the alkylating agent and prevented the formation of the desired intermediate. Consequently, the second synthetic strategy was also unsuccessful.

**Scheme 4:**
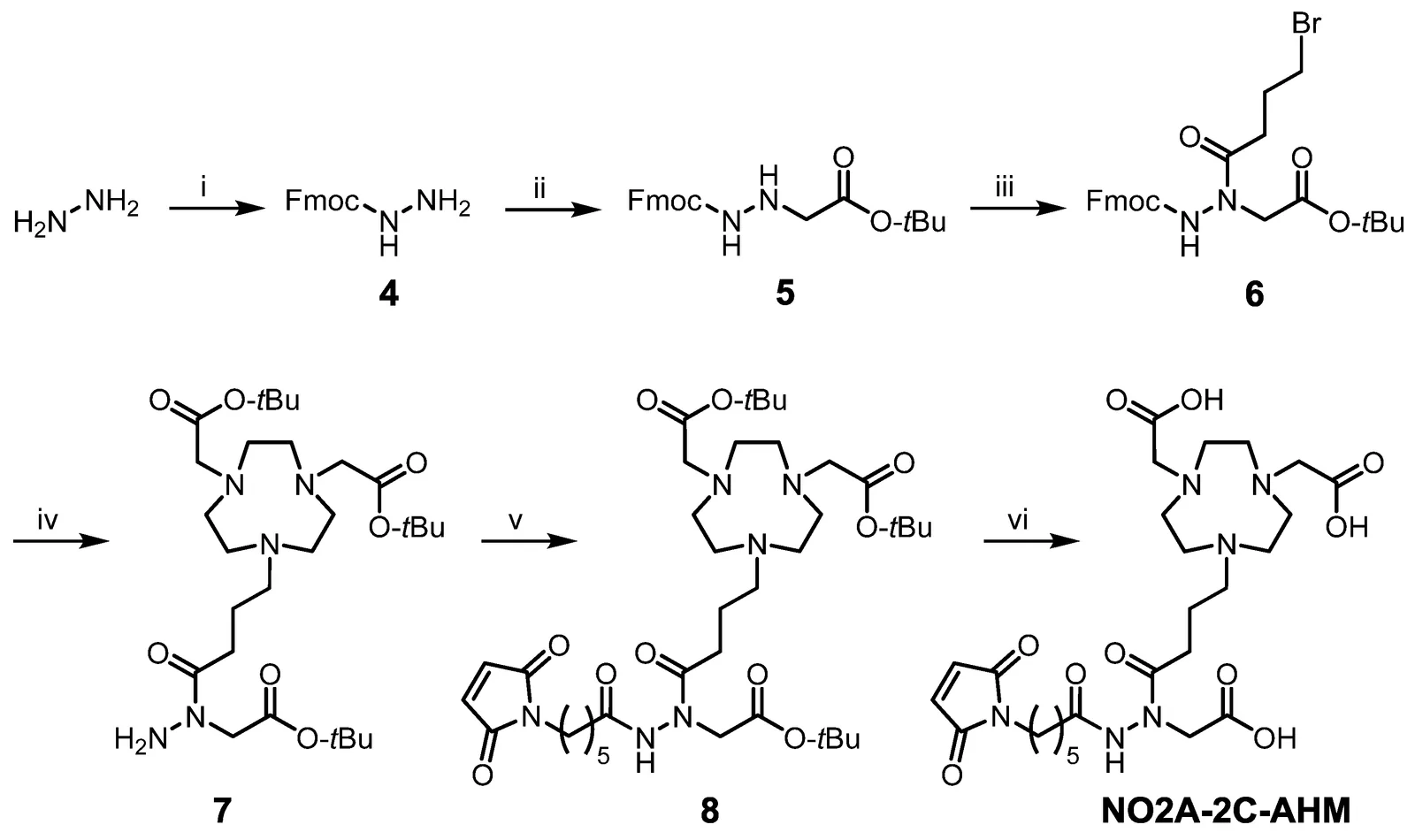
Third strategy for the synthesis of **NO2A-2C-AHM**. Reagents and conditions: (i) hydrazine hydrate, Fmoc-Cl, CH_3_CN/H_2_O (1/1, *v*/*v*), 0 °C, 2.5 h, then 0 °C to r.t., overnight, 91%; (ii) *tert*-butyl bromoacetate, K_2_CO_3_, DMF, 0 °C to r.t., 20 h, 60%; (iii) 4-bromobutyryl chloride, 2,6-lutidine, DCM, r.t., 30 min, 74%; (iv) NO2A-*t*Bu, DIPEA, CH_3_CN, 0 °C to r.t., 20 h, 55%; (v) 6-maleimidohexanoic acid, HATU, NMM, DCM/DMF, 0 °C to r.t., 15 h, 80%; (vi) TFA, DCM, r.t., 9 h, quantitative.

Following the failure of the first two approaches, a third synthetic strategy was designed (Scheme 1 and Scheme 4). In this route, the chelating arm was introduced prior to maleimide installation while avoiding intermediates prone to Michael addition or intramolecular cyclization. This strategy ultimately provided access to **NO2A-2C-AHM** in six steps, with an 18% overall yield from *tert*-butyl carbazate and 6-maleimidohexanoic acid (Scheme 4). The synthesis began with Fmoc protection of hydrazine to prevent undesired disubstitution during the subsequent alkylation step. Fmoc-protected hydrazine **4** was obtained in 91% yield according to a reported procedure [27]. The chelating arm was then introduced through an S_N_2 reaction with *tert*-butyl bromoacetate following the procedure reported by Spiegel et al. [28]. Compound **5** was isolated in 60% yield after purification, slightly lower than the previously reported 65% yield. Besides the desired product, several by-products were formed, including a dialkylated derivative resulting from overalkylation of the hydrazine moiety. Intermediate **5** was subsequently acylated with 4-bromobutyryl chloride to afford compound **6**. In contrast to the second strategy, in which spontaneous lactonization of 4-bromobutyric acid prevented further functionalization, introduction of the linker as the corresponding acid chloride successfully circumvented this limitation. Since acylation generates HBr, a base was required to neutralize the reaction medium. Pyridine is commonly used for this purpose; however, its nucleophilic character can promote undesired substitution of the bromide functionality, as previously reported by Rahim et al. [29]. To minimize such side reactions, 2,6-lutidine was selected as a sterically hindered and less nucleophilic base, affording compound **6** in 74% yield after purification (Scheme 4). The NOTA chelator was then introduced through an S_N_2 reaction between compound **6** and NO2A-*t*Bu under basic conditions, affording compound **7** in 55% yield after RP-C18 HPLC purification. Interestingly, the reaction conditions also promoted simultaneous removal of the Fmoc protecting group, eliminating the need for an additional deprotection step and simplifying the synthetic sequence (Scheme 4). The maleimide moiety was subsequently introduced through peptide-coupling conditions using 6-maleimidohexanoic acid, yielding compound **8** in 80% yield. Importantly, installation of the maleimide functionality at this late stage avoided the undesired Michael addition encountered in the first synthetic strategy. Finally, global deprotection of the *tert*-butyl esters furnished **NO2A-2C-AHM** as a white solid in quantitative yield and with a chemical purity greater than 98%, as determined by RP-HPLC at 214 nm. Overall, this third strategy successfully addressed the limitations encountered in the previous approaches and provided robust and reliable access to the target BFC, **NO2A-2C-AHM**. Although the overall yield (18%) is moderate, it primarily reflects the cumulative losses incurred throughout the six-step synthetic sequence and the multiple purification procedures. Nevertheless, the synthetic route proved robust and reproducible, affording **NO2A-2C-AHM** in sufficient quantity and purity for comprehensive physicochemical characterization and radiochemical evaluation.

#### 2.1.2. Synthesis of **NODAGA-HM**

**Scheme 5:**
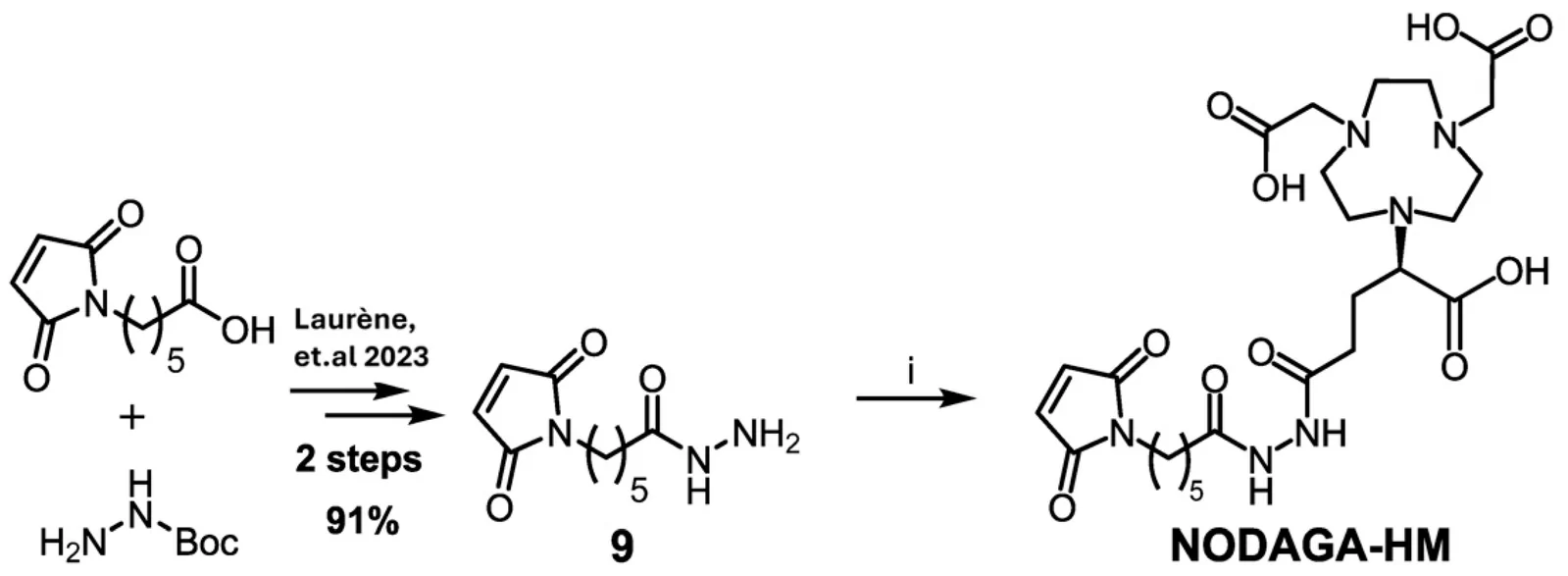
Strategy for the synthesis of **NODAGA-HM**. Reagents and conditions: (i) NODAGA-NHS, DIPEA, DMF, 0 °C to r.t., 15 h, 50%. (Laurène et al. 2023 [21]).

The design of **NO2A-2C-AHM** involved the introduction of a two-carbon extension between the NOTA macrocycle and the hydrazine unit. Although this modification increased the distance between these two structural elements, it also displaced the additional chelating arm (acetate donor arm) away from the coordination cavity. To evaluate whether the position of this donor arm influences metal complexation, **NODAGA-HM** was designed as a complementary BFC analog. In this ligand, the hydrazine-to-macrocycle spacing is preserved while the additional acetate arm is located closer to the coordination site. For the preparation of **NODAGA-HM**, compound **9** was used as a common intermediate from our previously reported **NO2A-AHM** synthetic route [21]. This intermediate was synthesized from *tert*-butyl carbazate and 6-maleimidohexanoic acid in two steps with an overall yield of 91% according to the reported procedure (Scheme 5). **NODAGA-HM** was then synthesized by coupling compound **9** with commercially available NODAGA-NHS ester (Scheme 5). The key step involved a nucleophilic addition–elimination reaction between the primary amine of compound **9** and the activated *N*-hydroxysuccinimide (NHS) ester under basic conditions. To minimize hydrolysis of the activated ester and limit side reactions, the NODAGA-NHS solution was added at 0 °C before the reaction mixture was allowed to warm gradually to room temperature. Under these conditions, selective displacement of the NHS leaving group afforded the desired amide linkage between the NODAGA chelator and the HM spacer. After purification, **NODAGA-HM** was isolated in 50% yield for this coupling step. Overall, **NODAGA-HM** was obtained in three synthetic steps from *tert*-butyl carbazate and 6-maleimidohexanoic acid, with an overall yield of 46% (Scheme 5).

### 2.2. Complexation Studies

To evaluate the coordination properties of the newly synthesized BFCs, non-radioactive complexation studies were performed using aluminum fluoride and lutetium as surrogates of the corresponding radiometals used in PET imaging and radionuclide therapy, respectively. The resulting complexes were characterized by LC-MS and ^1^H NMR spectroscopy, and their stability was further assessed under physiologically relevant conditions.

#### 2.2.1. Aluminum-Fluoride Complexation

**Figure 5:**
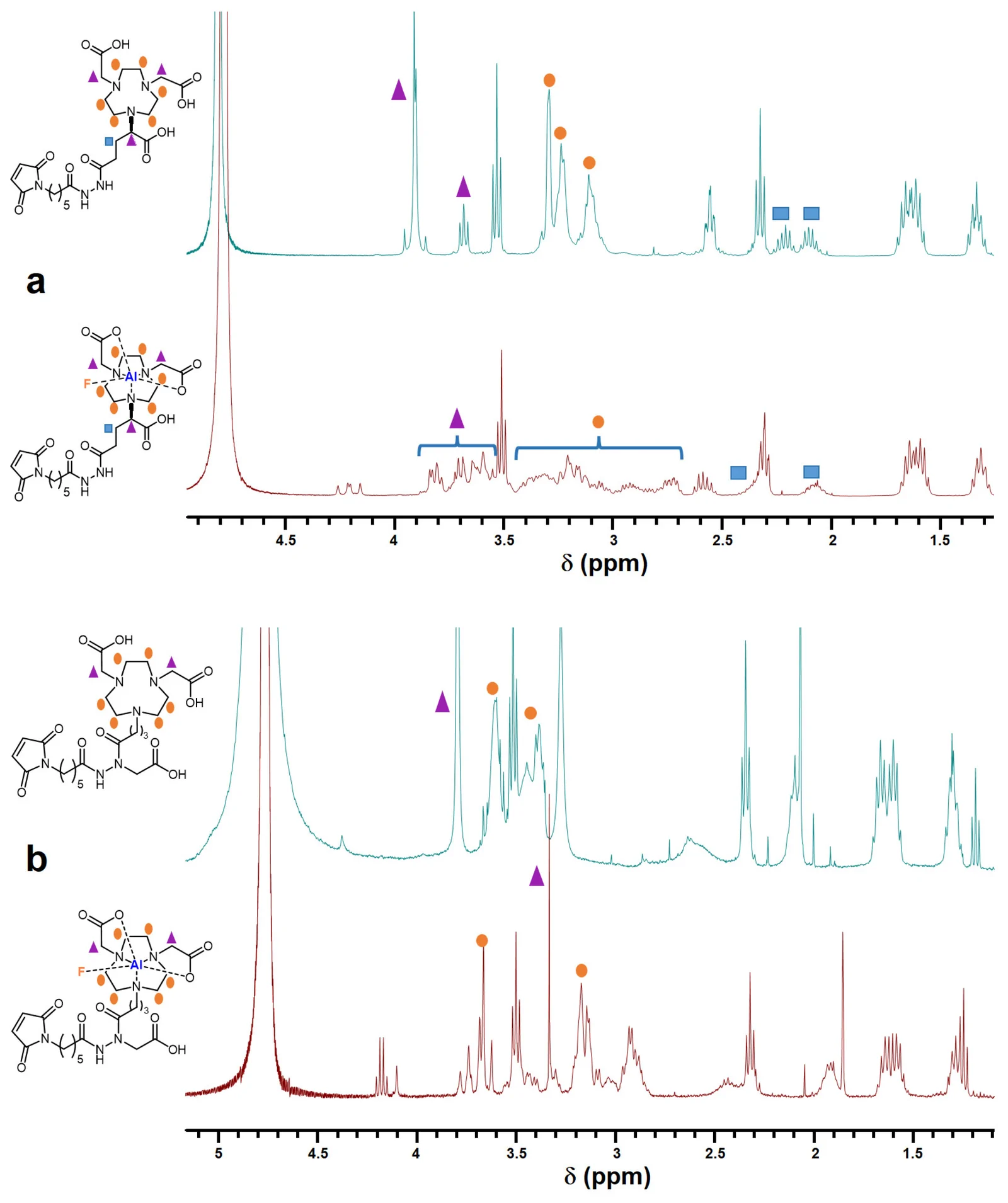
^1^H NMR spectra of (**a**) **AlF-NODAGA-HM** and **NODAGA-HM**, and (**b**) **AlF-NO2A-2C-AHM** and **NO2A-2C-AHM** in D_2_O at 330 K (400 MHz).

Aluminum-fluoride complexation was performed under the optimized conditions previously established for **NO2A-AHM** [21]. Briefly, the {AlF}^2+^ species was generated in situ from AlCl_3_ and NaF in acetate buffer (pH 4.5), followed by reaction with the corresponding BFC at 90 °C in the presence of 50% EtOH. Previous optimization studies showed that elevated temperatures (≥70 °C) are required to achieve near-quantitative complexation (>99%), while the addition of EtOH significantly enhances conversion by improving precursor solubility and facilitating formation of the AlF intermediate [21,30]. These optimized conditions were therefore applied to **NO2A-2C-AHM** and **NODAGA-HM**. The formation of **AlF-NO2A-2C-AHM** and **AlF-NODAGA-HM** was confirmed by LC-MS analysis, with [M + H]^+^ ions detected at 641.25 and 627.22, respectively. The corresponding incorporation rates reached 95%, while isolated yields after RP-HPLC purification were 90% and 80%, respectively. These values compare favorably with those previously reported for **NO2A-AHM** [21], indicating that extension of the linker or repositioning of the additional acetate donor arm does not impair complexation of the {AlF}^2+^ core. The coordination behavior of the corresponding AlF complexes was further investigated by molecular modeling, and the computational results are discussed in Section 2.3.1. Complex formation was further confirmed by ^1^H NMR spectroscopy in D_2_O (Figure 5). For **NODAGA-HM** (Figure 5a), coordination of the {AlF}^2+^ unit induced significant changes in the spectral region associated with the chelating framework. The broad multiplet observed between 3.09 and 3.28 ppm reflects a loss of chemical equivalence of the 12 macrocyclic protons of the NOTA cage upon coordination. In addition, the singlet at 3.88 ppm (assigned to the methylene groups of the acetate arms) and the triplet at 3.67 ppm (corresponding to the proton in the γ-position relative to the hydrazide carbonyl) were also affected by complex formation. Similar changes in chemical shifts and multiplicities were observed for **NO2A-2C-AHM** (Figure 5b), particularly for signals associated with the NOTA cage and acetate arm. These spectral modifications are consistent with coordination-induced changes in the electronic environment of the chelator and confirm the formation of the corresponding AlF complexes.

**Figure 6:**
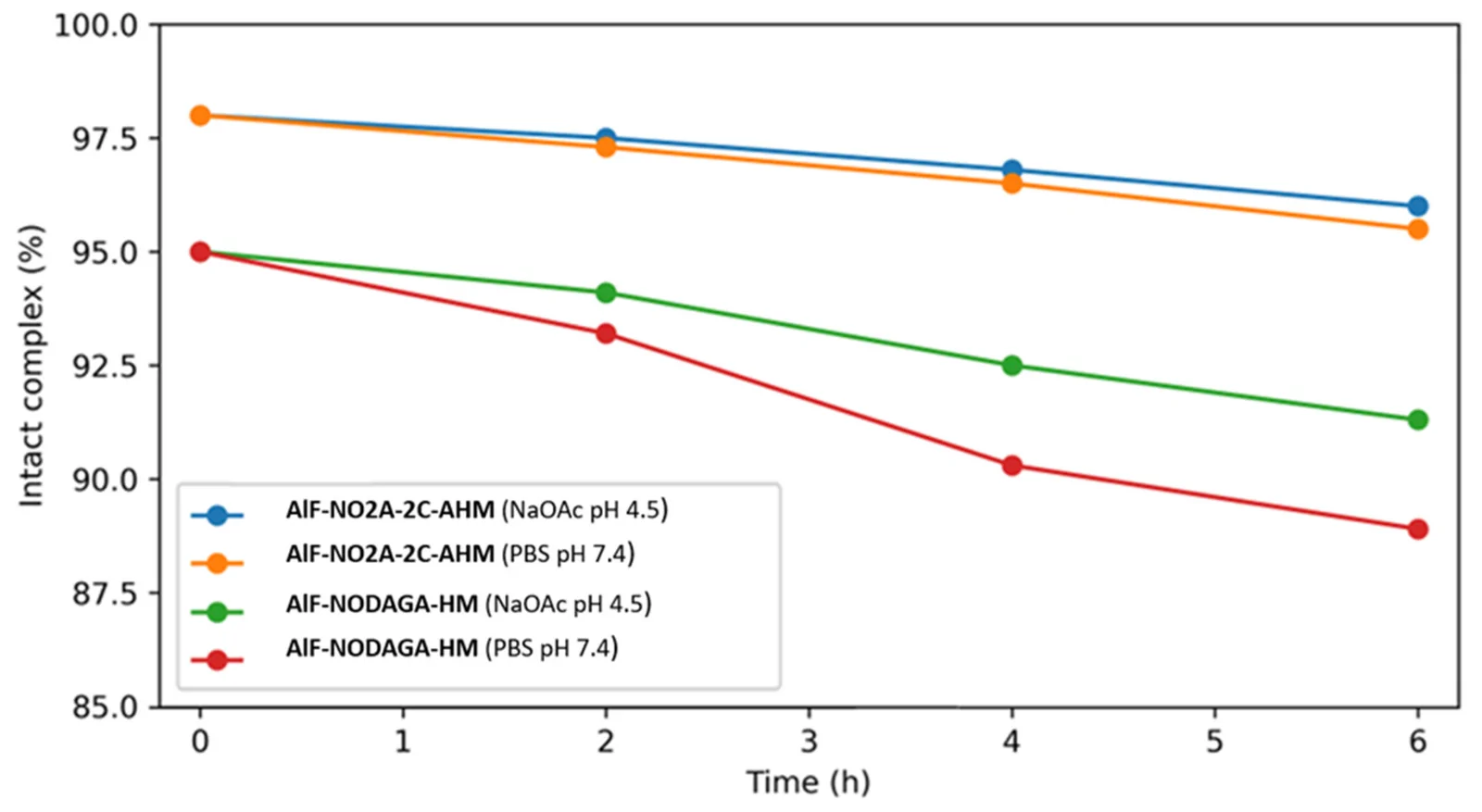
Stability profiles of **AlF-NO2A-2C-AHM** and **AlF-NODAGA-HM** (1 mg/mL) in 0.1 M NaOAc buffer (pH 4.5) and 0.1 M PBS (pH 7.4) at room temperature, determined by RP-HPLC monitoring at 214 nm over 6 h.

Finally, the stability of **AlF-NO2A-2C-AHM** and **AlF-NODAGA-HM** was evaluated in 0.1 M PBS (pH 7.4) and 0.1 M NaOAc buffer (pH 4.5) at room temperature. The complexes were monitored by RP-HPLC at 214 nm over 6 h, corresponding to approximately three half-lives of fluorine-18 (Figure 6). **AlF-NO2A-2C-AHM** exhibited excellent stability under both conditions, with more than 95% of the intact complex remaining after 6 h. **AlF-NODAGA-HM** also showed good stability, although a gradual decrease in the percentage of intact complex was observed, particularly in PBS, where approximately 89% of the complex remained after 6 h. Overall, both complexes retained a high level of integrity over the study period, indicating that {AlF}^2+^ coordination was largely preserved under the investigated conditions.

Although these results are encouraging within the imaging time window of fluorine-18, additional stability studies in biologically relevant media will be required to fully assess the suitability of these BFCs for radiopharmaceutical applications following conjugation to a targeting vector.

#### 2.2.2. Lutetium Complexation

Lutetium complexation was performed under conditions commonly used for NOTA-based ligands, namely in mildly acidic acetate buffer (pH 4.0–4.8), where deprotonation of the carboxylate groups is favored [31]. A slight excess of Lu^3+^ is generally used to promote complex formation. In our previous work on **NO2A-AHM** [21], efficient complexation was achieved in 0.1 M acetate buffer at pH 4.5 using mild heating (≈40 °C) and a reaction time of 30 min. These conditions were therefore applied to the newly synthesized chelators. Under these conditions, **NO2A-2C-AHM** did not show measurable complexation with Lu^3+^. The structural factors responsible for this behavior were further investigated by molecular modeling and are discussed in Section 2.3.2. In contrast, **NODAGA-HM** successfully coordinated Lu^3+^, affording a conversion rate of 95%. This behavior can be rationalized by the distinct coordination requirements of the {AlF}^2+^ and Lu^3+^. While the relatively compact {AlF}^2+^ species is readily complexed within the NOTA-derived coordination cavity of both ligands, Lu^3+^ possesses a significantly larger ionic radius and therefore requires a more favorable spatial arrangement of donor atoms for efficient complexation. In **NO2A-2C-AHM**, extension of the linker increases the distance between the additional acetate donor arm and the macrocyclic ring, limiting its effective involvement in metal coordination. As a result, the ligand behaves essentially as a NOTA-like chelator, which does not provide a sufficiently optimized coordination environment for Lu^3+^. In contrast, positioning the additional acetate donor arm closer to the macrocycle in **NODAGA-HM** creates a more preorganized and complementary coordination environment, thereby enabling efficient complexation of the Lu^3+^ ion.

After RP-HPLC purification under mildly acidic conditions (0.01% TFA in H_2_O/CH_3_CN), **Lu-NODAGA-HM** was isolated in 70% yield. The coordination behavior of both ligands was further investigated by computational analysis (Section 2.3.2).

**Figure 7:**
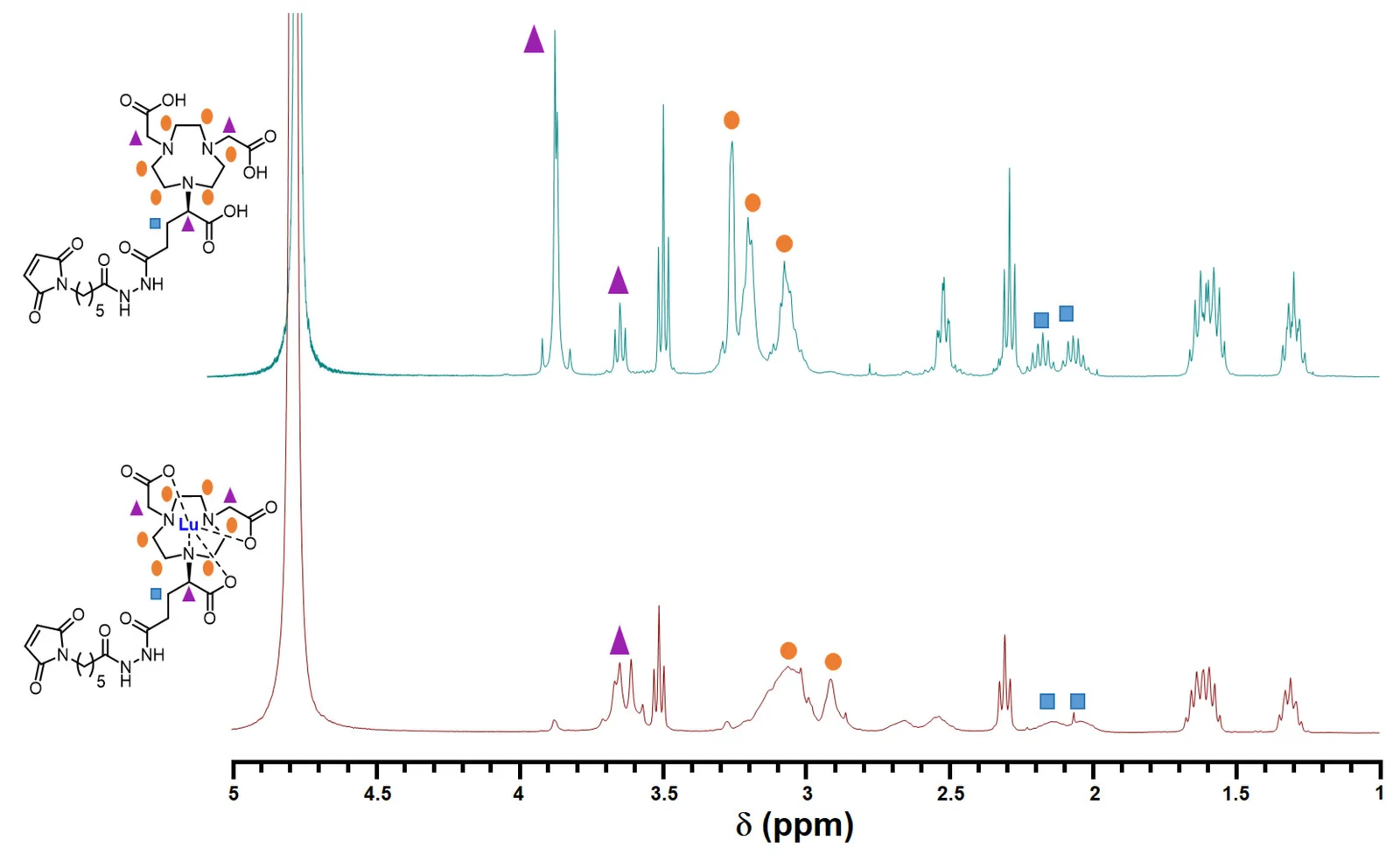
^1^H NMR spectra of **Lu-NODAGA-HM** and **NODAGA-HM** in D_2_O at 330 K (400 MHz).

Complex formation was further confirmed by ^1^H NMR spectroscopy (Figure 7). Upon coordination of Lu^3+^, significant modifications were observed in the spectral region associated with the chelating framework. In particular, the signals corresponding to the 12 protons of the NOTA macrocycle, initially located between 3.09 and 3.28 ppm, exhibited pronounced chemical-shift variations together with changes in multiplicity and signal duplication, reflecting the loss of proton equivalence upon metal coordination. Additional perturbations were observed for the methylene protons located in the α- and β-positions relative to the hydrazide function. The singlet assigned to the methylene groups of the acetate arms (3.88 ppm) and the triplet corresponding to the proton in the γ-position relative to the hydrazide carbonyl were also affected by complex formation. Notably, these two resonances merged into a single signal with altered resolution after coordination. These spectral modifications are consistent with substantial changes in the electronic environment of the chelator upon Lu^3+^ binding and confirm formation of **Lu-NODAGA-HM**.

### 2.3. Computational Analysis

To gain deeper molecular insights into the coordination chemistry of the newly synthesized BFCs and to rationalize the striking divergence observed during the experimental complexation studies, Density Functional Theory (DFT) calculations were performed. While experimental data in Section 2.2 demonstrated that both ligands readily accommodate the small {AlF}^2+^ core, a profound difference emerged regarding the larger Lu^3+^ ion, which was exclusively chelated by **NODAGA-HM**. Therefore, the structures of the AlF complexes were modeled to confirm geometric conservation, followed by an in-depth energetic analysis of the lutetium complexes to unravel the structural and energetic factors preventing complex formation with **NO2A-2C-AHM** in aqueous media.

#### 2.3.1. Aluminum-Fluoride Complexes

In line with previous computational investigations on **NO2A-AHM**, the coordination behavior of Al^3+^ was initially modeled by evaluating the relative stabilities of several binding arrangements. Among the various spatial conformations screened, the fluoride-containing octahedral complex—wherein the Al^3+^ center is coordinated by the three nitrogen atoms of the NOTA macrocycle, two carboxylate oxygen atoms, and one fluoride ion (N_3_O_2_F coordination sphere)—was confirmed as the energetic global minimum. This geometry proved significantly more stable than alternative hexacoordinated conformers lacking direct fluoride coordination, strongly correlating with literature data. Consequently, this optimized N_3_O_2_F octahedral core was selected as the structural blueprint for modeling the new derivatives, **NO2A-2C-AHM** and **NODAGA-HM** [21].

**Figure 8:**
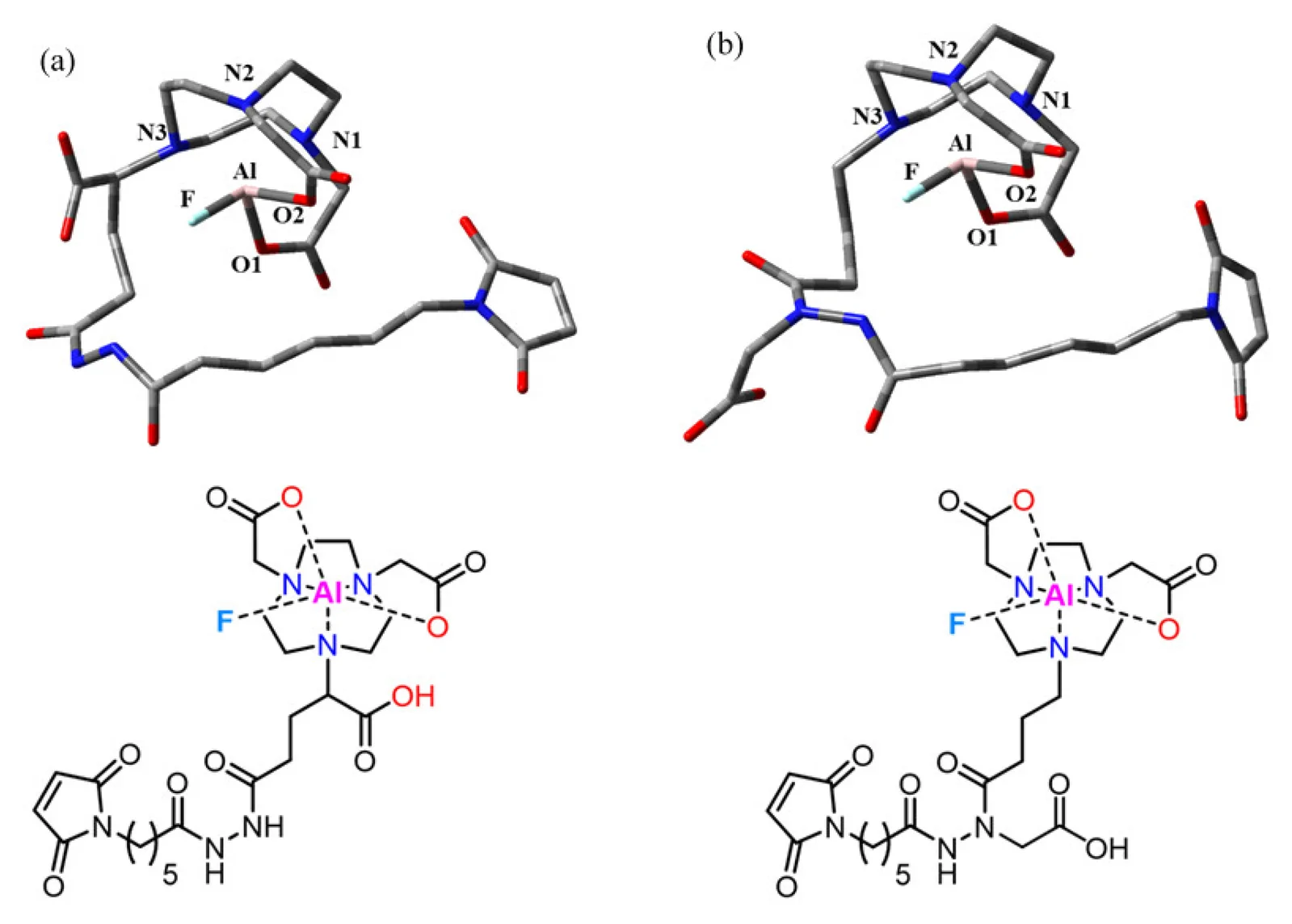
Optimized geometries of (**a**) **AlF-NODAGA-HM** and (**b**) **AlF-NO2A-2C-AHM**.

Geometric optimization confirmed that both novel BFCs perfectly preserve the pristine coordination environment of the parent **NO2A-AHM** scaffold. In both optimized complexes, the Al^3+^ cation resides centrally within the macrocyclic cavity, forming a distorted octahedral geometry with the six-coordinate N_3_O_2_F first coordination sphere (Figure 8). The calculated structural parameters, summarized in Table S1 (See Supporting Information), show that **NO2A-2C-AHM** exhibits an average Al–N bond length of 2.093 Å, an average Al–O distance of 1.821 Å, and a highly compressed Al–F bond at 1.703 Å. Similarly, the optimized framework of **NODAGA-HM** displayed average Al–N, Al–O, and Al–F distances of 2.182, 1.869, and 1.704 Å, respectively. These values closely match those previously reported for **NO2A-AHM** (Al–N = 2.130 Å, Al–O = 1.853 Å, and Al–F = 1.733 Å), demonstrating that the structural alterations introduced into the ligand framework exert negligible structural stress on the primary coordination capsule. Interestingly, the slightly shorter Al-N and Al-O distances calculated for **NO2A-2C-AHM** suggest a marginally stronger interaction with the {AlF}^2+^ core. Across all three chelators, the Al–F bond remains the shortest and most robust vector, driving the high thermodynamic affinity of fluoride for Al^3+^. The structural distortion of the octahedral envelope was further quantified by analyzing the twelve orthogonal and three linear angles around the aluminum center. For **NO2A-2C-AHM**, the average orthogonal and linear angles were 89.75° and 168.10°, respectively, while **NODAGA-HM** yielded values of 89.70° and 164.71°. These angular deviations from the ideal octahedral geometry (90° and 180°) are inherent to the rigidity of the triazacyclononane (TACN) ring and the spatial constraints imposed by the interlocking five-membered chelate rings, mirroring the parameters of the parent **NO2A-AHM** (89.81° and 168.33°).

#### 2.3.2. Lutetium Complexes

While the structural adaptability of the ligands allowed for uniform behavior with the {AlF}^2+^ core, experimental challenges arose when dealing with the larger Lu^3+^ ion, where **NO2A-2C-AHM** failed to provide any measurable complexation. To unlock the molecular rationale behind this selective behavior, DFT calculations were performed to evaluate the interaction energies (E_int_) of the respective lutetium complexes, explicitly contrasting gas-phase affinity with aqueous solvation using the SMD implicit solvation model.

The investigation into the complexation failure of **NO2A-2C-AHM** revealed a compelling thermodynamic narrative. In the gas phase, the optimized complex possesses a remarkably deep interaction energy (E_int_ = −1257.40 kcal·mol^−1^), confirming that **NO2A-2C-AHM** possesses a strong, intrinsic electrostatic and coordination affinity for the Lu^3+^ ion. However, upon the inclusion of explicit solvent effects, the interaction energy drops significantly to −1199.99 kcal·mol^−1^. This severe destabilization represents a significant solvation penalty of approximately 57.4 kcal·mol^−1^, demonstrating that the aqueous environment considerably weakens the metal–ligand interaction. In water, the dense hydration shell of the highly hydrophilic Lu^3+^ ion outcompetes the coordination capability of the ligand. The strong hydration of the Lu^3+^ ion in aqueous solution significantly weakens the metal–ligand interaction, preventing the formation of a sufficiently stable complex. This result is fully consistent with the experimental observation that **NO2A-2C-AHM** does not chelate Lu^3+^ under the investigated conditions.

**Figure 9:**
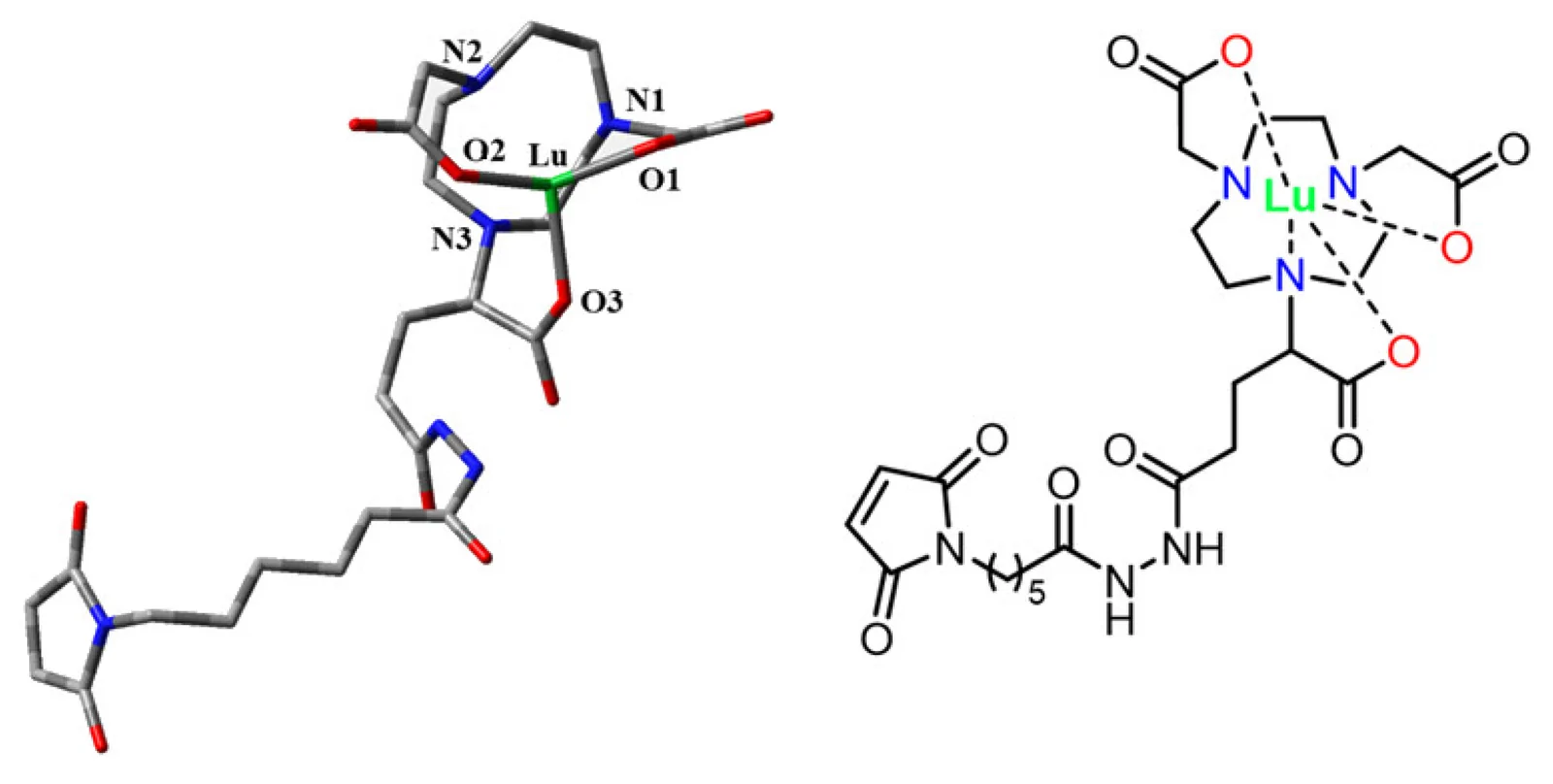
Optimized geometry of hexacoordinated **Lu-NODAGA-HM**.

Applying the exact same computational framework to **NODAGA-HM** highlighted a stark difference in their thermodynamic profile. In the gas phase, the optimized Lu^3+^ complex displayed a comparable interaction energy of −1256.65 kcal·mol^−1^. Crucially, upon transitioning to water, the interaction energy remained exceptionally robust at −1249.50 kcal·mol^−1^, resulting in a minimal solvation penalty of merely 7.15 kcal·mol^−1^. This limited destabilization clearly indicates that **Lu–NODAGA-HM** features superior structural pre-organization; by effectively coordinating the Lu^3+^ ion, the ligand successfully mitigates the competitive effects of water molecules, ensuring efficient complex formation as observed experimentally. To identify the preferred coordination mode of the successful **Lu–NODAGA-HM** complex, two initial structural arrangements were evaluated to accommodate the high coordination numbers typical of lanthanides: a hexacoordinated model (comprising the three NOTA nitrogen atoms and three carboxylate oxygen donor arms) (Figure 9) and a heptacoordinated model, which additionally forced the amide carbonyl oxygen into the first coordination sphere. Interestingly, geometric optimization of the heptacoordinated starting structure did not lead to a stable complex; instead, the metal-carbonyl distance spontaneously elongated, relaxing back to the exact same hexacoordinated structure obtained from the first model. This convergence indicates that the hexacoordinated envelope is heavily favored energetically, and that coordination of the amide carbonyl is not thermodynamically sufficient to overcome the geometric strain required to stabilize a heptacoordinated Lu^3+^ complex within this specific framework.

Finally, the precise structural parameters of the optimized hexacoordinated **Lu–NODAGA-HM** architecture were compared with the reference data of **Lu–NO2A-AHM** [21]. Both systems utilize a six-coordinate N_3_O_3_ core. The average Lu–N bond length was slightly shorter for **NODAGA-HM** (2.49 Å) than for **NO2A-AHM** (2.53 Å), suggesting a tighter, more intimate fit within the macrocyclic cavity. Similarly, the average Lu–O distances were shorter for **NODAGA-HM** (2.11 Å vs. 2.15 Å). As anticipated, these distances are systematically longer than those of the corresponding Al^3+^ complexes, a direct consequence of the significantly larger ionic radius of the lanthanide ion.

Angular analysis of the **Lu–NODAGA-HM** coordination polyhedron yielded an average orthogonal angle of 84.52° and a linear angle of 140.58°. Compared to **Lu–NO2A-AHM** (89.48° and 142.39°), **NODAGA-HM** induces a more severe geometric distortion away from the ideal octahedral architecture. This increased distortion is a structural consequence of the relocated donor arm and the tight conformational constraints of the ligand framework. Nevertheless, the highly efficient experimental chelation observed for **NODAGA-HM** despite its greater geometric distortion suggests that efficient coordination of large lanthanide ions depends primarily on the ability of the ligand to provide an appropriate coordination environment rather than on maintaining an ideal, symmetric octahedral geometry. This interpretation is consistent with the improved accommodation of the larger Lu^3+^ ion afforded by the additional acetate donor arm.

### 2.4. Radiolabeling Studies

The radiolabeling performance of the newly synthesized BFCs was subsequently evaluated using Al^18^F and ^177^Lu. Radiolabeling efficiency was assessed by determining the radiochemical conversion (RCC), defined as the percentage of radionuclide successfully incorporated into the desired radiocomplex. Prior non-radioactive complexation studies demonstrated that while both ligands efficiently coordinated the {AlF}^2+^ core, only **NODAGA-HM** formed a stable complex with Lu^3+^. Based on these chemical observations, radiolabeling investigations were undertaken to validate the suitability of both chelators for PET imaging and targeted radionuclide therapy applications.

#### 2.4.1. Al^18^F Radiolabeling

The Al^18^F radiolabeling of NOTA-derived ligands has been extensively investigated since its pioneering introduction by McBride and co-workers [9]. Subsequent studies have established that the RCC is strongly driven by both the reaction pH and the presence of hydrophilic organic co-solvents. In particular, the use of ethanol at concentrations above 50/50 (*v*/*v*) significantly improves complexation kinetics, while buffered solutions in the pH range of 4.5–5.0 are commonly preferred for Al^18^F-radiolabeling [32,33,34].

**Figure 10:**
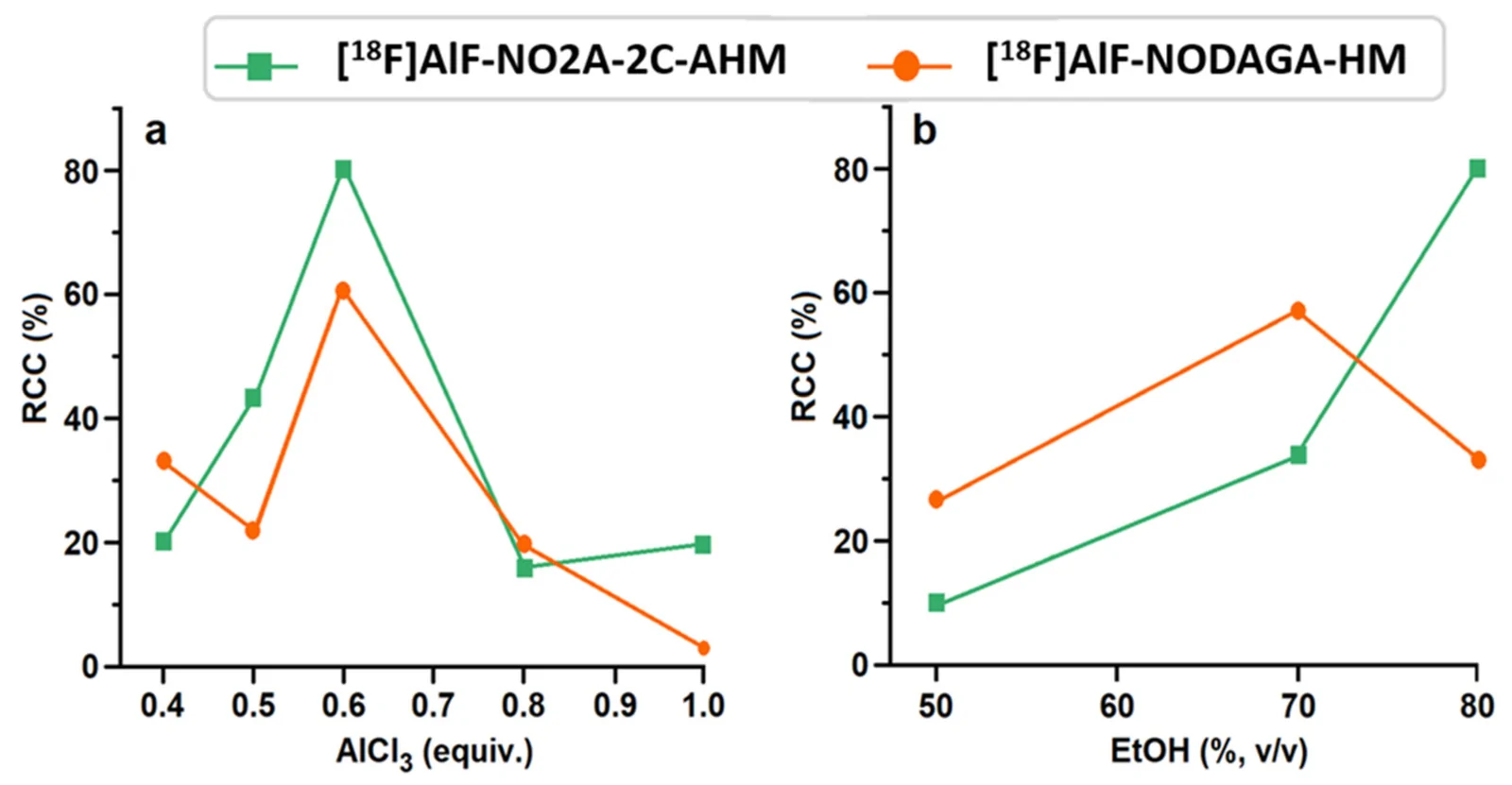
Effect of radiolabeling conditions on the RCC of **[^18^F]AlF-NO2A-2C-AHM** and **[^18^F]AlF-NODAGA-HM**. (**a**) Effect of the AlCl_3_-to-ligand ratio. (**b**) Effect of ethanol content (n = 1).

In the present work, the radiolabeling strategies for **NODAGA-HM** and **NO2A-2C-AHM** were carefully adapted from the reaction conditions previously established by our group for **NO2A-AHM** [21]. Radiolabeling was performed by first producing [^18^F]NaF, which was subsequently reacted with an AlCl_3_ solution (0.4–1.0 equiv.) in 0.5 M NaOAc buffer (pH 4.5) containing EtOH (50–80%, *v*/*v*) for 5 min at room temperature. The BFCs (50 µg, 1 equiv.) were then added, and the resulting reaction mixture was heated at 90 °C for 15 min. To identify the optimal labeling conditions for each chelator, the effects of both the AlCl_3_ amount and the EtOH content on the overall radiolabeling efficiency were systematically investigated. For both ligands, the AlCl_3_-to-ligand ratio exerted a marked influence on the RCC, with the highest conversions obtained using 0.6 equiv. of AlCl_3_. Under these optimized conditions, the RCC reached 59 ± 2% (n = 3) for **NODAGA-HM** and 75 ± 4% (n = 3) for **NO2A-2C-AHM** (Figure 10a). The influence of the EtOH content was subsequently evaluated (Figure 10b). While increasing the proportion of EtOH significantly improved the RCC for both ligands, distinct optimal thresholds were observed. The highest conversion was achieved at 80% EtOH for **NO2A-2C-AHM**, whereas **NODAGA-HM** reached its maximum conversion at 70% EtOH. Throughout this optimization study, only the AlCl_3_-to-ligand ratio and EtOH content were varied, while the precursor amount, reaction temperature, reaction time, and buffer pH were strictly kept constant. The optimized conditions for complexation of **NODAGA-HM** with Al-^18^F, consisting of 0.5 M sodium acetate buffer (NaOAc, pH 4.5) containing 70% (*v*/*v*) ethanol and a reaction temperature of 90 °C, provided RCC values of 59 ± 2% (n = 3), with an average molar activity (A_m_) of 0.60 ± 0.1 MBq/nmol;for **NO2A-2C-AHM** the optimized conditions consisted of 0.5 M sodium acetate buffer (NaOAc, pH 4.5) containing 80% (*v*/*v*) ethanol and a reaction temperature of 90 °C. Under these conditions, RCC values of 75 ± 4% (n = 3) were obtained, with an average molar activity (A_m_) of 0.60 ± 0.1 MBq/nmol calculated from the ratio between the total activity and the molar amount of BFC used for radiosynthesis.

Although the RCC values obtained for **NODAGA-HM** and **NO2A-2C-AHM** are lower than those reported for certain optimized NOTA-based systems, they remain sufficient for proof-of-concept radiolabeling studies and allowed reliable radiochemical characterization of both compounds. Since these bifunctional chelators are intended for subsequent conjugation to tumor-targeting peptides, further optimization of additional radiolabeling parameters, such as precursor amount, reaction time, and temperature, will be performed on the final peptide conjugates, as these parameters may differ after bioconjugation.

**Figure 11:**
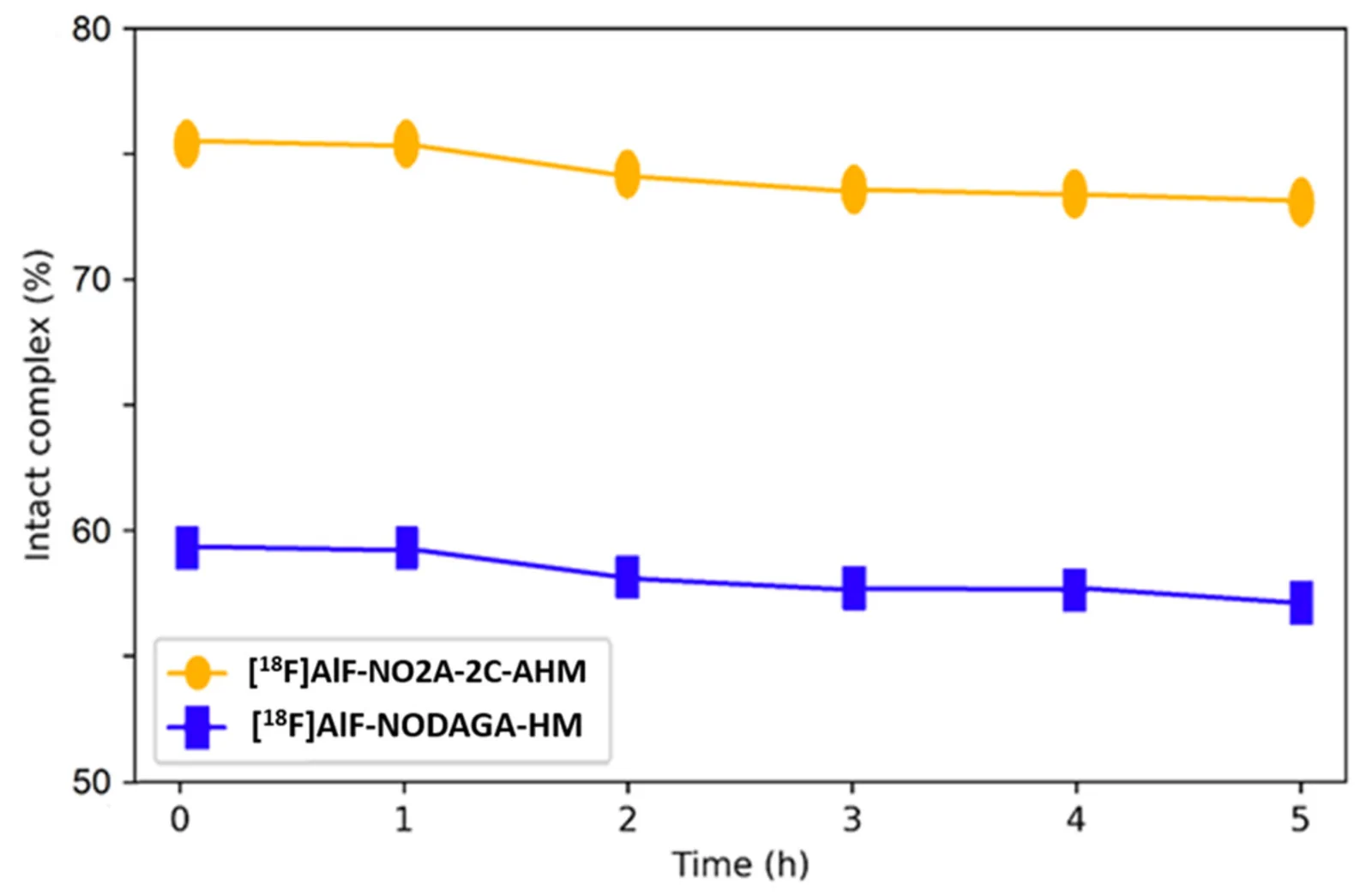
Stability profiles of **[^18^F]AlF-NO2A-2C-AHM** and **[^18^F]AlF-NODAGA-HM** at room temperature over 5 h determined by radio-HPLC.

The stability of the newly formed Al^18^F-complexes was evaluated by radio-HPLC at room temperature over a period of 5 h. Both radiocomplexes exhibited excellent stability throughout the study, with only a marginal decrease in RCC corresponding to approximately 4% of the initially formed complexes (Figure 11). Crucially, no additional radioactive species or degradation products were detected during the monitoring period, indicating that both **[^18^F]AlF-NO2A-2C-AHM** and **[^18^F]AlF-NODAGA-HM** remained stable under the investigated conditions.

#### 2.4.2. ^177^Lu Radiolabeling

**Figure 12:**
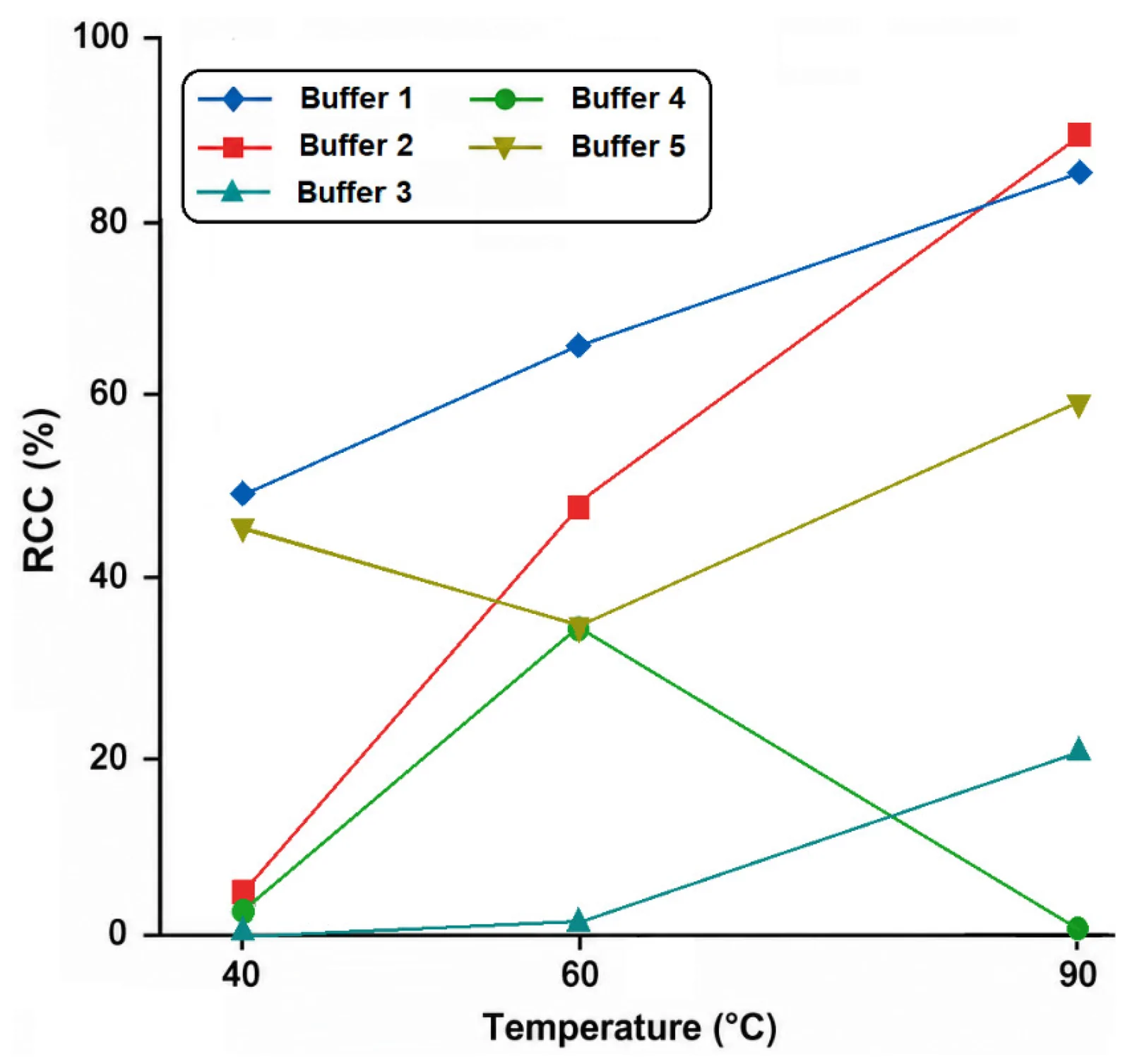
Effect of reaction temperature on the RCC of **[^177^Lu]Lu-NODAGA-HM** using different reaction media: Buffer 1 (0.7 M NaOAc, pH 8.0), Buffer 2 (0.7 M NaOAc, pH 4.5), Buffer 3 (0.03 M NaOAc, pH 4.5, EtOH), Buffer 4 (0.7 M NaOAc, pH 4.5, 0.56 M NaAsc), and Buffer 5 (1.5 M NaOAc, pH 6.0, 0.04 M ascorbic acid, AcOH, EtOH) (n = 1).

Based on the non-radioactive complexation studies described above, **NO2A-2C-AHM** was expected to exhibit poor coordination properties toward Lu^3+^. To confirm this behavior under radiolabeling conditions, five different reaction media were thoroughly evaluated (Figure 12). Despite these optimization attempts, no measurable RCC was obtained at 90 °C under any of the investigated conditions. These results are fully consistent with the non-radioactive complexation experiments and further confirm the inability of the **NO2A-2C-AHM** scaffold to efficiently coordinate ^177^Lu. In stark contrast, replacing the NO2A core with a NODAGA framework resulted in a marked improvement in radiolabeling performance. **NODAGA-HM** was therefore evaluated under the exact same experimental conditions to identify the optimal parameters for ^177^Lu radiolabeling.

Among the investigated reaction media (Figure 12), the highest RCC (87.3 ± 1.5% (n = 3)) was obtained in Buffer 2 (0.7 M NaOAc, pH 4.5), while Buffer 1 (0.7 M NaOAc, pH 8.0) afforded a slightly lower RCC (85%). This observation is consistent with the well-established influence of pH on lanthanide complexation, as both the protonation state of the chelator and the hydrolysis equilibrium of Lu^3+^ strongly dictate coordination efficiency. Nevertheless, the relatively high RCC obtained at pH 8.0 indicates that **NODAGA-HM** retains a significant ability to chelate ^177^Lu over a relatively broad pH range. The influence of the reaction medium was further investigated using Buffers 3–5 (Figure 12). Although ethanol has previously been reported to enhance the ^177^Lu radiolabeling efficiency of several radiopharmaceuticals [34,35,36]—enabling, for instance, the rapid preparation of [^177^Lu]Lu-PSMA-617 at room temperature within 10 min [34]—its evaluation in the present study did not yield similar improvements. Contrary to these literature reports, the addition of ethanol (Buffer 3) resulted in lower RCC values than those obtained under the optimal labeling conditions (Buffer 2), indicating that ethanol does not promote ^177^Lu-complexation with **NODAGA-HM**. Similarly, the addition of sodium ascorbate (NaAsc, Buffer 4) negatively affected radiolabeling efficiency. These conditions, adapted from the EasyOne radiolabeling protocol for [^177^Lu]Lu-PSMA-I&T, resulted in poor RCC, suggesting either competitive interactions or an unfavorable coordination environment for ^177^Lu complex formation. Likewise, the combination of ascorbic acid, acetic acid, and EtOH (Buffer 5) did not improve radiolabeling performance, leading to lower RCC values than those obtained with Buffer 2. Temperature optimization further confirmed the robustness of the selected labeling conditions. Increasing the reaction temperature from 40 °C to 90 °C markedly enhanced the RCC, with the highest conversion consistently achieved at 90 °C in Buffer 2. It is important to note that the analysis of crude mixtures heated at 90 °C showed no by-product identification of BFC in both UV and radioactive HPLC detection. In contrast, the alternative reaction media remained significantly less effective over the entire temperature range investigated. These results clearly demonstrate that an elevated temperature is required to promote rapid and efficient complexation of ^177^Lu by **NODAGA-HM** (Figure 12). Although a reaction temperature of 90 °C may appear high for certain heat-sensitive biomolecules, the present study was intentionally performed on the unconjugated bifunctional chelator in order to establish its intrinsic radiolabeling properties. Once conjugated to a targeting peptide, the radiolabeling conditions will be re-optimized to identify the mildest conditions compatible with both efficient complexation and preservation of biomolecule integrity.

**Figure 13:**
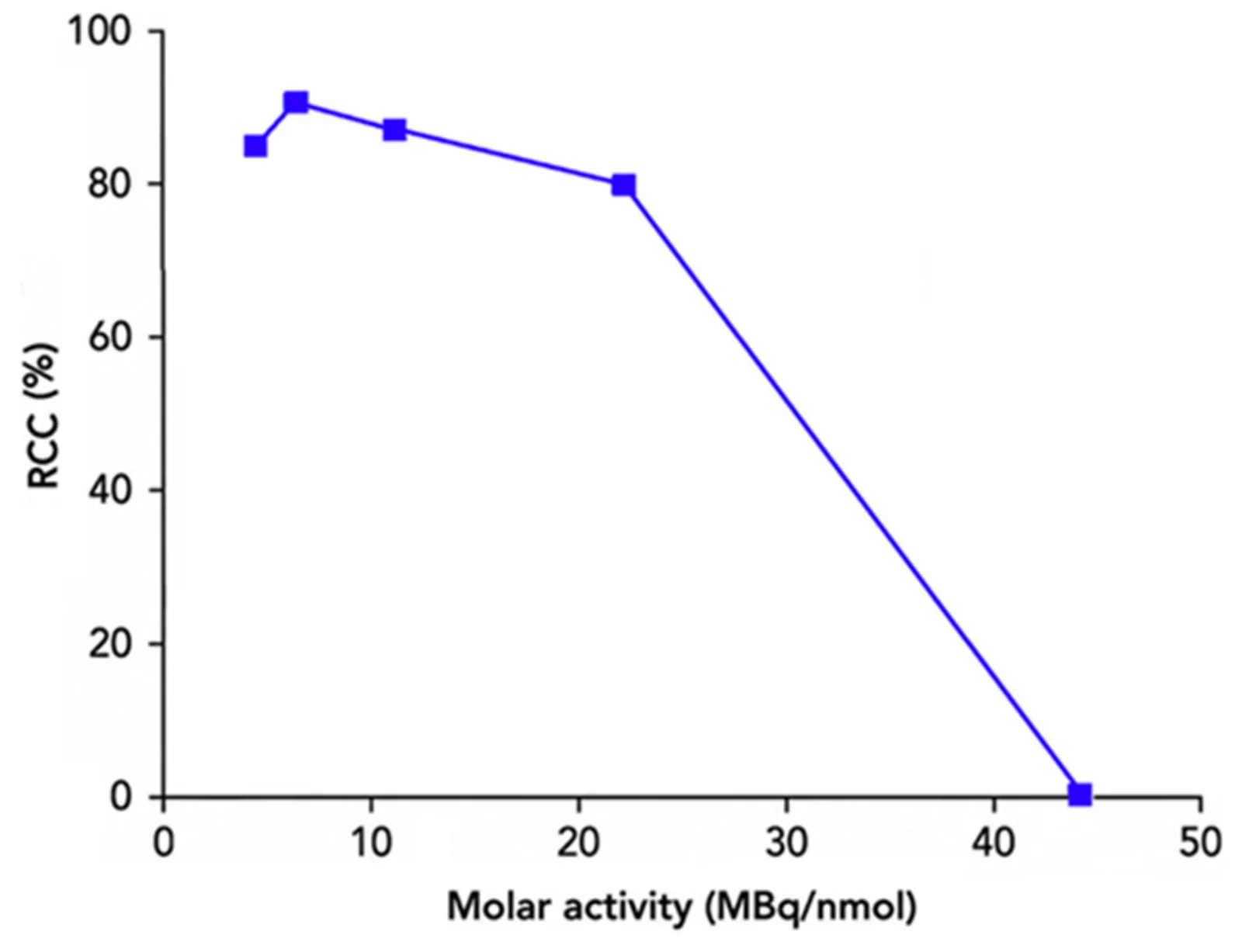
Effect of molar activity on the RCC of **[^177^Lu]Lu-NODAGA-HM** (n = 1).

Under these optimized labeling conditions, the influence of the precursor amount, reflected by the resulting molar activity, was subsequently evaluated. High RCC values (85–90%) were maintained at low to moderate molar activities, corresponding to higher precursor amounts. In contrast, increasing the molar activity by reducing the precursor amount led to a marked decrease in RCC. At a molar activity of 45 MBq·nmol^−1^, the RCC dropped to 0%. This decrease is attributed to the reduced amount of precursor used in the radiolabeling reaction rather than to an intrinsic limitation of the chelator itself, since the radionuclide activity remained constant throughout the experiment. These results demonstrate that maintaining a sufficient precursor amount is essential for achieving high radiolabeling efficiency under the investigated conditions. Since the present study was primarily intended to compare the intrinsic radiolabeling behavior of the BFCs, further optimization of the precursor amount will be performed after conjugation to a targeting peptide, as the optimal radiolabeling conditions may differ for the final radiopharmaceutical (Figure 13).

The poor radiolabeling performance observed for the NO2A-based analogs (**NO2A-AHM** [21] and **NO2A-2C-AHM**) likely reflects their lower thermodynamic stability and/or slower complexation kinetics toward ^177^Lu compared with the NODAGA scaffold. Overall, these results clearly demonstrate the superiority of the **NODAGA-HM** platform for ^177^Lu radiolabeling. The optimized conditions, consisting of Buffer 2 (0.7 M sodium acetate buffer, pH 4.5) and a reaction temperature of 90 °C, provided high and highly reproducible RCC values of 87.3 ± 1.5% (n = 3) with an average A_m_ = 6.32 ± 0.08 MBq/nmol calculated from the ratio between the total activity and the molar amount of NODAGA-HM used for radiosynthesis. Thus, **NODAGA-HM** was identified as the most promising candidate for further radiopharmaceutical development.

## 3. Materials and Methods

### 3.1. Materials

#### 3.1.1. Synthesis

Unless otherwise stated, all chemicals were purchased at the highest commercially available purity and used without further purification. Anhydrous solvents were obtained either by distillation over P_2_O_5_ under aan argon atmosphere or by storage over activated molecular sieves. All other reagent-grade solvents were used as received. NO2A-*t*Bu and NODAGA-NHS were obtained from CheMatech (Dijon, France). 2-(*1H*-7-Azabenzotriazol-1-yl)-1,1,3,3-tetramethyluronium hexafluorophosphate (HATU) was purchased from Iris Biotech GmbH (Marktredwitz, Germany). Hydrazine monohydrate, *tert*-butyl carbazate, *N*-methylmorpholine (NMM), trifluoroacetic acid (TFA), and Lu(NO_3_)_3_ were obtained from Alfa Aesar (Haverhill, MA, USA). 6-Maleimidohexanoic acid, *tert*-butyl bromoacetate, potassium carbonate, sodium fluoride (NaF), aluminum chloride (AlCl_3_) and 4-bromobutyric acid were purchased from Sigma-Aldrich (Taufkirchen, Germany). 2,6-lutidine was obtained from Acros Organics (Geel, Belgium). 4-bromobutanoyl chloride was purchased from TCI Europe (Eschborn, Germany). Milli-Q water (18.2 MΩ·cm) was systematically used for the preparation of all aqueous solutions. Unless otherwise specified, all synthetic steps were performed under a dry nitrogen atmosphere.

Centrifugation was carried out using an Eppendorf 5810 R centrifuge (Eppendorf SE, Hamburg, Germany). Flash column chromatography was performed on Geduran 60 H silica gel (63–200 mesh) (Merck KGaA, Darmstadt, Germany). Preparative HPLC was conducted on a Shimadzu Prominence system equipped with an LC-20AT pump, an SPD-M20A photodiode array (PDA) detector, and a CTO-20A column oven (Shimadzu Corporation, Kyoto, Japan), a Varian Pursuit5^®^ reversed-phase column (5 µm, 21.2 mm × 150 mm Varian; Agilent Technologies, Santa Clara, CA, USA). Data acquisition was managed using Varian Star Chromatography software, version 5.54 SP2. The preparative column was operated at a constant flow rate of 10 mL·min^−1^ with an injection volume of 10 mL, and the purification was monitored at 308 nm. Mobile phase A consisted of H_2_O containing 0.1% *v*/*v* TFA, and mobile phase B consisted of CH_3_CN containing 0.1% *v*/*v* TFA.

Analytical HPLC-MS analyses were performed on a Shimadzu LC-MS-2020 system equipped with a diode array detector (SPD-M20A) and an electrospray ionization (ESI) mass spectrometry detector (Shimadzu, Marne-la-Vallée, France). Chromatographic separation was achieved on a Pursuit 5^®^ C18 column (150 mm × 4.6 mm, 5 µm, Varian; Agilent Technologies, Santa Clara, CA, USA). Mobile phase A consisted of CH_3_CN/H_2_O (5/95, *v*/*v*) containing 0.1% *v*/*v* formic acid, whereas mobile phase B consisted of CH_3_CN containing 0.1% *v*/*v* formic acid. The column was operated at a flow rate of 0.8 mL·min^−1^ using a linear gradient from 5% to 100% B over 15 min. Mass spectra were acquired in positive ESI mode (ESI^+^).

One-dimensional (^1^H, ^13^C-JMOD, and ^19^F) and two-dimensional (COSY, HSQC, and HMBC) NMR spectra were recorded on a Bruker Avance 400 MHz spectrometer at 298 K or 330 K using CDCl_3_, DMSO-*d*_6_, or D_2_O as deuterated solvents at the LRGP NMR facility, Université de Lorraine (Nancy, France). In the ^13^C-JMOD (J-modulated) spectrum, the multiplicity of the ^13^C signals (methyl: quartet, methylene: triplet, methine: doublet, quaternary: singlet) is reflected in the phase of the fully ^1^H decoupled ^13^C spectrum using the SEFT (Spin-Echo Fourier Transform) technique. In the spectrum, the CH and CH_3_ signals give signals with a positive phase, while the CH2 and quaternary carbon atoms give negative phase signals. Chemical shifts (*δ*) are reported in parts per million (ppm) relative to the residual solvent signals: CDCl_3_ (*δ*_H_ = 7.26 ppm, *δ*_C_ = 77.16 ppm), DMSO-*d*_6_ (*δ*_H_ = 2.50 ppm, *δ*_C_ = 39.52 ppm), or D_2_O (*δ*_H_ = 4.79 ppm). Spin-spin coupling constants (*J*) are expressed in hertz (Hz). Signal multiplicities are designated as follows: s (singlet), d (doublet), t (triplet), m (multiplet), br (broad), or combinations thereof, H* (exchangeable proton), H_Ar_ (aromatic protons), C_Ar_ (aromatic carbons).

High-resolution mass spectrometry (HRMS) analyses were performed on a Bruker micrOTOF instrument using ESI^+^ mode, scanning mass ranges of *m*/*z* 50–1000 in low-range mode and *m*/*z* 50–2500 in wide-range mode.

#### 3.1.2. Radiolabeling

A 0.9% (*w*/*v*) sodium chloride injectable solution was obtained from B. Braun (Saint-Cloud, France). Anhydrous AlCl_3_ (99.999%, trace metals basis) and anhydrous NaOAc (99.99%, trace metals basis) were purchased from Sigma-Aldrich (Taufkircher, Germany), and glacial acetic acid (>99.85%) was supplied by Merck (Darmstadt, Germany). Absolute anhydrous ethanol was acquired from Carlo Erba (Val-de-Reuil, France).

All reaction buffers were prepared, and their pH values were recorded using a Mettler Toledo pH meter (Mettler-Toledo GmbH, Greifensee, Switzerland) equipped with an InLab Micro pH electrode. Sep-Pak Accell Plus QMA Plus Light cartridges (130 mg sorbent loading, particle size 37–55 μm) were purchased from Waters Corporation (Milford, MA, USA).

No-carrier-added fluorine-18 was produced via the ^18^O(*p*,*n*)^18^F nuclear reaction using a GE Healthcare PETtrace cyclotron (GE Healthcare, Milwaukee, WI, USA). Irradiation was performed at 10 μA for 5 min, affording approximately 3.7 GBq of fluorine-18 in 1.6 mL of ^18^O-enriched water. Radiolabeling reactions were carried out using an Eppendorf ThermoMixer from Eppendorf for simultaneous heating and agitation. Manual radiolabeling following [^18^F]NaF production was carried out on an AllInOne^®^ (Trasis, Ans, Belgium) synthesis platform, both supplied by Trasis.

Radiosyntheses were monitored by radio-thin-layer chromatography (radio-TLC), radio-high-performance liquid chromatography (radio-HPLC) and radio-ultrahigh-performance liquid chromatography (radio-UHPLC). Radio-TLC analyses were performed using a mini-GITA^®^ TLC scanner from Elysia-Raytest (Straubenhardt, Germany). Radio-HPLC analyses were carried out on a Waters system equipped with a 2695eb pump, an autosampler, and a 2998 PDA detector, coupled to a NaI radioactivity detector from Berthold (Bad Wildbad, Germany), and operated with Empower software version 3.6.1 (Orlando, FL, USA). Chromatographic separations were achieved on an ACE^®^ Avantor RP-C18 column (AIT, Cormeilles-en-Parisis, France) (150 × 3.0 mm, 3 μm). Radio-UHPLC analyses were performed using a Shimadzu UHPLC system (Shimadzu Corporation, Kyoto, Japan) equipped with UV and radiometric detectors. Data acquisition and processing were performed using LabSolutions software version 6 106 SP1. Mobile phase A consisted of water containing 0.1% *v*/*v* TFA, whereas mobile phase B consisted of CH_3_CN containing 0.1% *v*/*v* TFA. Detection was performed simultaneously by UV absorbance at 214 nm and radioactivity monitoring. Manual radiolabeling experiments with lutetium-177 were performed using [^177^Lu]LuCl_3_ supplied by Curium.

### 3.2. Organic Synthesis

#### 3.2.1. Tert-Butyl N-(4-bromobutanoyl)-N-(6-(2,5-dioxo-2,5-dihydro-1H-pyrrol-1-yl)hexanamido)Glycinate (**2**)

Compound **1** was synthesized according to the previously reported procedure developed by our group, without any modification to the experimental protocol or isolated yield [21]. Compound **1** was obtained from *tert*-butyl carbazate and 6-maleimidohexanoic acid in three steps and with an overall yield of 48% (Scheme 2).

To a solution of compound **1** (100 mg, 0.295 mmol, 1 equiv.) and 2,6-lutidine (69 µL, 0.590 mmol, 2 equiv.) in anhydrous DCM (1 mL), a solution of 4-bromobutyryl chloride (35 µL, 0.295 mmol, 1 equiv.) in anhydrous DCM (2 mL) was added dropwise over 10 min at room temperature. The reaction mixture was stirred for an additional 20 min and then washed with aqueous 0.1% *v*/*v* TFA (3 × 5 mL). The aqueous phases were extracted with DCM (2 × 10 mL), and the combined organic layers were dried over MgSO_4_, filtered, and concentrated under reduced pressure to afford compound **2** as a yellow oil (100 mg, 70%).

Analytical HPLC (5–100% B in 15 min, 0.8 mL·min^−1^) Rt = 19.7 min, purity: 98% at 214 nm.

^1^H NMR (400 MHz, CDCl_3_) *δ* 1.40–1.28 (m, 2H, N-CH_2_-CH_2_-C**H_2_**, maleimide linker), 1.46 (s, 9H, 3 C**H_3_**, *t*Bu), 1.67 (m, 4H, N-CH_2_-C**H_2_**-CH_2_-C**H_2_**, maleimide linker), 2.63–2.45 (m, 4H,C**H_2_**-CH_2_-Br, C**H_2_**-CO-N, maleimide linker), 2.54–2.51 (m, 2H,C**H_2_**-CH_2_-CH_2_-Br), 3.57–3.45 (m, 4H, C**H_2_**-Br, N-C**H_2_**-CH_2_, maleimide linker), 6.69 (s, 2H, C**H**=C**H**, maleimide core), 8.00 (s, 1H,N**H**).

^13^C NMR (100.6 MHz, CDCl_3_) *δ* 24.35 **C**H_2_-CH_2_-CO-N, maleimide linker), 26.05 (**C**H_2_-CH_2_-CH_2_-CO, maleimide linker), 27.36 (**C**H_2_-CH_2_-Br), 27.90 (3 **C**H_3_, *t*Bu), 29.81 (**C**H_2_-Br ), 33.41 (**C**H_2_-CH_2_-CH_2_-Br), 33.77 (**C**H_2_-CO-N, maleimide linker), 37.28 (N-**C**H_2_-CH_2_, maleimide linker), 48.62 (NH-**C**H_2_-CO_2_*t*Bu, additional arm), 82.72 (**C**, *t*Bu), 133.95 (H**C**=**C**H), 168.88 (CH_2_-**C**O_2_), 170.72 (2 **C**O), 171.28 (**C**ONH), 173.93 (**C**ONH).

HPLC-MS (ESI^+^) *m*/*z* calcd for C_20_H_30_BrN_3_O_6_ [M + Na]^+^: 510.12; found: 510.10.

#### 3.2.2. Di-Tert-Butyl 2,2′-(7-(1-(6-(2-(4-bromobutanoyl)-2-(2-(tert-butoxy)-2-oxoethyl)hydrazineyl)-6-oxohexyl)-2,5-dioxopyrrolidin-3-yl)-1,4,7-triazonane-1,4-diyl)Diacetate (**3**)

To a solution of compound **2** (89 mg, 0.182 mmol, 1 equiv.) in anhydrous CH_3_CN (10 mL), a solution of NO2A*t*Bu (58.6 mg, 0.164 mmol, 0.9 equiv.) and DIPEA (63.4 µL, 0.364 mmol, 2 equiv.) in anhydrous CH_3_CN (4.5 mL) was added dropwise at room temperature. The reaction mixture was stirred at room temperature overnight, then diluted with water containing 0.1% *v*/*v* TFA (50 mL). The aqueous phase was extracted with DCM (3 × 50 mL), and the combined organic layers were washed with brine (30 mL), dried over MgSO_4_, filtered, and concentrated under reduced pressure. The crude residue was purified by preparative HPLC using a linear gradient from 50% to 70% B over 25 min (Rt = 15 min) to afford compound **3** as a yellow oil (69.2 mg, 50%).

Analytical HPLC (5–100% B in 15 min, 0.8 mL· min^−1^) Rt = 20.02 min, purity: 90% at 214 nm.

^1^H NMR (400 MHz, CDCl_3_) *δ* 1.38–1.29 (m, 2H, N-CH_2_-CH_2_-C**H_2_**, maleimide linker), 1.46 (s, 27H, 9 C**H_3_**, *t*Bu), 1.60–1.7 (m, 4H, N-CH_2_-C**H_2_**-CH_2_-C**H_2_**, maleimide linker), 2.9–2.1 (m, 20H, N-CH_2_-C**H_2_**-CH_2_-C**H_2_**, maleimide linker, C**H_2_**-C**H_2_**-CH_2_-Br, 3 N-C**H_2_**-C**H_2_**, macrocycle), 3.01–3.11 (m, 2H, N-C**H_2_**-CO_2_*t*Bu, additional arm), 3.1–3.7 (m, 8H, N-C**H_2_**-CH_2_, maleimide linker, C**H_2_**-Br, 2 N-C**H_2_**-CO_2_*t*Bu, macrocycle core), 4.13 (dd, 1H, *J* = 8.8, 5.6 Hz, C**H**-N-CH_2_-CH_2_-N, maleimide core).

^13^C NMR (100.6 MHz, CDCl_3_) δ 22.01 (**C**H_2_-CH_2_-CO-NH, maleimide linker), 24.60 (**C**H_2_-CH_2_-CH_2_-CO-NH, maleimide linker), 25.89 (CH_2_-**C**H_2_-CH_2_-Br), 26.99 (N-CH_2_-**C**H_2_, maleimide linker), 27.88 (9 **C**H_3_, *t*Bu), 29.85 (**C**H_2_-Br), 32.99 (**C**H_2_-CH_2_-CH_2_-Br), 38.17 (**C**H_2_, maleimide core), 38.61 (N-**C**H_2_-CH_2_, maleimide linker), 47.33 (N-**C**H_2_-CO_2_*t*Bu, additional arm), 53.78 (N-**C**H_2_-**C**H_2_, macrocycle core), 55.52 (N-**C**H_2_-**C**H_2_, macrocycle core), 55.94 (N-**C**H_2_-**C**H_2_, macrocycle core), 60.28 (**C**H-N-CH_2_, maleimide core) 63.84 (2 N-**C**H_2_-CO_2_*t*Bu, macrocycle core), 83.90 (3 **C**, *t*Bu), 159.87 (3 **C**O_2_*t*Bu), 165.87 (**C**O-NH), 174.71 (N-**C**O), 176.73 (2 **C**O).

HPLC-MS (ESI^+^) *m*/*z* calcd for C_38_H_65_BrN_6_O_10_ [M + H]^+^: 845.40; found: 845.40.

#### 3.2.3. (9H-fluoren-9-yl)Methyl Hydrazinecarboxylate (**4**)

Compound **4** was synthesized according to the procedure previously reported by Boeglin et al. [27], reproducing the experimental protocol without modification.

Briefly, to a stirred solution of hydrazine hydrate (19 g, 386 mmol, 10 equiv.) in CH_3_CN/H_2_O (150 mL, 1/1, *v*/*v*) cooled to 0 °C, a solution of Fmoc-Cl (10 g, 38.6 mmol, 1 equiv.) in CH_3_CN (600 mL) was added dropwise over 2.5 h. The reaction mixture was allowed to warm slowly to room temperature and stirred overnight. CH_3_CN was removed under reduced pressure, and compound **4** was precipitated by the addition of H_2_O (200 mL). The resulting solid was collected by filtration using a porosity 4 fritted glass filter, washed successively with H_2_O (100 mL) and hexane (50 mL), and dried under reduced pressure to afford compound **4** as a white powder (8.9 g, 91%) without requiring further purification. Spectroscopic data (^1^H NMR) were in perfect agreement with literature values [27].

Analytical HPLC (0–100% B in 30 min, 0.8 mL·min^−1^), Rt = 9.97 min, purity: 90% at 214 nm.

^1^H NMR (DMSO-*d*_6_, 400 MHz) *δ* 4.10 (s, 2H, N**H_2_**), 4.23 (d, 1H, *J* = 6.9 Hz, C**H**), 4.29 (d, 2H, *J* = 6.6 Hz, C**H_2_**), 7.30 (t, 2H, *J* = 7.5 Hz, 2 **H****_Ar_**), 7.39 (t, 2H, *J* = 7.5 Hz, 2 **H****_Ar_**), 7.67 (d, 2H, *J* = 7.2 Hz, 2 **H****_Ar_**), 7.87 (d, 2H, *J* = 7.2 Hz, 2 **H****_Ar_**), 8.34 (s, 1H, N**H**).

#### 3.2.4. (9H-fluoren-9-yl)Methyl 2-(2-(tert-butoxy)-2-oxoethyl)Hydrazine-1-Carboxylate (**5**)

Compound **5** was synthesized according to the procedure previously reported by Spiegel et al. [28], reproducing the experimental protocol without modification.

Briefly, to a stirred solution of compound **4** (1 g, 3.93 mmol, 1.0 equiv.) and K_2_CO_3_ (597.8 mg, 4.32 mmol, 1.1 equiv.) in anhydrous DMF (16 mL), *tert*-butyl bromoacetate (0.58 mL, 3.93 mmol, 1.1 equiv.) was added at 0 °C. The reaction mixture was stirred at 0 °C for 5 min, then allowed to warm to room temperature and stirred for 20 h. The mixture was diluted with water (250 mL) and extracted with EtOAc (3 × 200 mL). The combined organic layers were washed with brine (250 mL), dried over MgSO_4_, filtered, and concentrated under reduced pressure. Purification by silica gel column chromatography using hexane/EtOAc (70/30, *v*/*v*) afforded product **5** as a colorless oil (1.77 g, 60%). Spectroscopic data (^1^H NMR) were in perfect agreement with literature values [28].

Analytical HPLC (0–100% B in 30 min, 0.8 mL·min^−1^), Rt = 21.22 min, purity: 90% at 214 nm.

^1^H NMR (400 MHz, CDCl_3_) *δ* 1.47 (s, 9H, 3 C**H_3_**, *t*Bu), 3.41 (s, 2H, N-C**H_2_**-CO_2_*t*Bu, additional arm), 4.25 (dd, 1H, *J* = 13.5, 6.4 Hz, C**H**), 4.31 (d, 2H, *J* = 7.0 Hz, C**H_2_**), 7.32 (t, 2H, *J* = 7.4 Hz, 2 **H****_Ar_**), 7.40 (t, 2H, *J* = 7.4 Hz, 2 **H****_Ar_**), 7.59 (d, 2H, *J* = 7.3 Hz, 2 **H****_Ar_**), 7.77 (d, 2H, *J* = 7.5 Hz, 2 **H****_Ar_**).

#### 3.2.5. (9H-fluoren-9-yl)Methyl 2-(4-bromobutanoyl)-2-(2-(tert-butoxy)-2-oxoethyl)Hydrazine-1-Carboxylate (**6**)

To a stirred solution of **5** (1 g, 2.92 mmol, 1 equiv.) and 2,6-lutidine (0.68 mL, 5.85 mmol, 2 equiv.) in anhydrous DCM (11 mL), a solution of 4-bromobutyryl chloride (0.338 mL, 2.92 mmol, 1 equiv.) in DCM (14 mL) was added dropwise. The reaction mixture was stirred at room temperature for 30 min, then washed with H_2_O and 0.1% *v*/*v* TFA (3 × 50 mL). The aqueous phase was extracted with DCM (2 × 50 mL), and the combined organic layers were dried over MgSO_4_, filtered, and concentrated under reduced pressure. Purification by silica gel column chromatography hexane/AcOEt (70/30, *v*/*v*) afforded the product **6** as white powder (1.11 g, 74%).

Analytical HPLC (0–100% B in 30 min, 0.8 mL·min^−1^), Rt = 22.05 min, purity: 95% at 214 nm.

^1^H NMR (400 MHz, CDCl_3_) *δ* 1.46 (s, 9H, 3 **C**H_3_, *t*Bu), 2.12 (s, 2H, CH_2_-C**H_2_**-CH_2_-Br), 2.51–2.42 (m, 2H, C**H_2_**-CH_2_-CH_2_-Br), 3.43 (s, 2H, C**H_2_**-Br), 4.23 (t, 1H, *J* = 6.1 Hz, C**H**, Fmoc), 4.60 (s, 2H, C**H_2_**, Fmoc), 7.33 (td, 2H, *J* = 7.5, 1.2 Hz, 2 **H****_Ar_**), 7.45–7.39 (m, 2H, 2 **H****_Ar_**), 7.56 (d, 2H, *J* = 7.5 Hz, 2 **H****_Ar_**), 7.77 (dt, 2H, *J* = 7.6, 1.0 Hz, 2 **H****_Ar_**).

^13^C NMR (100.6 MHz, CDCl_3_) *δ* 27.99 (CH_2_-**C**H_2_-CH_2_-Br), 28.53 (3 **C**H_3_, *t*Bu), 30.38 (**C**H_2_-CH_2_-CH_2_-Br), 33.69 (**C**H_2_-Br), 47.58 (**C**H, Fmoc), 49.18 (NH-**C**H_2_-CO_2_*t*Bu, additional arm), 67.96 (O-**C**H_2_, Fmoc), 83.33 (**C**, *t*Bu), 120.62 (2 **C**H_Ar_), 125.24 (2 **C**H_Ar_), 127.62 (2 **C**H_Ar_), 128.42 (2 **C**H_Ar_), 141.91 (2 **C**_Ar_), 143.63 (2 **C**_Ar_), 155.24 (CH-**C**O_2_-N), 167.21 (NH-**C**O-CH_2_, N-CO).

HPLC-MS (ESI^+^) *m*/*z* calcd for C_25_H_29_BrN_2_O_5_ [M + H]^+^: 517.13; found: 517.00.

#### 3.2.6. Di-Tert-Butyl 2,2′-(7-(4-(1-(2-(tert-butoxy)-2-oxoethyl)hydrazineyl)-4-oxobutyl)-1,4,7-triazonane-1,4-diyl)Diacetate (**7**)

To a stirred solution of **6** (120 mg, 0.237 mmol, 1 equiv.) in CH_3_CN (10 mL), a solution of NO2A-*t*Bu (76 mg, 0.213 mmol, 0.9 equiv.) and DIPEA (0.082 mL, 0.474 mmol, 2 equiv.) in CH_3_CN (5 mL) was added dropwise at 0 °C. The reaction mixture was allowed to reach room temperature and stirred for 20 h. The solvent was removed under reduced pressure, and the crude residue was purified by preparative HPLC using a gradient from 50% to 70% B over 25 min (Rt = 13.2 min). The appropriate fraction was concentrated under reduced pressure and lyophilized to afford compound **7** as a yellow oil (67 mg, 55%).

Analytical HPLC (50–70% B in 20 min, 0.8 mL·min^−1^), Rt = 14.35 min, purity: 95% at 214 nm.

^1^H NMR (400 MHz, D_2_O) *δ* 1.48 (s, 27H, 9 C**H_3_**, *t*Bu), 2.10–2.07 (m, 2H, N-CO-CH_2_-C**H_2_**, macrocycle linker), 2.84 (dt, 4H, *J* = 14.6, 7.0 Hz, N-CO-C**H_2_**-CH_2_-C**H_2_**, macrocycle linker), 3.04–3.33 (m, 12H, 3 N-C**H_2_**_-_C**H_2_**, macrocycle core), 3.58 (d, 4H, *J* = 7.6 Hz, 2 N-C**H_2_**-CO_2_*t*Bu, macrocycle core), 4.26 (s, 2H, N-C**H_2_**-CO_2_*t*Bu, additional arm).

^13^C NMR (100.6 MHz, D_2_O) *δ* 19.33 (N-CO-CH_2_-**C**H_2_, macrocycle linker), 26.82 (9 **C**H_3_, *t*Bu), 28.64 (N-CO-**C**H_2,_ macrocycle linker) 49.16 (2 N-**C**H_2_, macrocycle core), 50.75 (N-**C**H_2_-CO_2_*t*Bu, additional arm), 52.68 (2 N-**C**H_2_, macrocycle core), 55.93 (2 N-**C**H_2_-CO_2_*t*Bu, macrocycle core), 83.16 (3 **C**, *t*Bu), 172.32 (3 **C**O_2_*t*Bu), 176.3 (N-**C**O).

HPLC-MS (ESI^+^) *m*/*z* calcd for C_28_H_53_N_5_O_7_ [M + H]^+^: 572.40; found: 572.35.

#### 3.2.7. Di-Tert-Butyl 2,2′-(7-(4-(1-(2-(tert-butoxy)-2-oxoethyl)-2-(6-(2,5-dioxo-2,5-dihydro-1H-pyrrol-1-yl)hexanoyl)hydrazineyl)-4-oxobutyl)-1,4,7-triazonane-1,4-diyl)Diacetate (**8**)

To a stirred solution of **7** (16 mg, 0.028 mmol, 1 equiv.) and NMM (0.012 mL, 0.111 mmol, 4 equiv.) in anhydrous DCM (0.312 mL), a solution of 6-maleimidohexanoic acid (5.9 mg, 0.028 mmol, 1 equiv.) in anhydrous DCM (0.16 mL) was added at 0 °C. A solution of HATU (10.6 mg, 0.028 mmol, 1 equiv.) in a minimal volume of DMF (0.25 mL) was then added. The reaction mixture was allowed to warm slowly to room temperature and stirred for 15 h. The mixture was washed with H_2_O and 0.1% *v*/*v* TFA (3 × 1 mL), and the aqueous phase was extracted with DCM (2 × 1 mL). The combined organic layers were dried over MgSO_4_, filtered, and concentrated under reduced pressure. The crude product was purified by preparative HPLC using 50 to 70% B over 25 min (Rt = 18.5 min) to yield compound **8** as a yellow oil (17 mg, 80%).

Analytical HPLC (50–70% B in 30 min, 0.8 mL·min^−1^, Rt = 14.35 min, purity: 95% at 214 nm.

^1^H NMR (400 MHz, CDCl_3_) *δ* 1.32–1.28 (m, 2H, N-CH_2_-CH_2_-C**H_2_**, maleimide linker), 1.46 (s, 27H, 9 C**H_3_**, *t*Bu), 1.69–1.60 (m, 4H, N-CH_2_-C**H_2_**-CH_2_-C**H_2_**, maleimide linker), 2.38–2.20 (m, 4H, C**H_2_**-CO-NH, maleimide linker, N-CO-CH_2_-C**H_2_**, macrocycle linker), 3.12–3.95 (m, 24H, 3 N-C**H_2_**-C**H_2_**, macrocycle core, 3 N-C**H_2_**-CO_2_*t*Bu, CO-C**H_2_**-CH_2_-C**H_2_**-N_,_ macrocycle linker, N-C**H_2_**-CH_2_-CH_2_-CH_2_CO-N, maleimide linker), 6.73 (s, 2H, **H**C=C**H**, maleimide core).

^13^C NMR (100.6 MHz, CDCl_3_) *δ* 18.94 (N-CO-CH_2_-**C**H_2_, macrocycle linker), 25.10 (**C**H_2_-CH_2_-CO-N, maleimide linker), 26.73 (9 **C**H_3_, *t*Bu), 28.6 (**C**H_2_-CH_2_-CH_2_-CO-N, maleimide linker), 29.48 (N-CH_2_-**C**H_2_, maleimide linker), 30.38 (N-CO-**C**H_2,_ macrocycle linker), 34.14 (**C**H_2_-CO-N, maleimide linker), 38.26 (N-**C**H_2_-CH_2_, maleimide linker), 49.41 (N-**C**H_2_-CO_2_*t*Bu, additional arm), 50.92 (2 N-**C**H_2_, macrocycle core), 52.56 (2 N-**C**H_2_, macrocycle core), 52.93 (**C**H_2_-N, macrocycle linker), 55.05 (2 N-**C**H_2_, macrocycle core), 57.95 (2 N-**C**H_2_-CO_2_*t*Bu, macrocycle core), 82.82 (3 **C**, *t*Bu), 134.72 (H**C**=**C**H, maleimide core), 168.57 (NH-N-**C**O), 171.33 (3 **C**O_2_*t*Bu), 173.13 (2 **C**O), 174.49 (2 **C**O-NH).

HPLC-MS (ESI^+^) *m*/*z* calcd for C_38_H_64_N_6_O_10_ [M + H]^+^: 765.47; found: 765.46.

#### 3.2.8. 2,2′-(7-(4-(1-(carboxymethyl)-2-(6-(2,5-dioxo-2,5-dihydro-1H-pyrrol-1-yl)hexanoyl)hydrazineyl)-4-oxobutyl)-1,4,7-triazonane-1,4-diyl)Diacetic Acid (**NO2A-2C-AHM)**

To a solution of compound **8** (17 mg, 0.022 mmol, 1 equiv.) in DCM (1 mL) was added TFA (0.441 mL, 5.72 mmol, 260 equiv.) to remove the *tert*-butyl protecting groups. The reaction mixture was stirred at room temperature for 9 h, after which TFA was removed under reduced pressure and co-evaporated with DCM (4 × 1.5 mL). The resulting residue was precipitated in Et_2_O (2 mL, −20 °C). The suspension was centrifuged for 25 min at 5000 rpm and the supernatant decanted and removed. **NO2A-2C-AHM** was obtained as a white powder in quantitative yield (13.2 mg).

Analytical HPLC (5–50% B in 15 min, 0.8 mL·min^−1^), Rt = 11.75 min, purity: 97% at 214 nm.

^1^H NMR (400 MHz, D_2_O) *δ* 1.39–1.25 (m, 2H, N-CH_2_-CH_2_-C**H_2_**, maleimide linker), 1.73–1.54 (m, 4H, N-CH_2_-C**H_2_**-CH_2_-C**H_2_**, maleimide linker), 2.07 (m, 2H, CO-CH_2_-C**H_2_**-CH_2_-N, macrocycle linker), 2.34 (t, 2H, *J* = 7.2 Hz, C**H_2_**-CO-NH-N, maleimide linker), 2.62 (s, 2H, CO-CH_2_-CH_2_-C**H_2_**-N, macrocycle linker), 3.28 (s, 4H, N-C**H_2_**-CO_2_H, additional arm, CO-C**H_2_**-CH_2_-CH_2_-N, macrocycle linker), 3.69–3.35 (m, 14H, 3 N-C**H_2_**-C**H_2_**, macrocycle core, N-C**H_2_**-CH_2_, maleimide linker), 3.80 (s, 4H, 2 N-C**H_2_**-CO_2_H, macrocycle core), 6.84 (s, 2H, **H**C=C**H**, maleimide core).

^13^C NMR (100.6 MHz, D_2_O) *δ* 19.56 (N-CO-CH_2_-**C**H_2_, macrocycle linker), 25.02 (**C**H_2_-CH_2_-CO-N, maleimide linker), 26.21 (**C**H_2_-CH_2_-CH_2_-CO-N, maleimide linker), 28.03 (N-CH_2_-**C**H_2_, maleimide linker), 29.78 (N-CO-**C**H_2_, macrocycle linker), 33.78 (**C**H_2_-CO-N, maleimide linker), 38.13 (N-**C**H_2_-CH_2_, maleimide linker), 50.20 (2 N-**C**H_2_, macrocycle core), 51.53 (2 N-**C**H_2_, macrocycle core), 51.68 (N-**C**H_2_-CO_2_H, additional arm), 54.62 (2 N-**C**H_2_, macrocycle core), 57.77 (**C**H_2_-N, macrocycle linker), 58.06 (2 N-**C**H_2_-CO_2_, macrocycle core), 135.04 (H**C**=**C**H, maleimide core), 172.38 (NH-N-**C**O), 173.69 (2 **C**O), 176.56 (**C**O-NH) 177.08 (3 **C**OOH).

HPLC-MS (ESI^+^) *m*/*z* calcd for C_26_H_41_N_6_O_10_ [M + H]^+^: 597.28; found: 597.35.

#### 3.2.9. 2,2′-(7-(1-carboxy-4-(2-(6-(2,5-dioxo-2,5-dihydro-1H-pyrrol-1-yl)hexanoyl)hydrazineyl)-4-oxobutyl)-1,4,7-triazonane-1,4-diyl)Diacetic Acid (**NODAGA-HM**)

Compound **9** was synthesized following the procedure previously described by our group [21]. The modification that Boc deprotection was executed using neat TFA (100%) instead of a TFA/DCM mixture. The reaction went to completion at 20 min at room temperature (compared to the original 5 h protocol) without affecting the isolated yield.

To a solution of compound **9** (33.31 mg, 0.148 mmol, 1.0 equiv.) and DIPEA (77.29 µL, 0.444 mmol, 3.0 equiv.) in DMF (3.33 mL), a solution of NODAGA-NHS ester (130 mg, 0.177 mmol, 1.2 equiv.) in DMF (3.33 mL) was added dropwise at 0 °C. The reaction mixture was slowly allowed to warm to room temperature and stirred for 15 h. The solvent was then removed under reduced pressure, and the crude product was purified by preparative HPLC using a linear gradient from 0% to 70% B over 20 min (Rt = 9.29 min). The product-containing fraction was concentrated and lyophilized to afford **NODAGA-HM** as a white powder (44 mg, 50%).

Analytical HPLC (0–100% B in 20 min, 0.8 mL·min^−1^), Rt = 10.77 min, purity: 95% at 214 nm.

^1^H NMR (400 MHz, D_2_O) *δ* 1.37–1.23 (m, 2H, N-CH_2_-CH_2_-C**H_2_**, maleimide linker), 1.70–1.52 (m, 4H, N-CH_2_-C**H_2_**-CH_2_-C**H_2_**, maleimide linker), 2.24–2.00 (m, 2H, NH-CO-CH_2_-C**H_2_**, macrocycle linker), 2.31 (t, 2H, *J* = 7.4 Hz, C**H_2_**-CO-NH, maleimide linker), 2.54 (td, 2H, *J* = 7.5, 2.3 Hz, NH-CO-C**H_2_**_,_ macrocycle linker), 3.33–3.01 (m, 12H, 3 N-C**H_2_**-C**H_2_**, macrocycle core), 3.51 (t, 2H, *J* = 7.0 Hz, N-C**H_2_**-CH_2_, maleimide linker), 3.71–3.62 (m, 1H, CH_2_-C**H**-COOH, macrocycle linker), 3.89 (s, 4H, 2 N-C**H_2_**-COOH, macrocycle core), 6.85 (s, 2H, **H**C=C**H**, maleimide core).

^3^C NMR (100.6 MHz, D_2_O) *δ* 24.01 (N-CO-CH_2_-**C**H_2_, macrocycle linker), 24.48 (**C**H_2_-CH_2_-CO-N, maleimide linker), 25.42 (**C**H_2_-CH_2_-CH_2_-CO-N, maleimide linker), 27.34 (N-CH_2_-**C**H_2_, maleimide linker), 30.25 (NH-CO-**C**H_2_-CH_2_-CH-COOH, macrocycle linker), 33.27 (**C**H_2_-CO-NH, maleimide linker), 37.50 (N-**C**H_2_-CH_2_, maleimide linker), 45.84 (2 N-**C**H_2_, macrocycle core), 49.23 (2 N-**C**H_2_, macrocycle core), 50.91 (2 N-**C**H_2_, macrocycle core), 55.80 (2 N-**C**H_2_-CO_2_H, macrocycle core), 63.74 (**C**H-CO_2_H, macrocycle linker), 134.35 (H**C**=**C**H), 172.56 (2 **C**O, maleimide core), 173.50 (**C**O-NH), 174.42 (NH-**C**O), 175.56 (CH-**C**OOH), 175.90 (2 **C**OOH).

HPLC-MS (ESI^+^) *m*/*z* calcd for C_25_H_38_N_6_O_10_ [M + H]^+^: 583.27; found: 583.05.

### 3.3. Complexation

#### 3.3.1. General Procedure for the Synthesis of AlF-BFC Complexes

A solution of AlCl_3_ (1.5 equiv.) in 0.1 M sodium acetate buffer (pH 4.5) was mixed with a solution of NaF (4.0 equiv.) prepared in the same buffer and stirred for approximately 10 min at room temperature to generate the {AlF}^2+^ species. A solution of the respective BFC (1.0 equiv.) in 0.1 M sodium acetate buffer (pH 4.5) was subsequently added, followed by absolute ethanol to achieve a final EtOH content of 50% *v*/*v* in the reaction mixture. The mixture was heated at 90 °C for 30 min. The resulting Al^nat^F-BFC complex was purified by preparative HPLC using a linear gradient from 5% to 50% B over 15 min and lyophilized.

- **AlF-NO2A-2C-AHM**

White powder. Yield: 90%.

Analytical HPLC (5–50% B in 20 min, 0.8 mL·min^−1^), Rt = 12,46 min, purity: 95% at 214 nm.

^1^H NMR (400 MHz, D_2_O) *δ* 1.38–1.24 (m, 2H, N-CH_2_-CH_2_-C**H_2_**, maleimide linker), 1.61 (dd, 4H, *J* = 15.1, 7.5 Hz, N-CH_2_-C**H_2_**-CH_2_-C**H_2_**, maleimide linker), 1.96–1.82 (m, 2H, CO-CH_2_-C**H_2_**-CH_2_-N, macrocycle linker), 2.32 (t, 2H, *J* = 7.3 Hz, C**H_2_**-CO-NH, maleimide linker), 3.82–2.79 (m, 22H, 3 N-C**H_2_**-C**H_2_**, macrocycle core, N-C**H_2_**-CH_2_, maleimide linker, N-C**H_2_**-CO_2_H_,_ additional arm_,_ C**H_2_**-N, macrocycle linker, 2 N-C**H_2_**-CO_2_H, C**H_2_**-CO-N, maleimide linker), 6.82 (d, 2H, *J* = 3.8 Hz, **H**C=C**H**, maleimide core).

^19^F NMR (376 MHz, D_2_O) *δ* −166.06.

HPLC-MS (ESI^+^) *m*/*z* calcd for C_25_H_38_AlFN_6_O_10_ [M + H]^+^: 641.27; found: 641.20.

- **AlF-NODAGA-HM**

White powder. Yield: 80%.

Analytical HPLC (5–50% B in 20 min, 0.8 mL·min^−1^), Rt = 12.29 min, purity: 95% at 214 nm.

^1^H NMR (400 MHz, D_2_O) *δ* 1.38–1.25 (m, 2H, N-CH_2_-CH_2_-C**H_2_**, maleimide linker), 1.71–1.50 (m, 4H, N-CH_2_-C**H_2_**-CH_2_-C**H_2_**, maleimide linker), 2.13–2.02 (m, 1H, NH-CO-CH_2_-C-H*, macrocycle linker), 2.29 (t, 2H, *J* = 7.4 Hz, C**H_2_**-CO-NH, maleimide), 2.55 (m, 1H, NH-CO-CH_2_-C-H*,macrocycle linker), 3.86–2.67 (m, 19H, 3 N-C**H_2_**-C**H_2_**, macrocycle core, N-C**H_2_**-CH_2_, maleimide linker, CH_2_-C**H**-COOH, macrocycle linker, 2 N-C**H_2_**-COOH, macrocycle core), 6.84 (s, 2H, **H**C=C**H**, maleimide core).

^19^F NMR (376 MHz, D_2_O) *δ* −166.97, −168.77.

HPLC-MS (ESI^+^) *m*/*z* calcd for C_25_H_36_AlFN_6_O_10_ [M + H]^+^: 627.22; found: 627.20.

#### 3.3.2. General Procedure for the Synthesis of ^nat^Lu-BFC Complexes

A solution of the BFC (1.0 equiv.) in 0.1 M sodium acetate buffer (pH 4.5) was treated with a solution of Lu(NO_3_)_3_ (1.5 equiv.) prepared in the same buffer at room temperature. The resulting mixture was heated at 40 °C for 30 min. Purification by HPLC was carried out using a linear gradient from 5% to 50% B over 15 min, followed by lyophilization.

- **Lu-NODAGA-HM**

White powder. Yield: 70%.

Analytical HPLC (5–50% B in 20 min, 0.8 mL·min^−1^), Rt = 12,13 min, purity: 95% at 214 nm.

^1^H NMR (400 MHz, D_2_O) *δ* 1.23–1.37 (m, 2H, N-CH_2_-CH_2_-C**H_2_**, maleimide linker), 1.52–1.70 (m, 4H, N-CH_2_-C**H_2_**-CH_2_-C**H_2_**, maleimide linker), 2.03–2.12 (m, 1H, NH-CO-CH_2_-C-H*, macrocycle linker), 2.28 (t, 2H, *J* = 7.4 Hz, 2H, C**H_2_**-CO-NH, maleimide linker), 2.63 (m, 1H, NH-CO-CH_2_-C-H*, macrocycle linker), 2.84–3.25 (m, 12H, 3 N-C**H_2_**-C**H_2_**, macrocycle core), 3.47–3.85 (m, 7H, N-C**H_2_**-CH_2_, maleimide linker, CH_2_-C**H**-COOH, macrocycle linker, 2 N-C**H_2_**-COOH, macrocycle core), 6.81 (s, 2H, **H**C=C**H**, maleimide core).

HPLC-MS (ESI^+^) *m*/*z* calcd for C_25_H_36_LuN_6_O_10_ [M + H]^+^: 755.18; found: 755.15.

### 3.4. Radiolabeling

#### 3.4.1. Radiosynthesis of [^18^F]AlF-BFC Complexes

[^18^F]NaF was prepared on an AllInOne^®^ synthesis module according to the procedure reported by Collet et al. [37]. Briefly, [^18^F]NaF was recovered from a QMA chloride cartridge using 3 mL of 0.9% *w*/*v* NaCl solution, affording a decay-corrected radiochemical yield (RCY) of 80 ± 3% (n = 10).

The volume of AlCl_3_ corresponding to 0.4–1.0 equivalents relative to the bifunctional chelator (BFC) was added from an aluminum chloride solution (2 mM in 0.9% NaCl). The corresponding AlCl_3_ solution was combined with 500 μL of [^18^F]NaF (50 MBq) in 1250 μL of 0.1 M sodium acetate buffer (pH 4.5) prepared in EtOH/H_2_O (50–80%, *v*/*v*). The mixture was stirred at room temperature for 5 min. A solution of the corresponding BFC in Milli-Q water (1 mg·mL^−1^, 30 µL, 1.0 equiv.) was then added, and radiolabeling was carried out at 90 °C for 15 min under continuous agitation (300 rpm) using a Thermomixer.

Radiochemical conversion (RCC) was determined by radio-UHPLC using a linear gradient from 5% to 40% mobile phase B over 15 min at a flow rate of 0.6 mL·min^−1^. Under these conditions, **[^18^F]AlF-NO2A-2C-AHM** and **[^18^F]AlF-NODAGA-HM** eluted at Rt of 2.57 and 2.13 min, respectively. Additional analysis was performed by radio-TLC on silica gel plates using H_2_O/CH_3_CN (4/6, *v*/*v*) as the mobile phase, yielding Rf values of 0.63 and 0.61, respectively.

Stability studies were performed directly on the crude radiolabeling reaction mixture. After completion of the radiolabeling reaction, aliquots were maintained at room temperature and analyzed every hour for 5 h by radio-UHPLC to determine the percentage of intact radiolabeled compound. Radiochemical stability was assessed by monitoring the evolution of radiochemical purity over time.

#### 3.4.2. Radiosynthesis of [^177^Lu]Lu-BFC Complexes

Radiolabeling reactions were carried out in five distinct buffer systems to evaluate the influence of the reaction media on the overall radiolabeling efficiency: **Buffer 1**: 0.7 M NaOAc, pH 8.0; **Buffer 2**: 0.7 M NaOAc, pH 4.5; **Buffer 3**: 0.03 M NaOAc, pH 4.5, with 40% *v*/*v* EtOH; **Buffer 4**: 0.7 M NaOAc, pH 4.5 and 0.56 M sodium ascorbate; **Buffer 5**: 1.5 M NaOAc, pH 6.0, with 0.04 M ascorbic acid, AcOH, and EtOH.

A solution of [^177^Lu]LuCl_3_ (100–380 MBq in 0.1 M HCl, 0.4 mL) was added to the BFC solution (1 mg·mL^−1^ in Milli-Q water), together with the appropriate buffer. The reaction mixture was stirred at the selected temperature (40 °C, 60 °C, or 90 °C) for 30 min under agitation (300 rpm) using a Thermomixer. RCC was evaluated by radio-HPLC using a linear gradient from 5% to 50% mobile phase B over 15 min at a flow rate of 0.6 mL·min^−1^. Under these conditions, **[^177^Lu]Lu-NODAGA-HM** eluted at a Rt of 5.32 min.

### 3.5. Molecular Modeling

DFT calculations were performed using the Gaussian16 revision B.01-SMP software package [38]. Geometry optimizations of the AlF complexes were carried out using the B3LYP functional with the 6-31G(d) basis set, whereas Lu-containing complexes were optimized at the B3LYP/def2-TZVP level, incorporating the Stuttgart/Dresden (SDD) effective core potential for lutetium. All geometry optimizations were performed in aqueous solution using the SMD implicit solvation model with water as the solvent.

Default Gaussian16 convergence criteria were used for both the self-consistent field procedure and the geometry optimizations. Solvent effects were included using the SMD implicit solvation model with water as the solvent.

To evaluate the intrinsic affinity of each ligand toward the Lu^3+^ ion, interaction energies (E_int_) were calculated for the optimized complexes in both the gas phase and aqueous solution according to the following Equation (1):

(1) E_int_ = E_complex_ − E_Lu_ − E_ligand_

where E_Lu_ and E_ligand_ correspond to the energies of the isolated Lu^3+^ ion and the free ligand, respectively, calculated in the geometry adopted within the optimized complex without further geometry optimization of the isolated fragments.

## 4. Conclusions

In this study, two new hybrid NOTA-based bifunctional chelators, **NO2A-2C-AHM** and **NODAGA-HM**, were designed, synthesized, and evaluated for their ability to complex the dual Al-^18^F/^177^Lu theranostic tandem. This synthetic study highlighted the critical importance of the BFC architecture—particularly the position of the additional chelating arm—on metal coordination, which was thoroughly confirmed by computational modeling, non-radioactive complexation, and radiolabeling studies. While the synthesis of **NO2A-2C-AHM** required the development of an optimized multistep route to overcome undesired side reactions, **NODAGA-HM** was obtained more directly through coupling with a commercially available NODAGA-NHS precursor.

Non-radioactive complexation studies showed that both BFCs successfully formed stable AlF complexes, indicating that the structural modifications introduced into the NOTA scaffold did not compromise {AlF}^2+^ coordination. This was further confirmed by efficient Al^18^F radiolabeling and good stability. In contrast, markedly different behaviors were observed for lutetium complexation. While **NO2A-2C-AHM** failed to complex Lu^3+^ under both non-radioactive and radioactive conditions, **NODAGA-HM** exhibited efficient complexation and high radiochemical conversion with ^177^Lu under optimized radiolabeling conditions.

Molecular modeling accurately predicted these experimental observations. Although both ligands were able to accommodate the AlF core in a distorted octahedral geometry, their behavior toward Lu^3+^ differed significantly. The positioning of the additional donor arm closer to the NOTA macrocycle provides a coordination environment better suited to the larger ionic radius of Lu^3+^ in the **NODAGA-HM** BFC, whereas the geometry of **NO2A-2C-AHM** is less suited to stabilizing lutetium complexes in aqueous medium. These findings demonstrate that donor-arm positioning is a key structural parameter governing the balance between Al^18^F and ^177^Lu complexation.

Overall, the combined experimental and computational results demonstrate that increasing the linker length alone, as implemented in **NO2A-2C-AHM**, does not improve Lu^3+^ coordination. Although this structural modification preserves efficient AlF complexation, it does not provide a coordination environment favorable for lutetium, whereas the more compact donor-atom arrangement in **NODAGA-HM** enables efficient Lu^3+^ complexation. These findings highlight that the spatial organization of the donor atoms, rather than linker extension alone, is the key structural parameter governing the balance between Al^18^F and ^177^Lu complexation. The **NODAGA-HM** BFC therefore represents a promising platform for the development of radiotheranostic agents compatible with both Al^18^F PET imaging and ^177^Lu targeted radionuclide therapy. Future work will focus on the conjugation of **NODAGA-HM** to tumor-targeting biomolecules. Owing to the presence of the maleimide functionality, **NODAGA-HM** can readily be conjugated to thiol-containing targeting biomolecules. In the field of radiopharmaceutical development, this challenge may be overcome by exploiting the large number of biomarkers that are overexpressed in tumor cells and can serve as relevant targets for imaging and therapy [39]. Following conjugation, optimization of the radiolabeling conditions will be necessary, as the attached biomolecule may influence the accessibility of the chelating cavity and the overall physicochemical properties of the resulting radiopharmaceutical. The stability of the resulting radiopharmaceuticals will be evaluated under physiologically relevant conditions, with serum stability, before comprehensive in vitro (e.g., cellular uptake, …) and in vivo (e.g., biodistribution, …) studies.

## Figures and Tables

**Table 1:** Performance of the BFC **3p-*****C*****-NETA** with various radiometals.

Radiometal	Radiolabeling Yield (%)	Stability ^a^	References
Al^18^F	62.3 ± 1.5		[17]
^67^Cu	96.1 ± 3.2		[18]
^68^Ga	97.7 ± 0.1		[17]
^177^Lu	99.4 ± 0.4		[19]
^86/90^Y	>99		[19]
^212/213^Bi	93.4 ± 1.9		[20]
^161^Tb	>90		[17]

^a^ Green = good stability; Orange = moderate stability.

## Data Availability

The original contributions presented in this study are included in the article/Supplementary Materials. Further inquiries can be directed to the corresponding authors.

## Supplementary Materials

The following supporting information can be downloaded at: https://www.mdpi.com/article/10.3390/ijms27167421/s1.

## Abbreviations

The following abbreviations are used in this manuscript:

BFC	Bifunctional chelator
PET	Positron emission tomography
RCC	Radiochemical conversion
RCY	Radiochemical yield
DTPA	Diethylenetriaminepentaacetic acid
NOTA	1,4,7-triazacyclononane-1,4,7-triacetic acid
DOTA	1,4,7,10-tetraazacyclododecane-1,4,7,10-tetraacetic acid
DOTAGA	2-[1,4,7,10-tétraazacyclododécane]-pentanedioic acid
SN_2_	Bimolecular nucleophilic substitution
DFT	Density Functional Theory
Rf	Retention factor
Rt	Retention time
