# Supplementary material for: Comparative Study of NOTA-Derived Chelators: Synthesis, Al18F/177Lu Radiolabeling and Computational Analysis
Source: Int J Mol Sci. 2026 Aug 19;27(16):7421. doi: 10.3390/ijms27167421 (PMC13513234; doi:10.3390/ijms27167421)
Supplement: Supplementary file 1 [file ijms-27-07421-s001.zip › ijms-4485648-supplementary.pdf]

Comparative Study of NOTA-Derived Chelators: Synthesis, $Al^{18}F/^{177}Lu$ Radiolabeling and Computational Analysis

Abir Dhimen [1,2], Xavier Assfeld [3], Gilles Karcher [2], Charlotte Collet [2,4,*] and Samir Acherar [1,*]

[1] Université de Lorraine, CNRS, LRGP, F-54000 Nancy, France

[2] Nancyclotep, Plateforme d'Imagerie Moléculaire, F-54511 Vandœuvre-lès-Nancy, France

[3] Université de Lorraine, CNRS, LPCT, F-54000 Nancy, France

[4] Université de Lorraine, INSERM, IADI, F-54000 Nancy, France

* Correspondence: charlotte.collet@univ-lorraine.fr (C.C.); samir.acherar@univ-lorraine.fr (S.A.)

Contents

1. NMR spectra and HPLC chromatograms of main products...2

1.1. Compound 2 (^{1}H and *J* mod ^{13}C NMR spectra)... 2

1.2. Compound 3 (^{1}H and *J* mod ^{13}C NMR spectra, and MS spectrum) 3

1.3. Compound 4 (^{1}H NMR spectrum) 4

1.4. Compound 5 (^{1}H NMR spectrum) 5

1.5. Compound 6 (^{1}H and ^{13}C-JMOD NMR spectra)... 6

1.6. Compound 7 (^{1}H and ^{13}C-JMOD NMR spectra)... 7

1.7. Compound 8 (^{1}H and ^{13}C-JMOD NMR spectra)... 8

1.8. NO2A-2C-AHM (^{1}H and ^{13}C-JMOD NMR spectra, and HPLC-MS analysis)........................ 9

1.9. NODAGA-HM (^{1}H and ^{13}C-JMOD NMR spectra, and HPLC-MS analysis)........................ 11

1.10. AlF-NO2A-2C-AHM (^{1}H and ^{19}F NMR spectra, and HPLC-MS analysis) 13

1.11. AlF-NODAGA-HM (^{1}H and ^{19}F NMR spectra, and HPLC-MS analysis) 15

1.12. Lu-NODAGA-HM (^{1}H NMR spectrum, and HPLC-MS analysis).. 17

2. Radiolabeling 19

2.1. [^{18}F]AlF-NODAGA-HM.. 19

2.2. [^{18}F]AlF-NO2A-2C-AHM... 20

2.3. [^{177}Lu]Lu-NODAGA-HM 21

3. Molecular Modeling Data 22

3.1. AlF-NO2A-2C-AHM and AlF-NODAGA-HM.. 22

3.2. Lu-NODAGA-HM 23

1. NMR spectra and HPLC chromatograms of main products

1.1. Compound 2 (^{1}H and ^{13}C-JMOD NMR spectra)

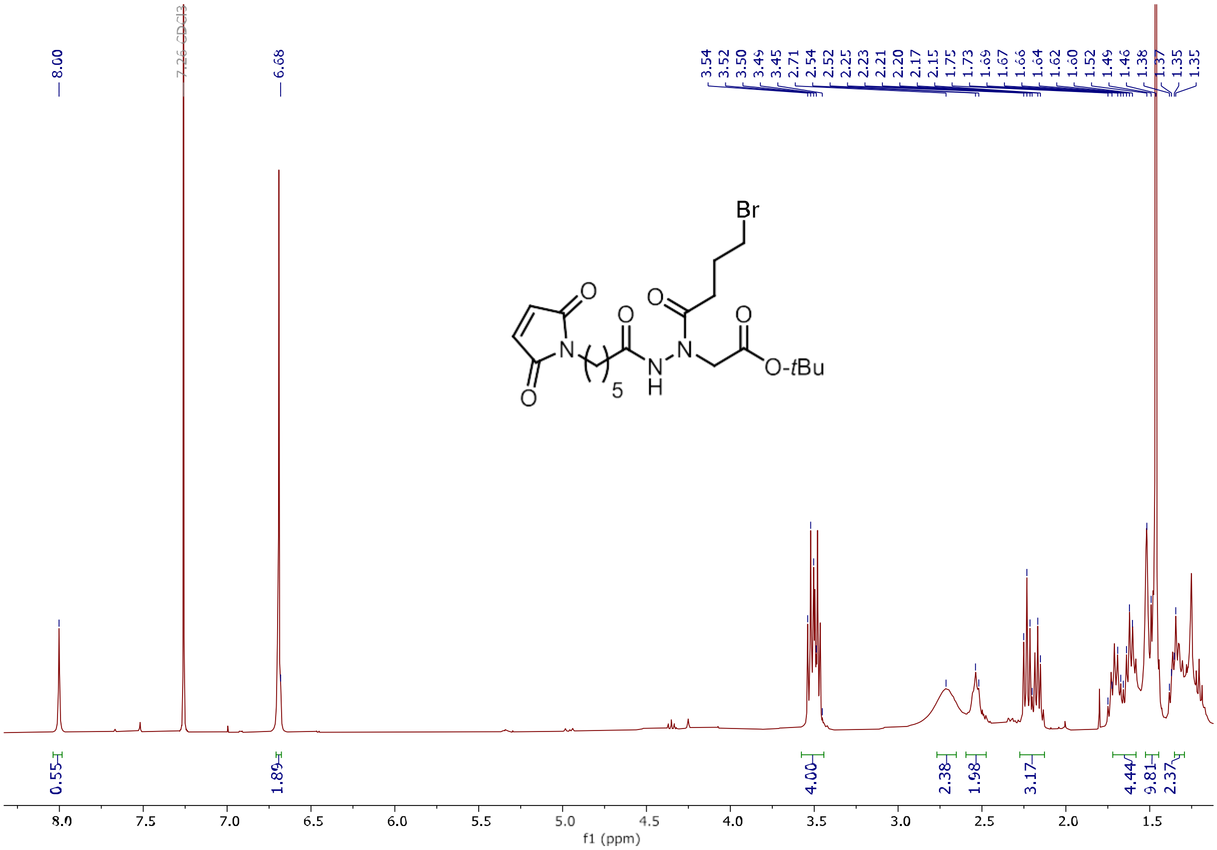

Figure S1: ^{1}H NMR (400 MHz) spectrum of compound 2 ($CDCl_3$, 298 K)

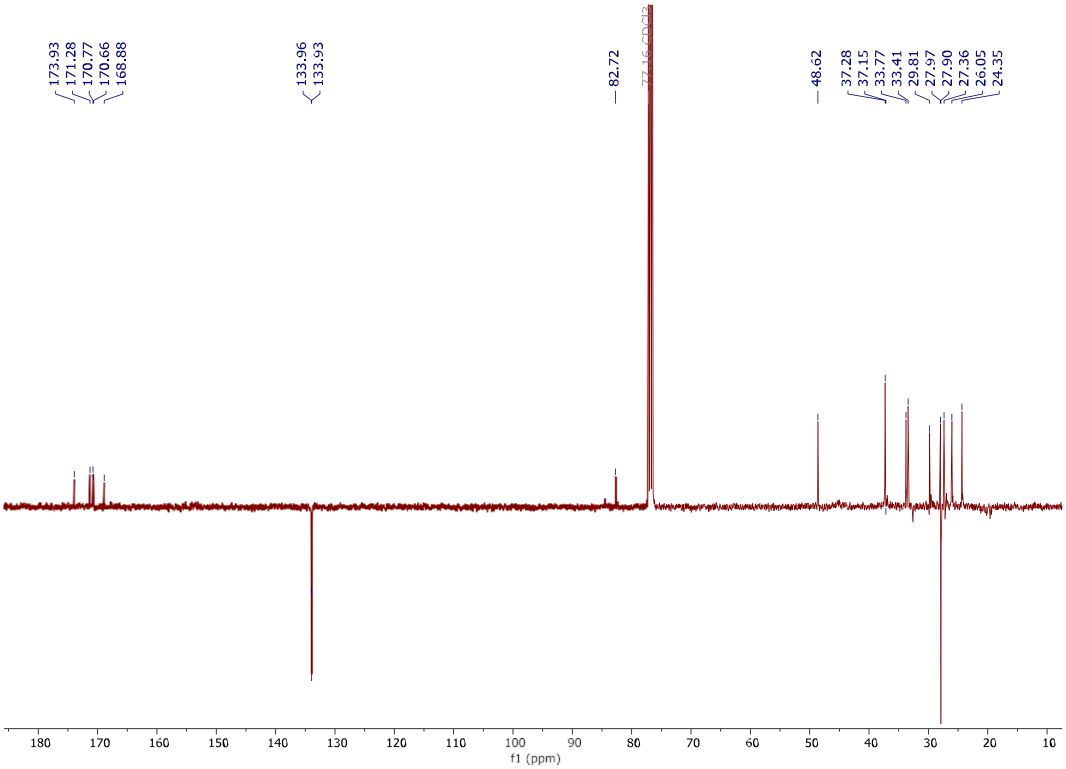

Figure S2: ^{13}C-JMOD NMR (100.6 MHz) spectrum of compound 2 ($CDCl_3$, 298 K)

1.2. Compound 3 (^{1}H and ^{13}C-JMOD NMR spectra, and MS spectrum)

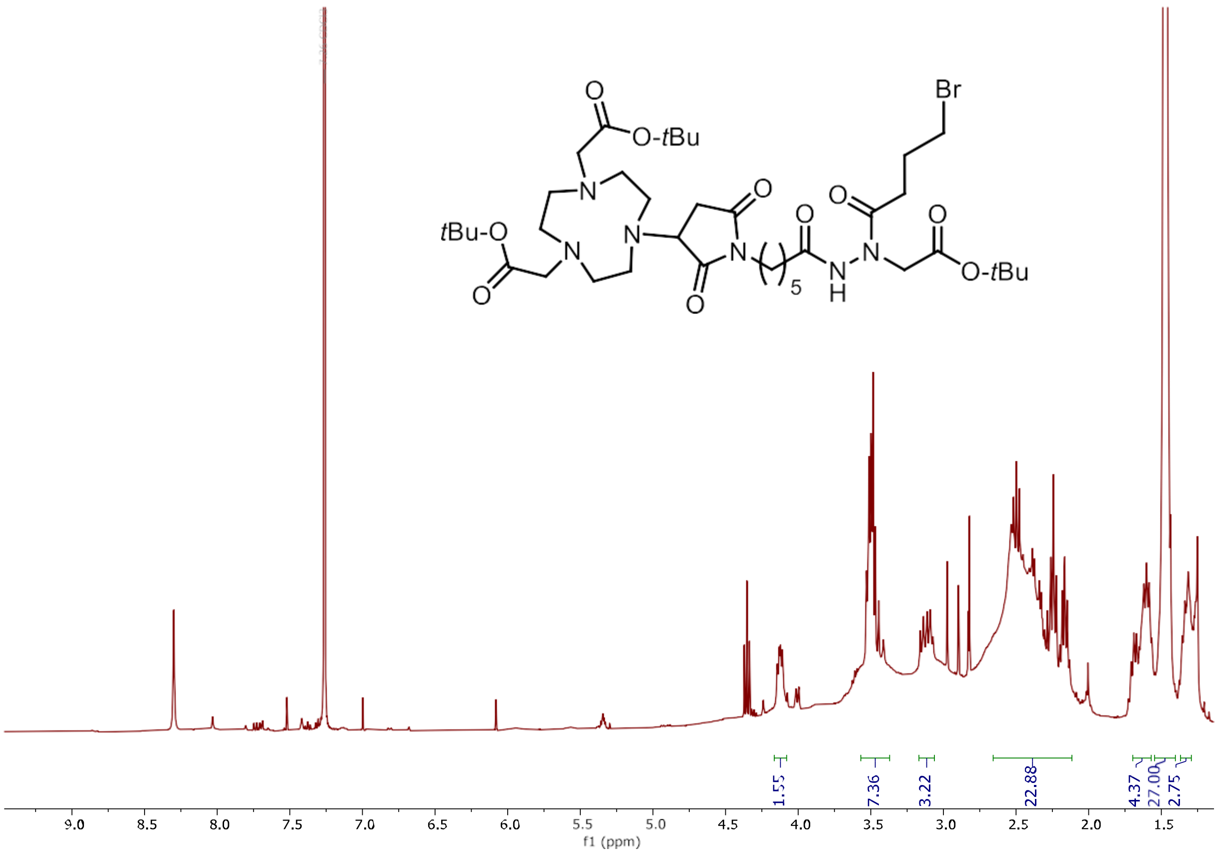

Figure S3: ^{1}H NMR (400 MHz) spectrum of compound 3 ($CDCl_3$, 298 K)

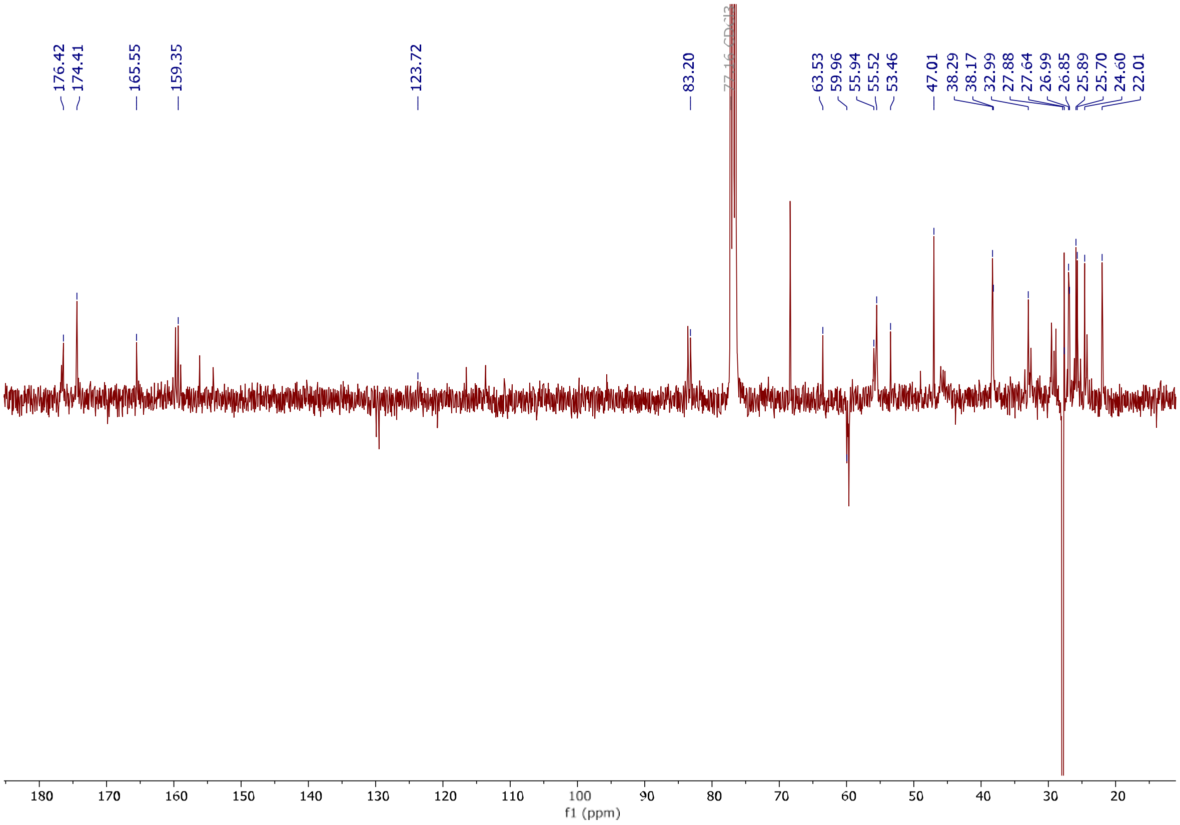

Figure S4: ^{13}C-JMOD NMR (100.6 MHz) spectrum of compound 3 ($CDCl_3$, 298 K)

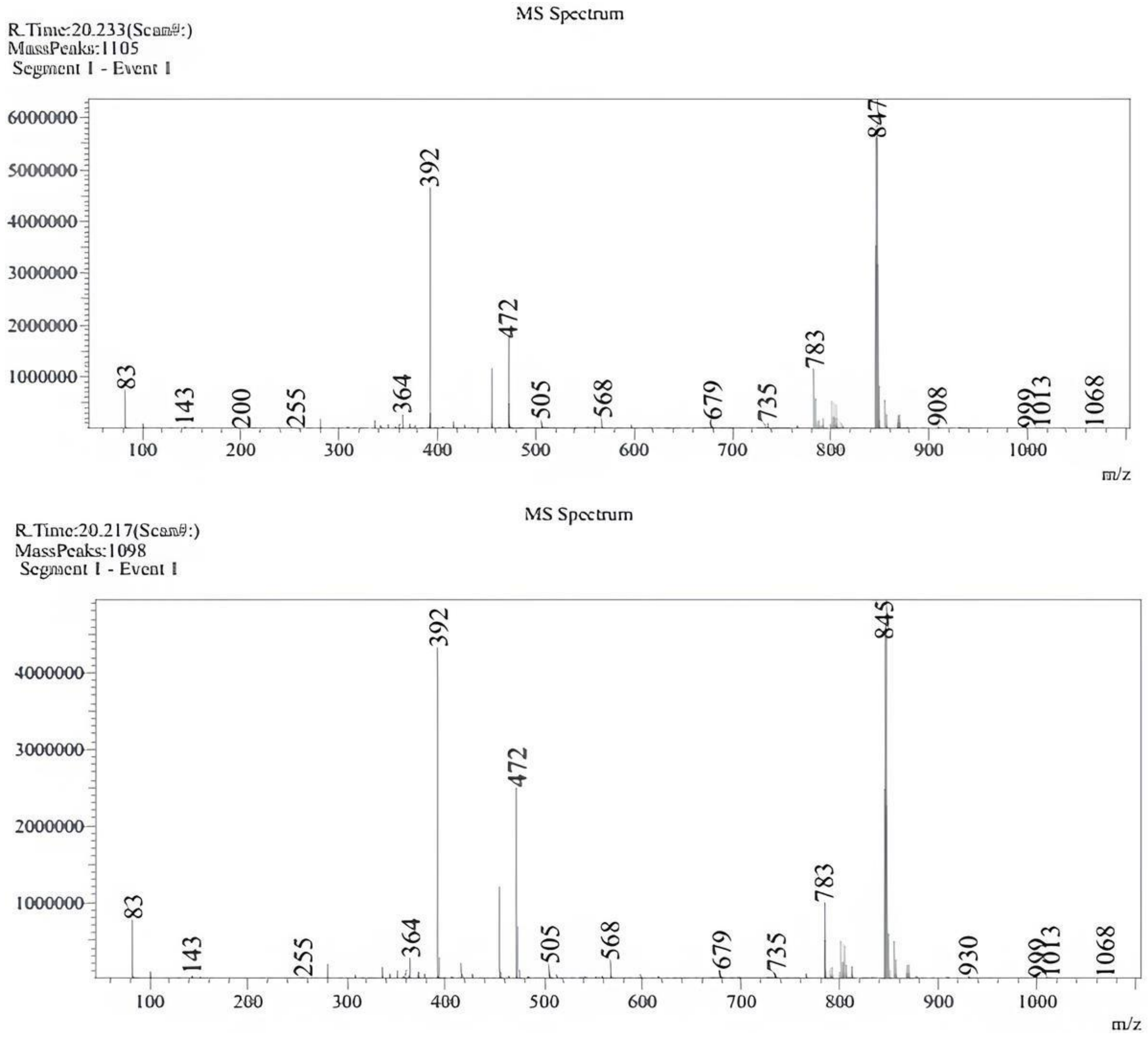

Figure S5: MS spectrum of compound 3

1.3. Compound 4 (^{1}H NMR spectrum)

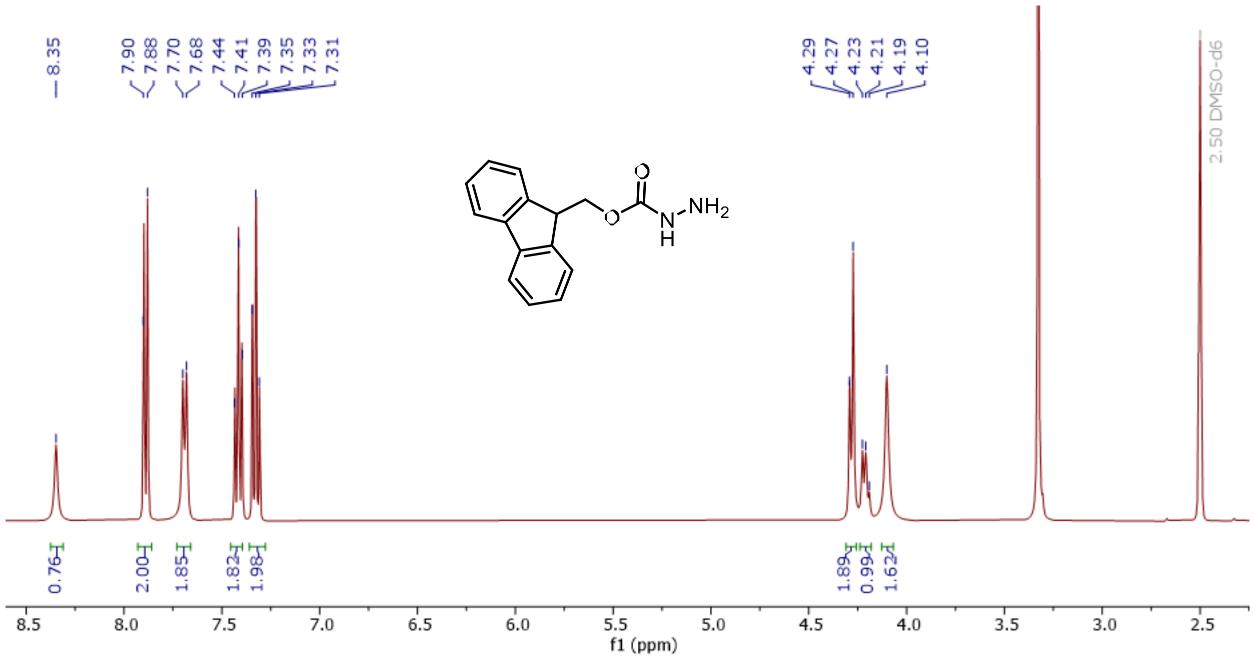

Figure S6: ^{1}H NMR (400 MHz) spectrum of compound 4 (DMSO-d_6, 298 K)

1.4. Compound 5 (^{1}H NMR spectrum)

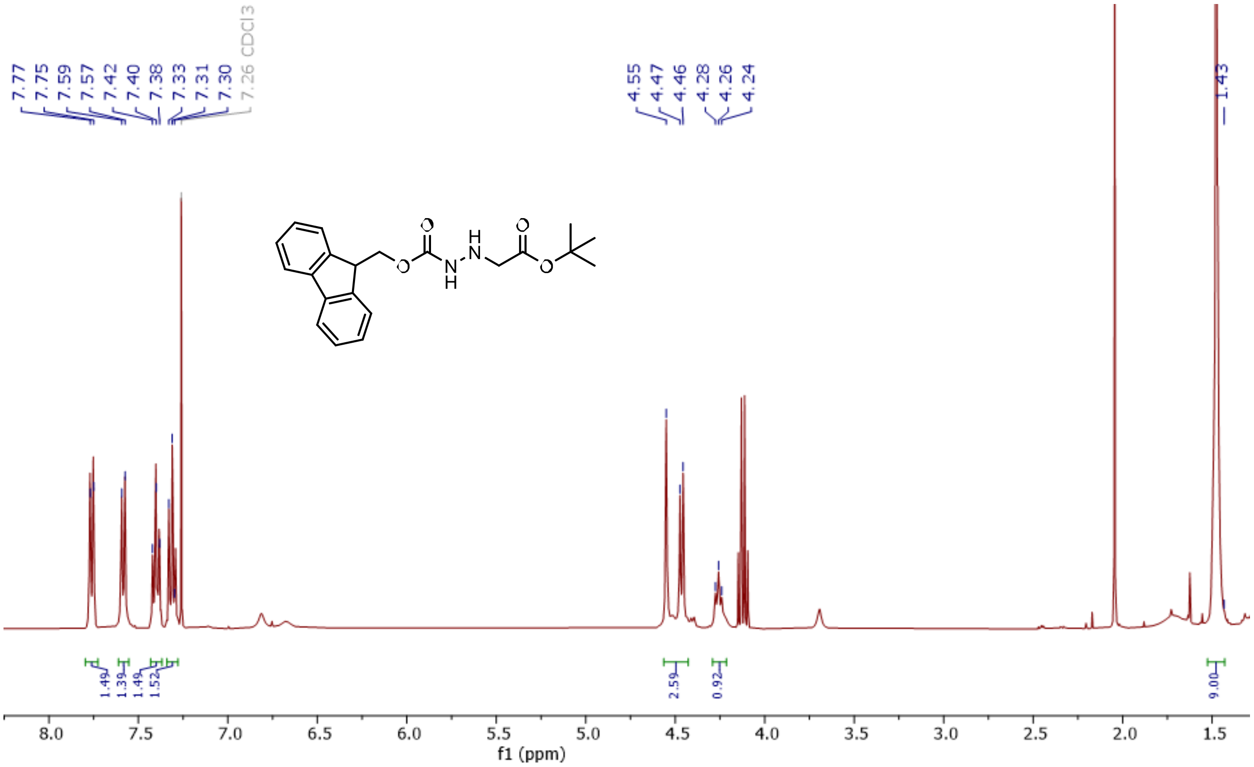

Figure S7: ^{1}H NMR (400 MHz) spectrum of compound 5 ($CDCl_3$, 298 K)

1.5. Compound 6 (^{1}H and ^{13}C-JMOD NMR spectra)

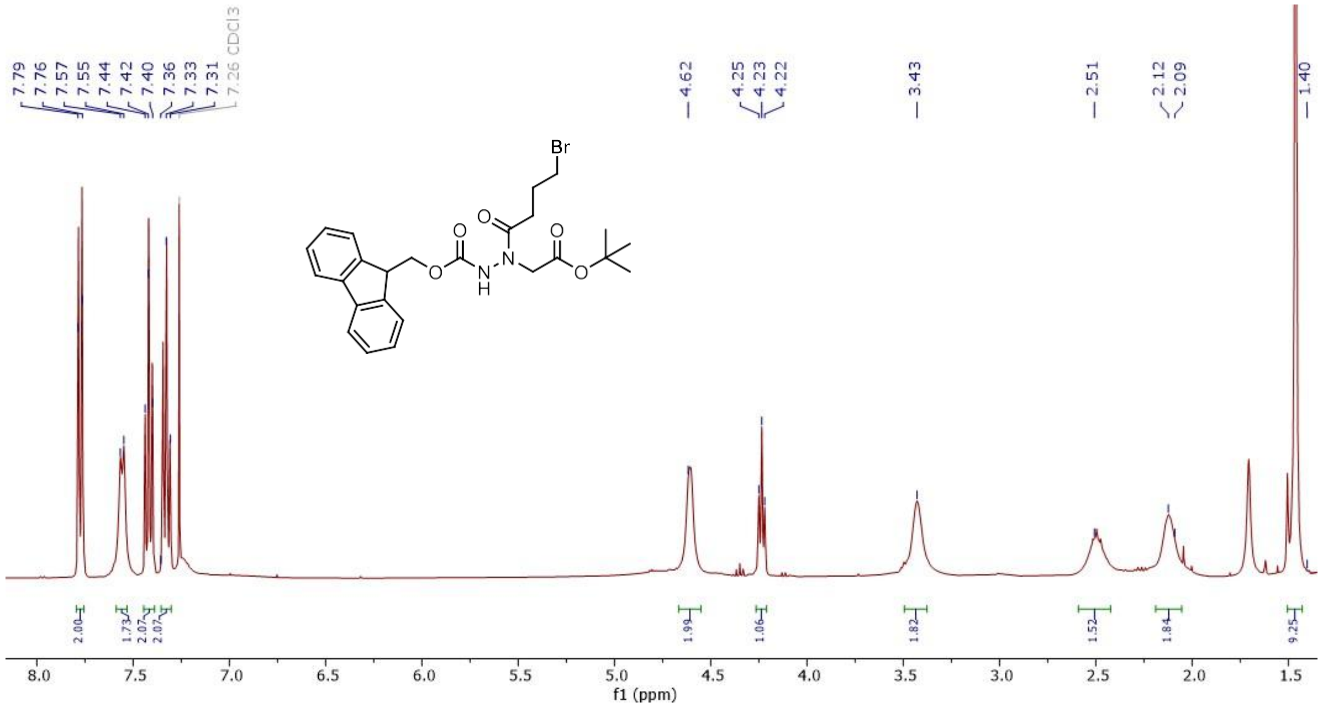

Figure S8: ^{1}H NMR (400 MHz) spectrum of compound 6 ($CDCl_3$, 298 K)

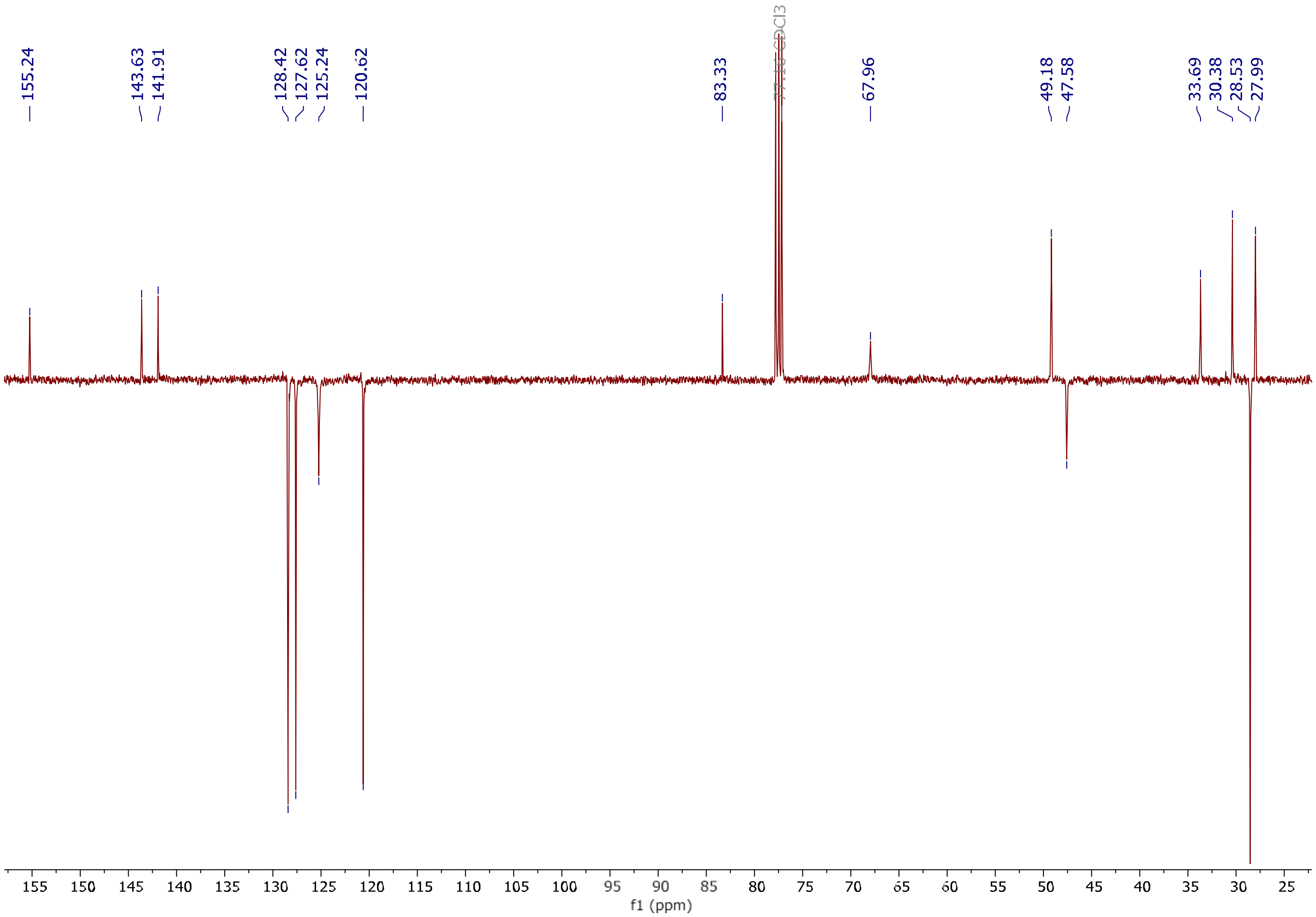

Figure S9: ^{13}C-JMOD NMR (100.6 MHz) spectrum of compound 6 ($CDCl_3$, 298 K)

1.6. Compound 7 (^{1}H and ^{13}C-JMOD NMR spectra)

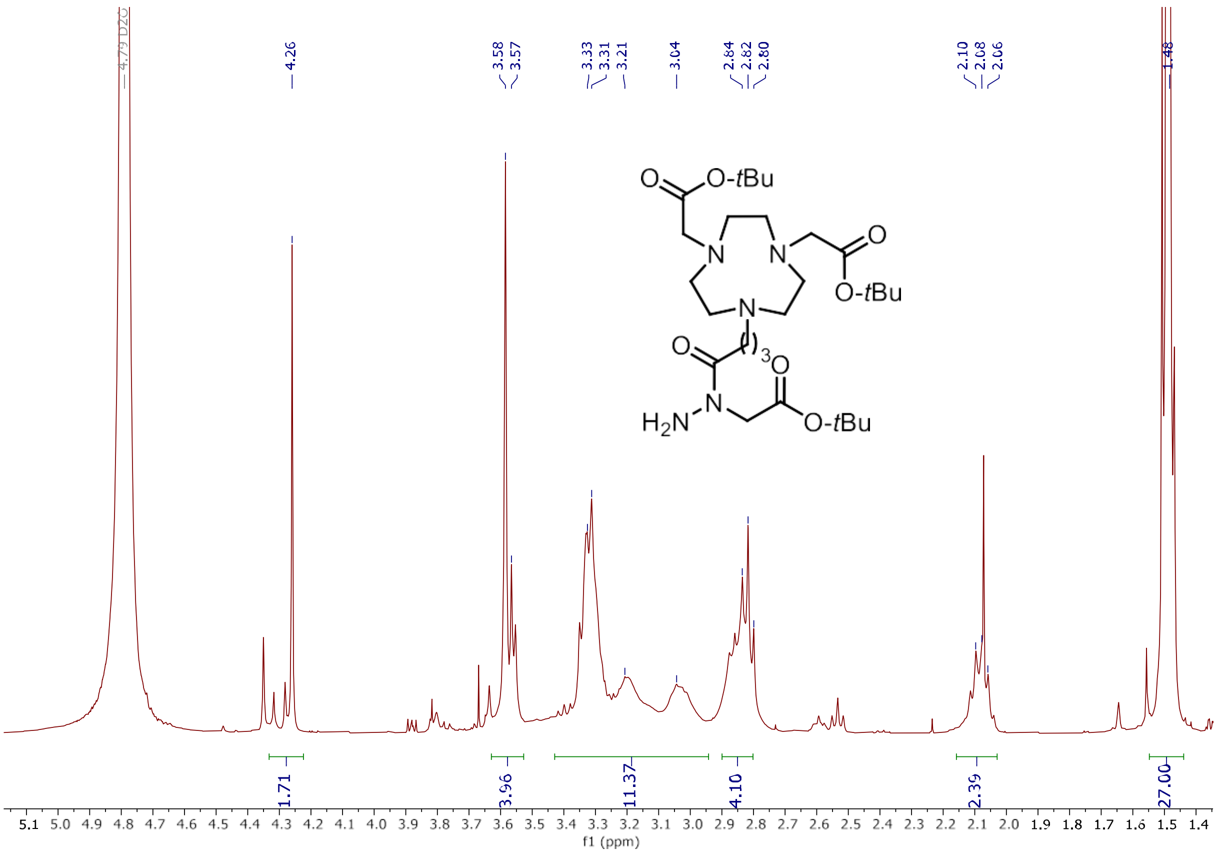

Figure S10: ^{1}H NMR (400 MHz) spectrum of compound 7 (D_2O, 298 K)

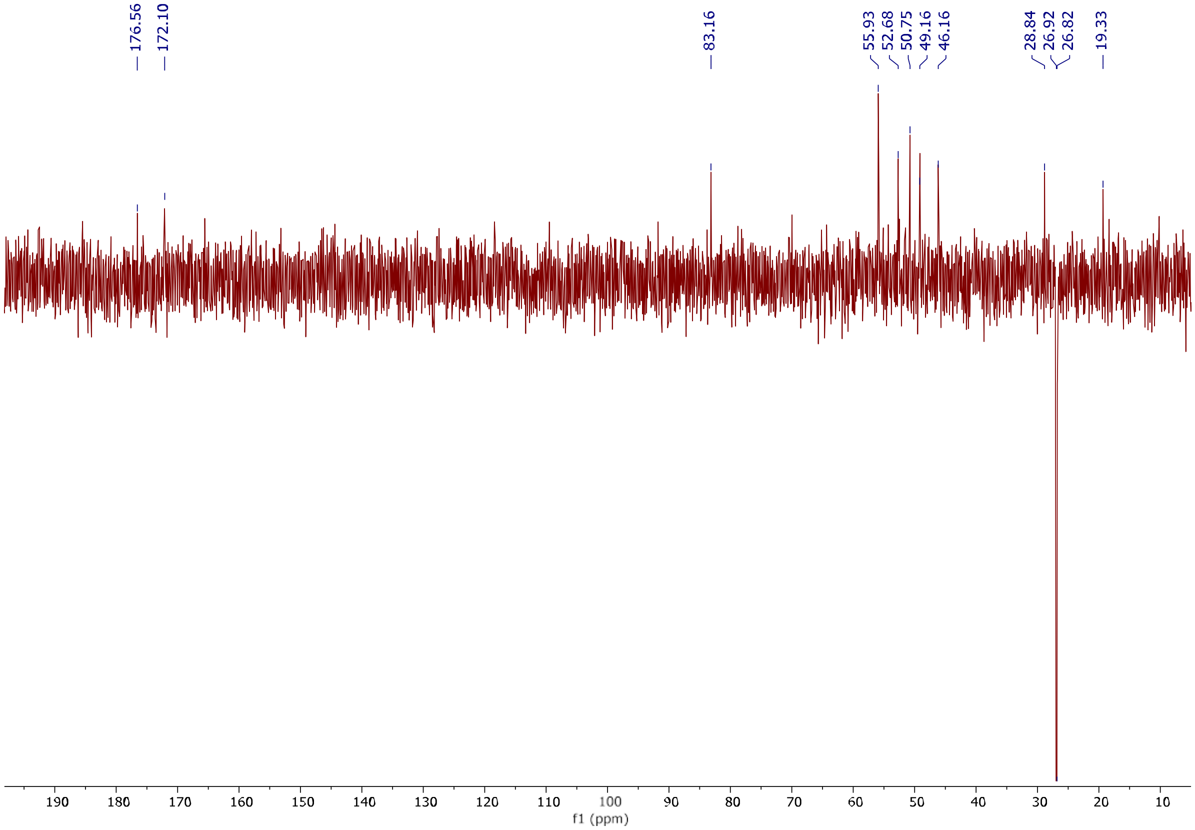

Figure S11: ^{13}C-JMOD NMR (100.6 MHz) spectrum of compound 7 (D_2O, 298 K)

1.7. Compound 8 (^{1}H and ^{13}C-JMOD NMR spectra)

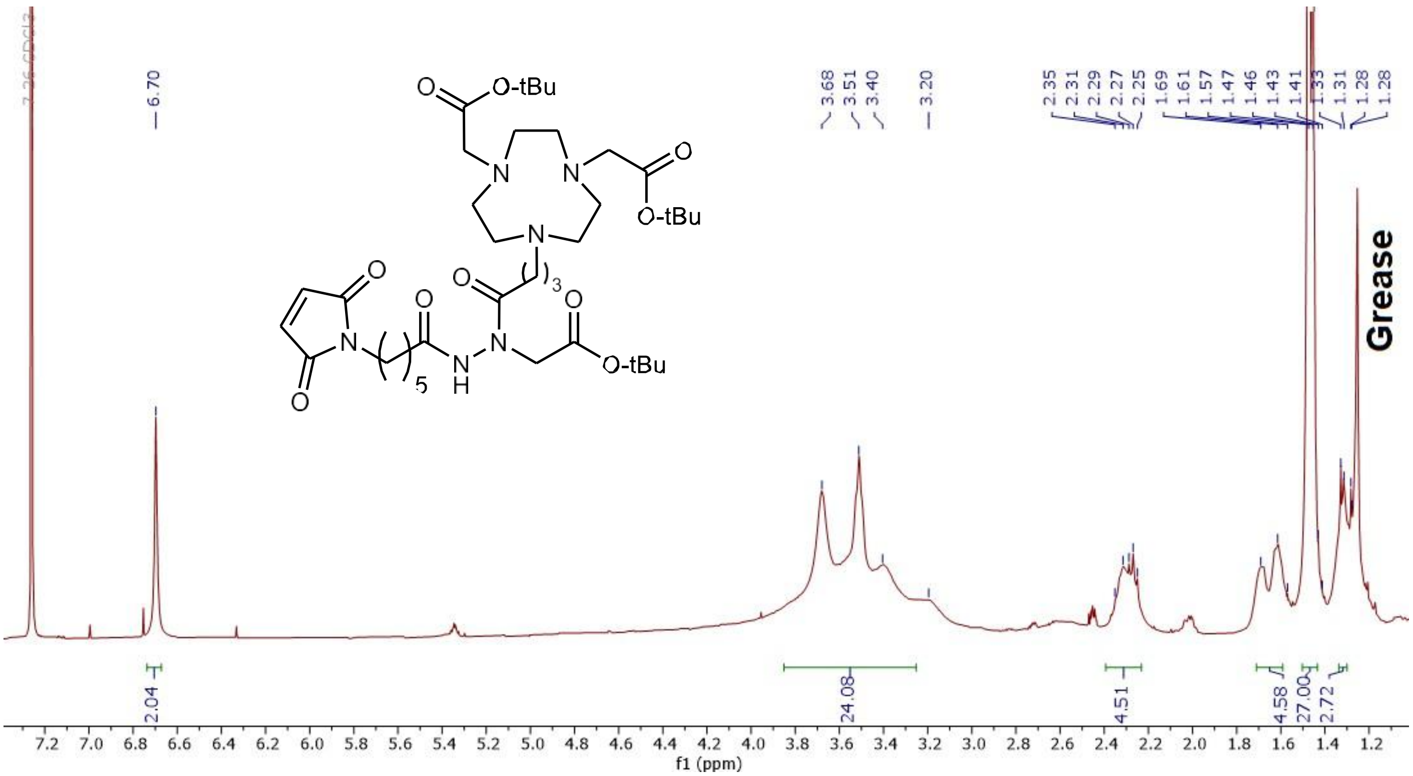

Figure S12: ^{1}H NMR (400 MHz) spectrum of compound 8 ($CDCl_3$, 298 K)

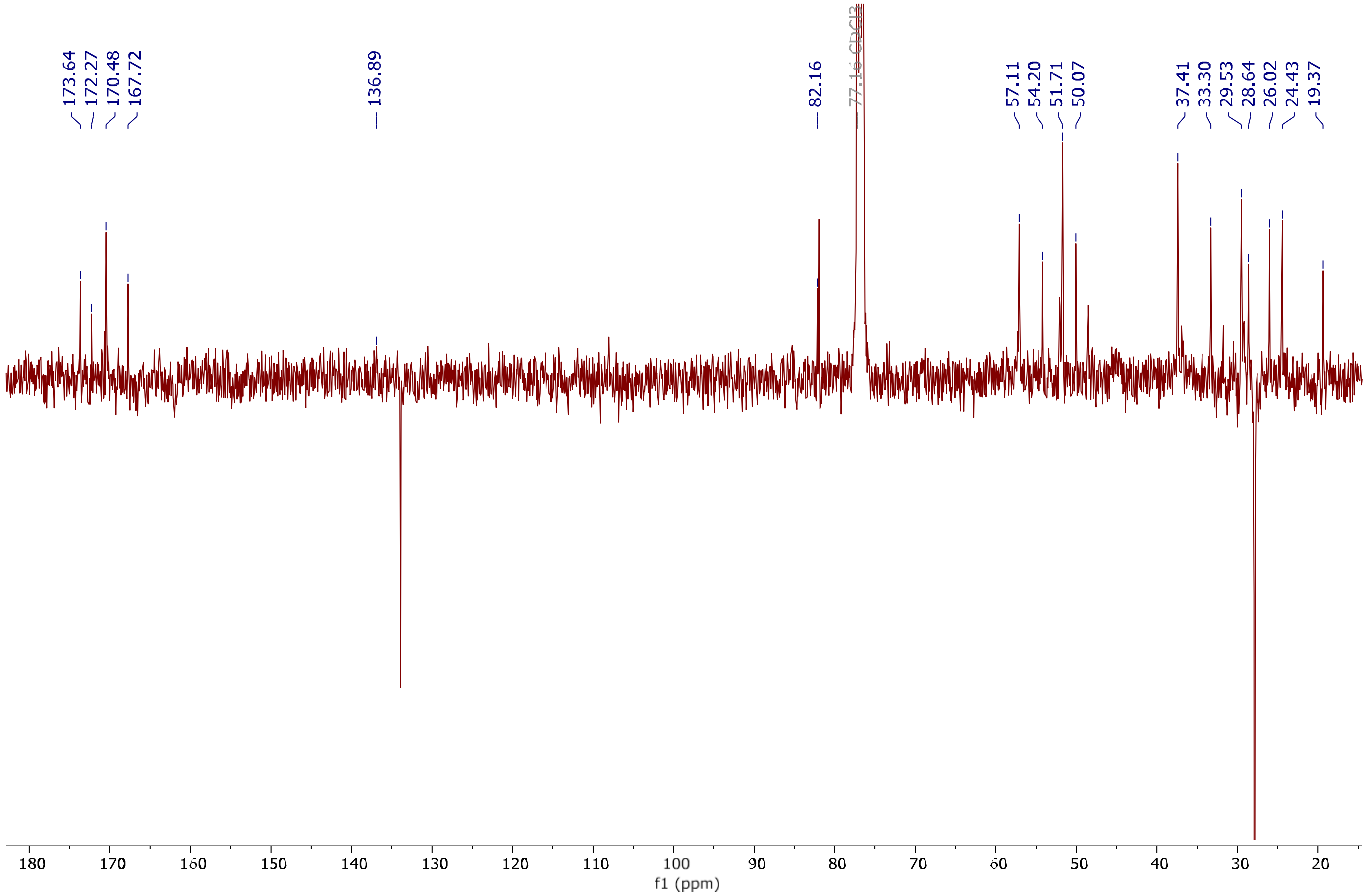

Figure S13: ^{13}C-JMOD NMR (100.6 MHz) spectrum of compound 8 ($CDCl_3$, 298 K)

1.8. NO2A-2C-AHM (^{1}H and ^{13}C-JMOD NMR spectra, and HPLC-MS analysis)

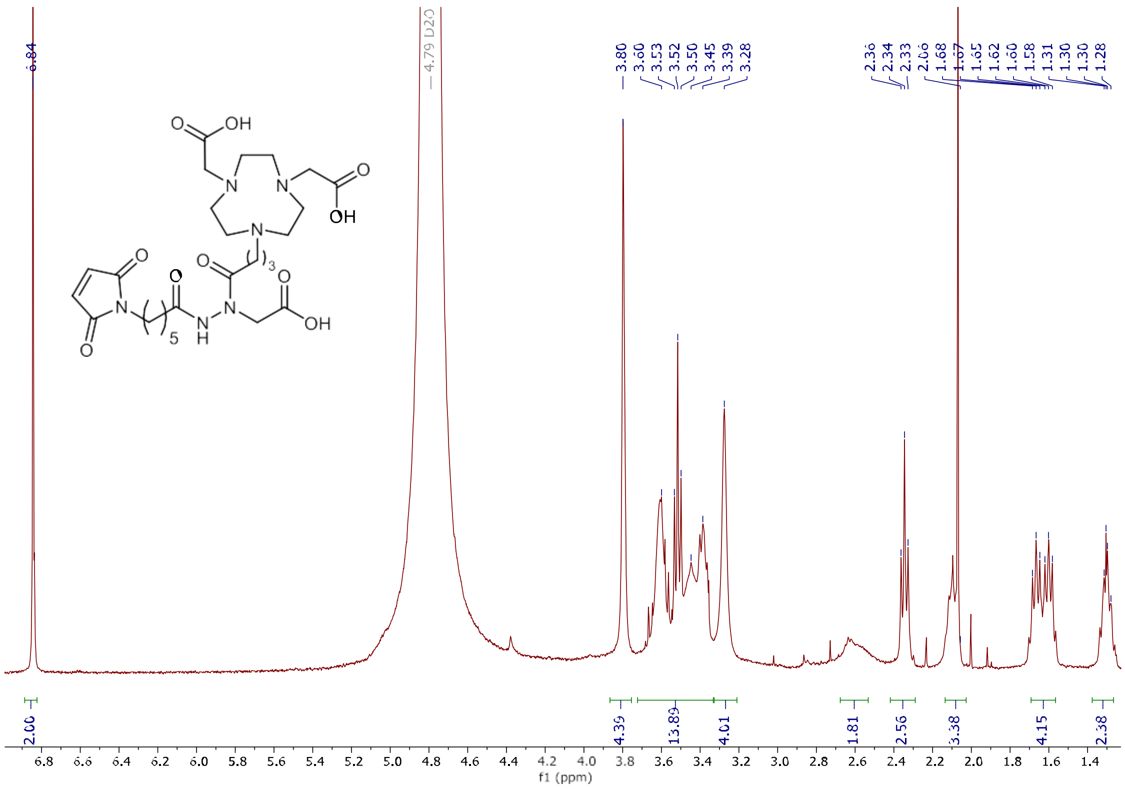

Figure S14: ^{1}H NMR (400 MHz) spectrum of NO2A-2C-AHM (D_2O, 298 K)

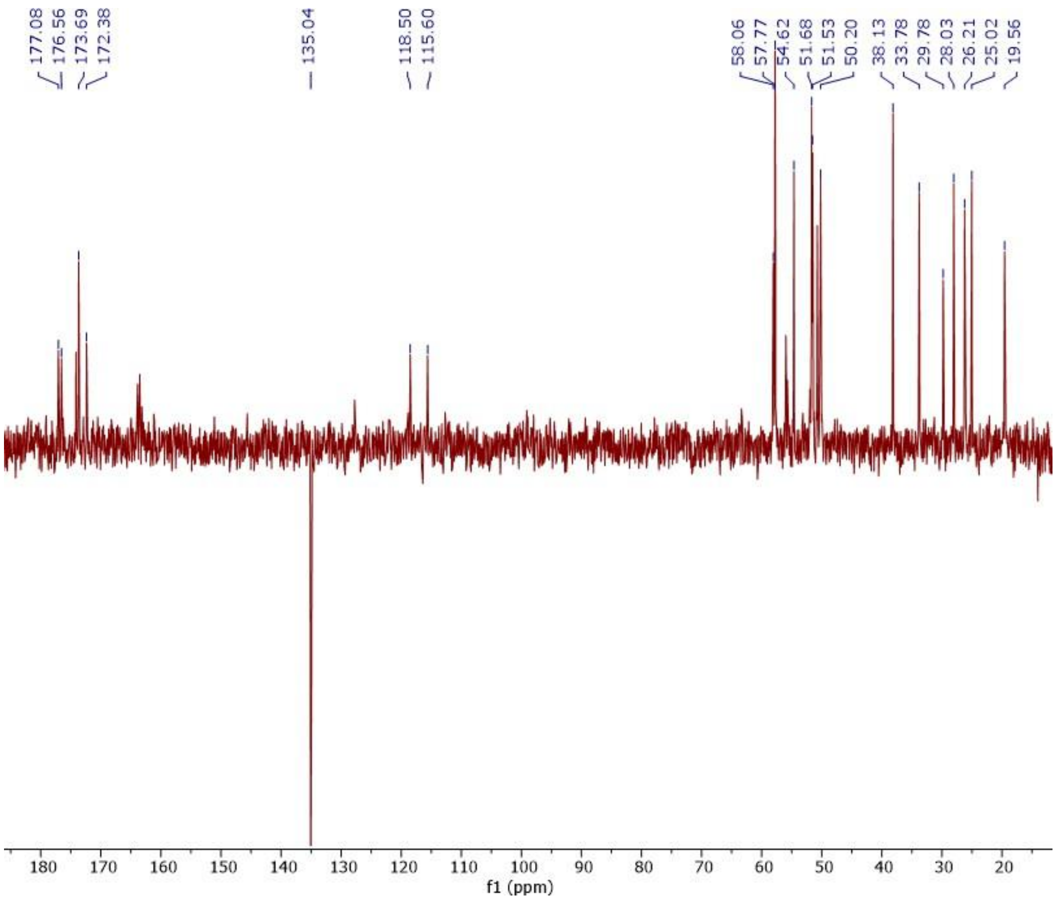

Figure S15: ^{13}C-JMOD NMR (100.6 MHz) spectrum of NO2A-2C-AHM (D_2O, 298 K)

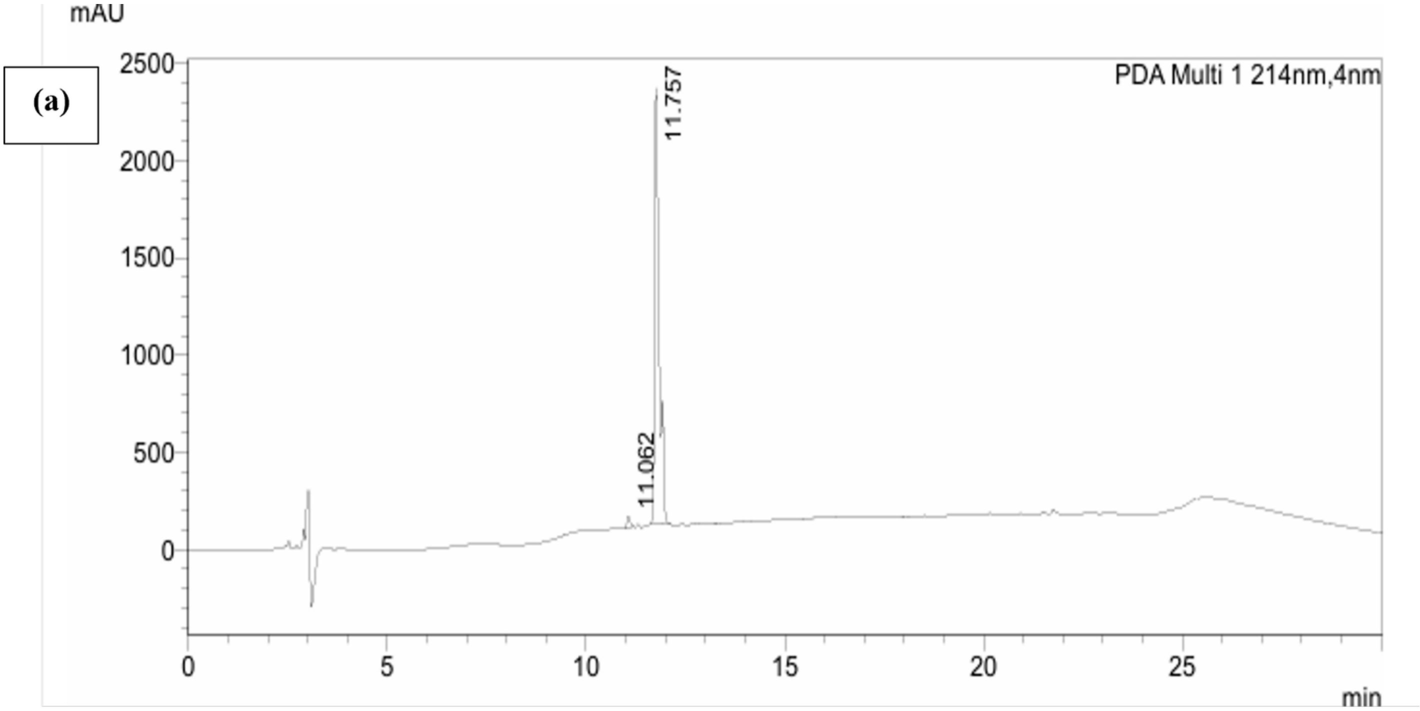

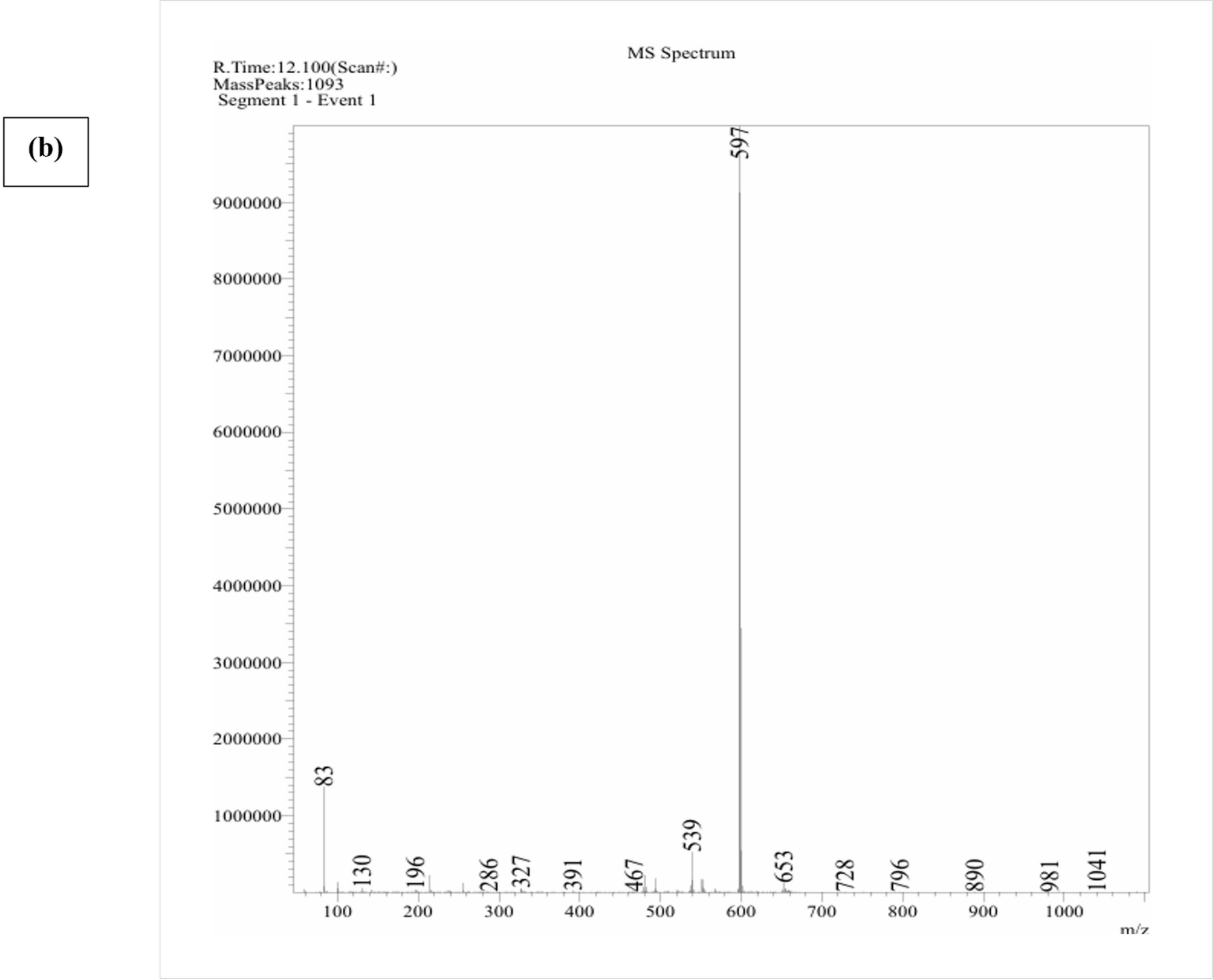

Figure S16: (a) HPLC chromatogram (b) MS spectrum of NO2A-2C-AHM

1.9. NODAGA-HM (^{1}H and ^{13}C-JMOD NMR spectra, and HPLC-MS analysis)

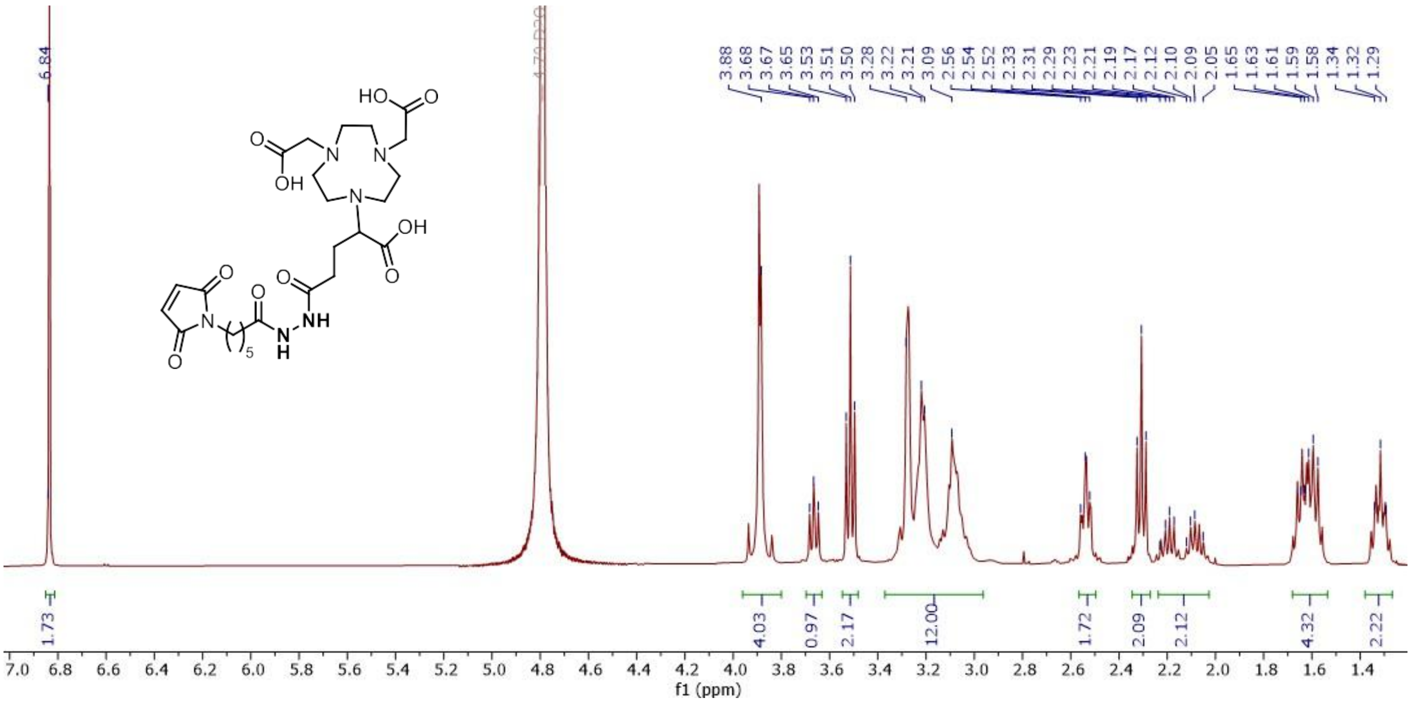

Figure S17: ^{1}H NMR (400 MHz) spectrum of NODAGA-HM (D_2O, 298 K)

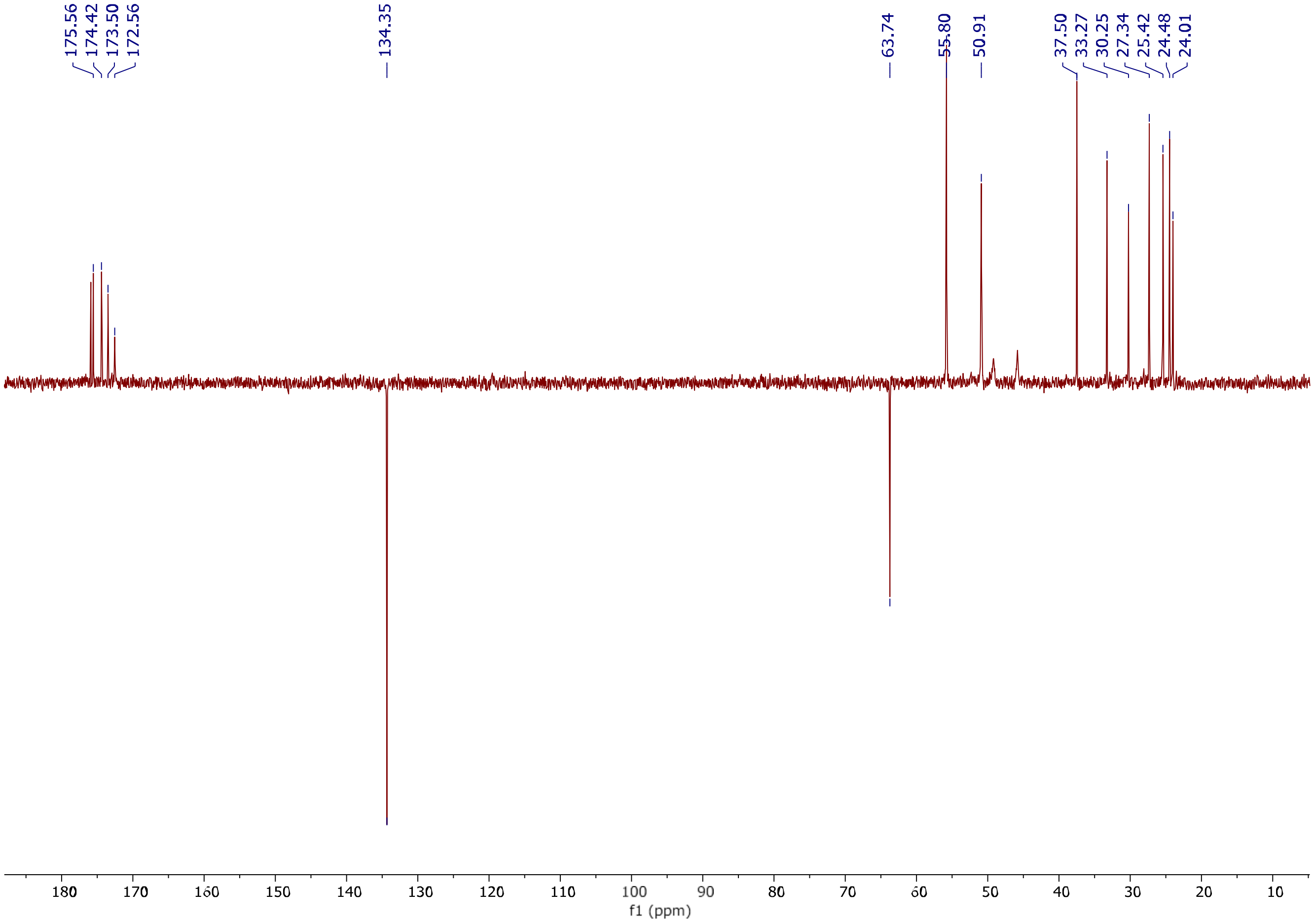

Figure S18: ^{13}C-JMOD NMR (100.6 MHz) spectrum of NODAGA-HM (D_2O, 298 K)

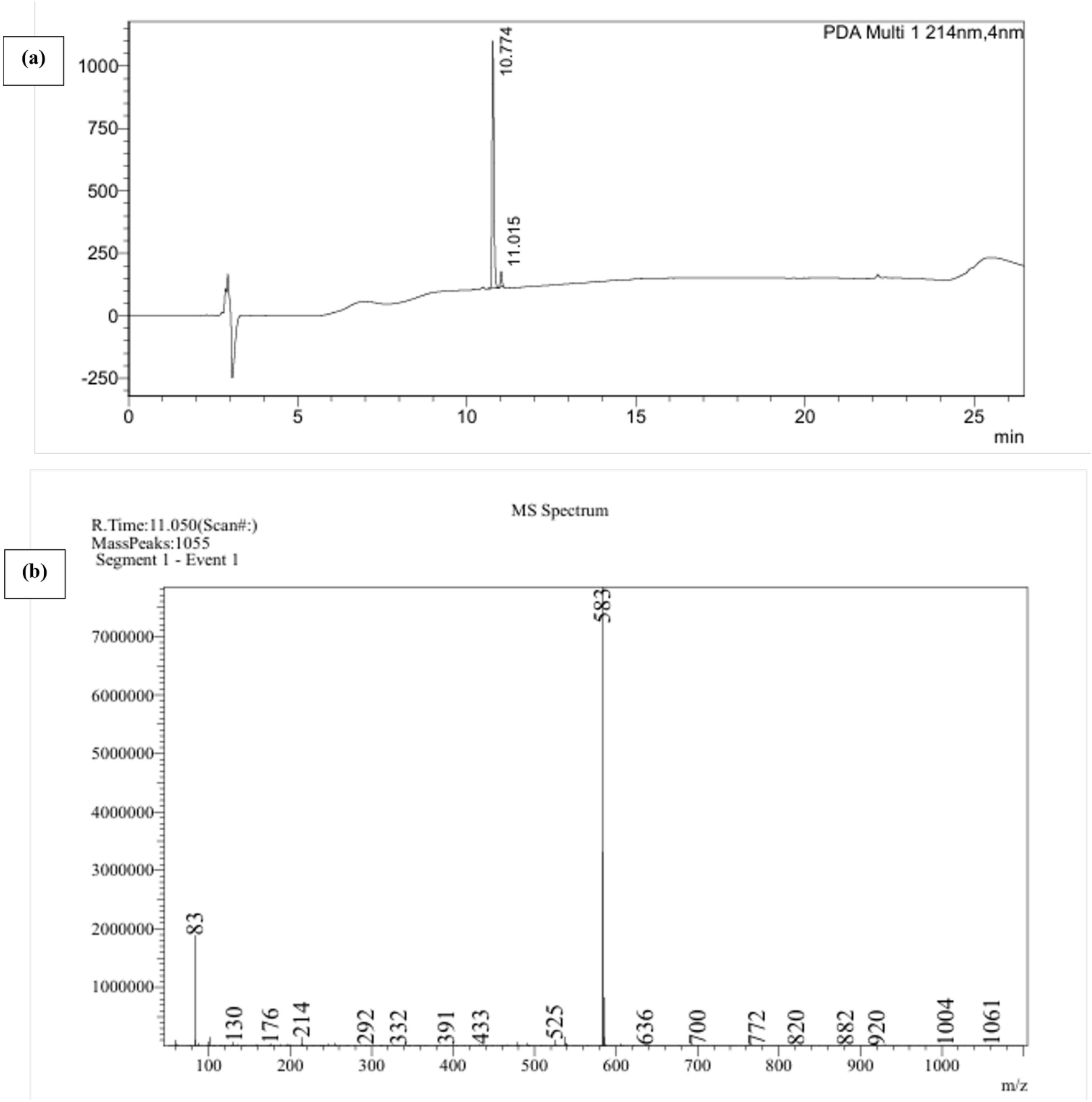

Figure S19: (a) HPLC chromatogram (b) MS spectrum of NODAGA-HM

1.10. AlF-NO2A-2C-AHM (^{1}H and ^{19}F NMR spectra, and HPLC-MS analysis)

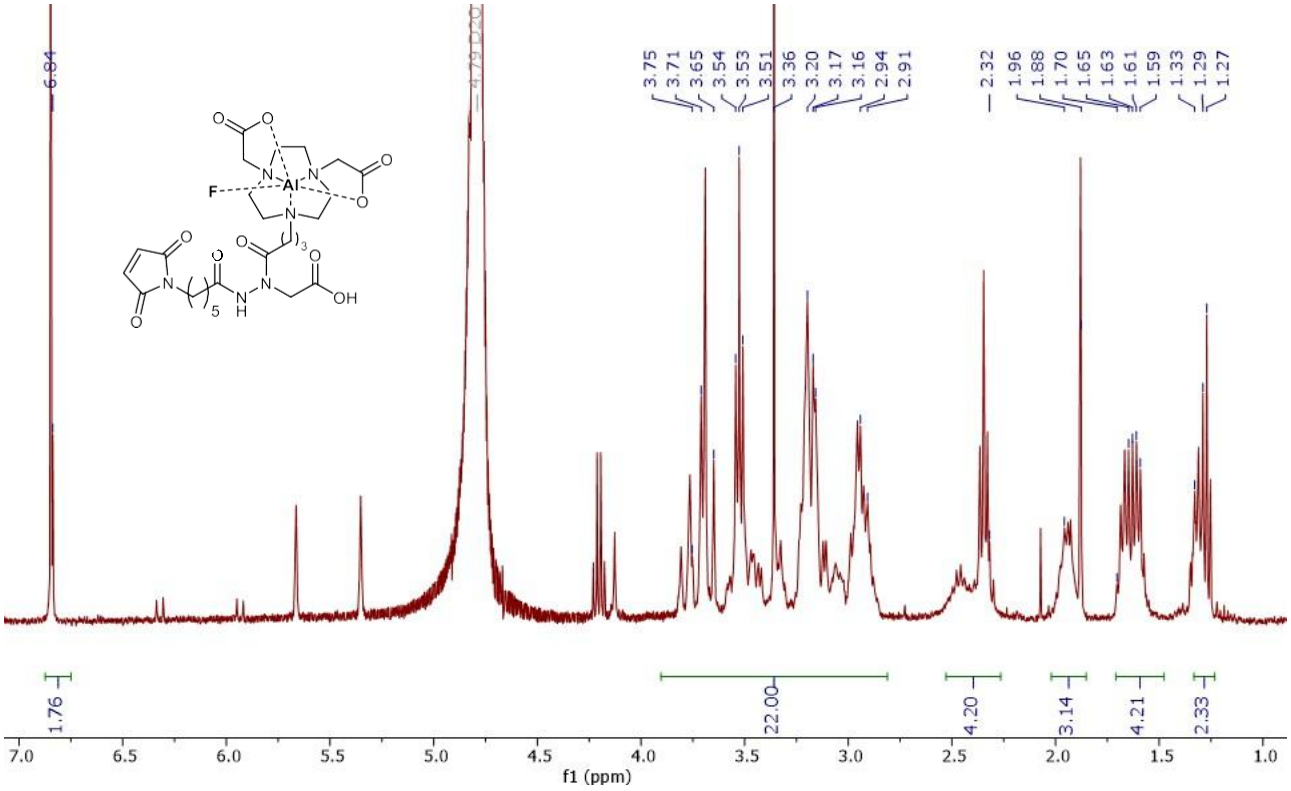

Figure S20: ^{1}H NMR (400 MHz) spectrum of AlF-NO2A-2C-AHM (D_2O, 330 K)

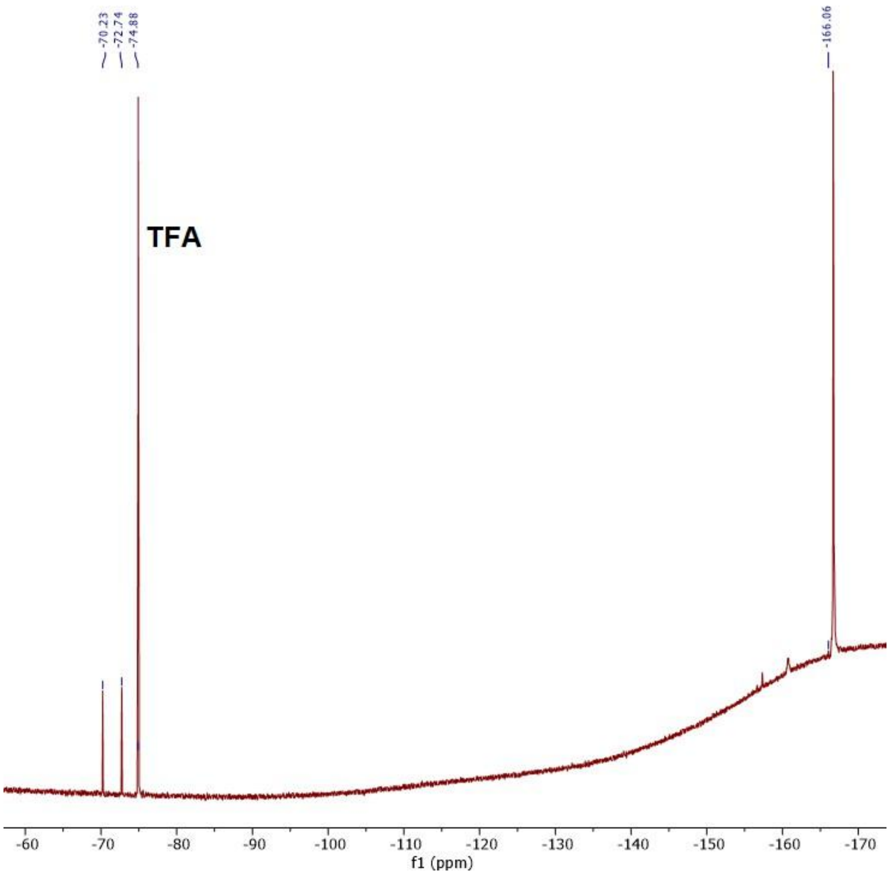

Figure S21: ^{19}F NMR (376 MHz) spectrum of AlF-NO2A-2C-AHM (D_2O, 330 K)

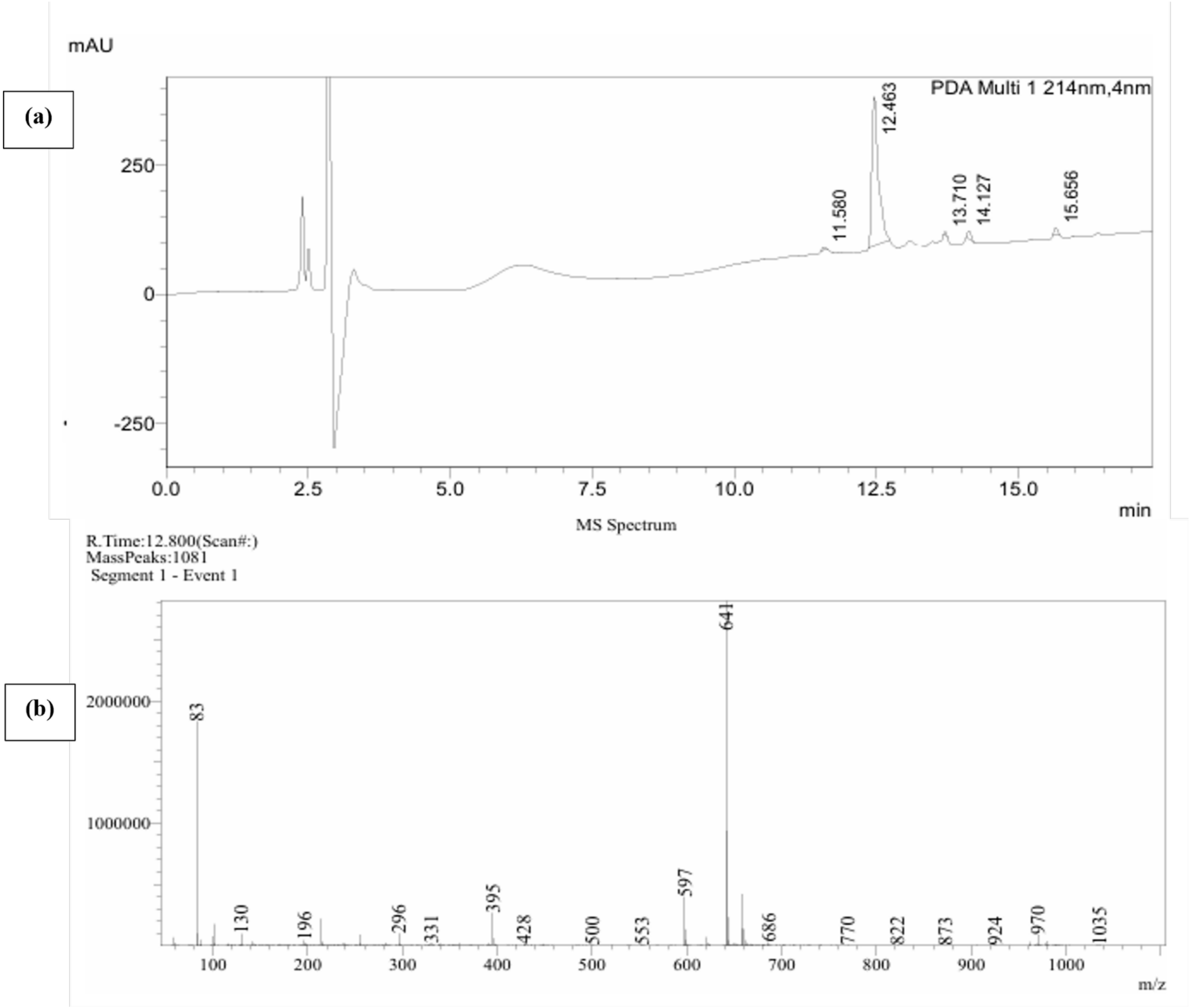

Figure S22: (a) HPLC chromatogram (b) MS spectrum of AlF-NO2A-2C-AHM

1.11. AlF-NODAGA-HM (^{1}H and ^{19}F NMR spectra, and HPLC-MS analysis)

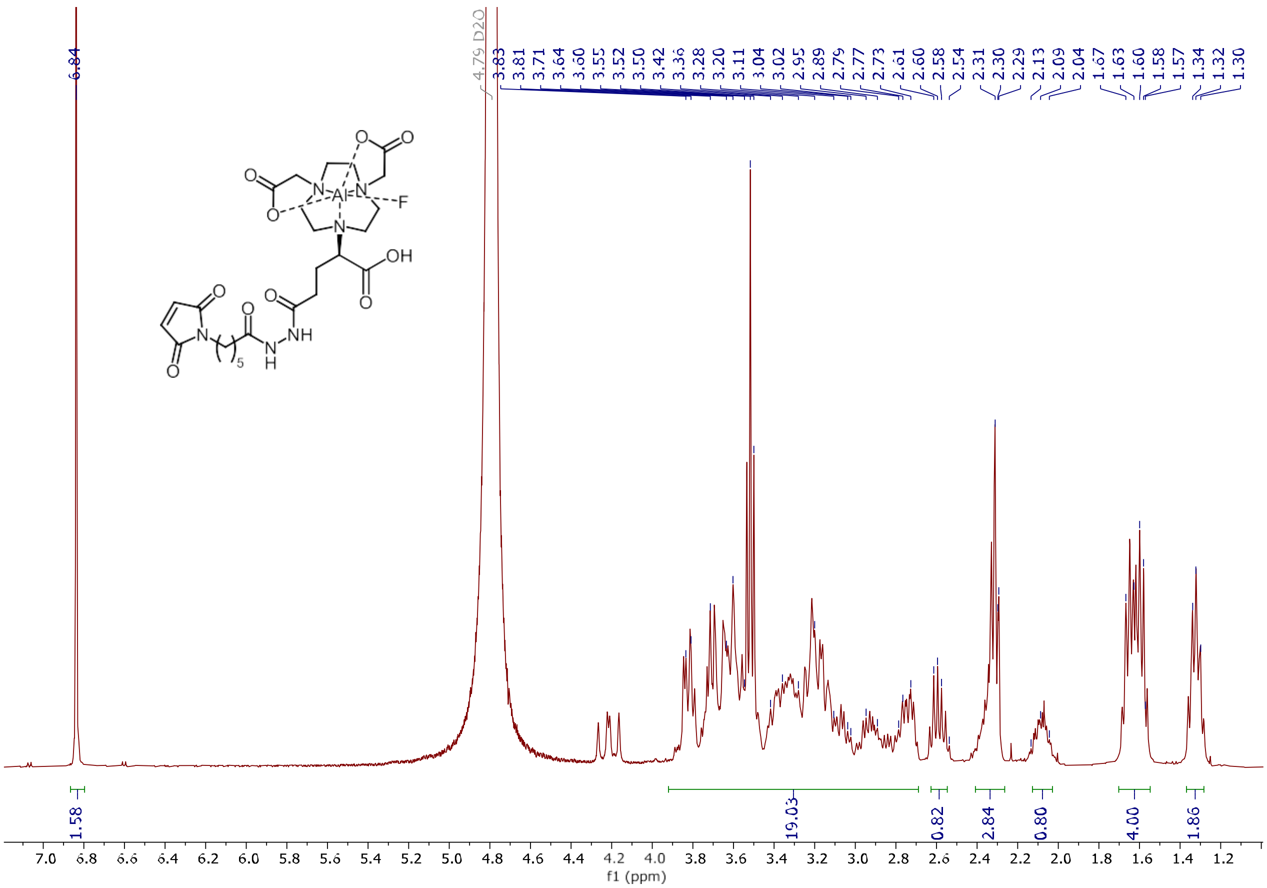

Figure S23: ^{1}H NMR (400 MHz) spectrum of AlF-NODAGA-HM (D_2O, 330 K)

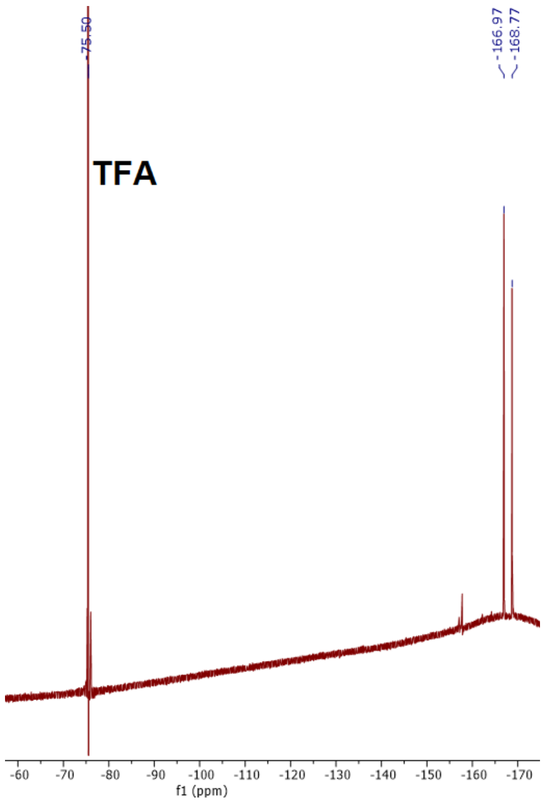

Figure S24: ^{19}F NMR (376 MHz) spectrum of AlF-NODAGA-HM (D_2O, 330 K)

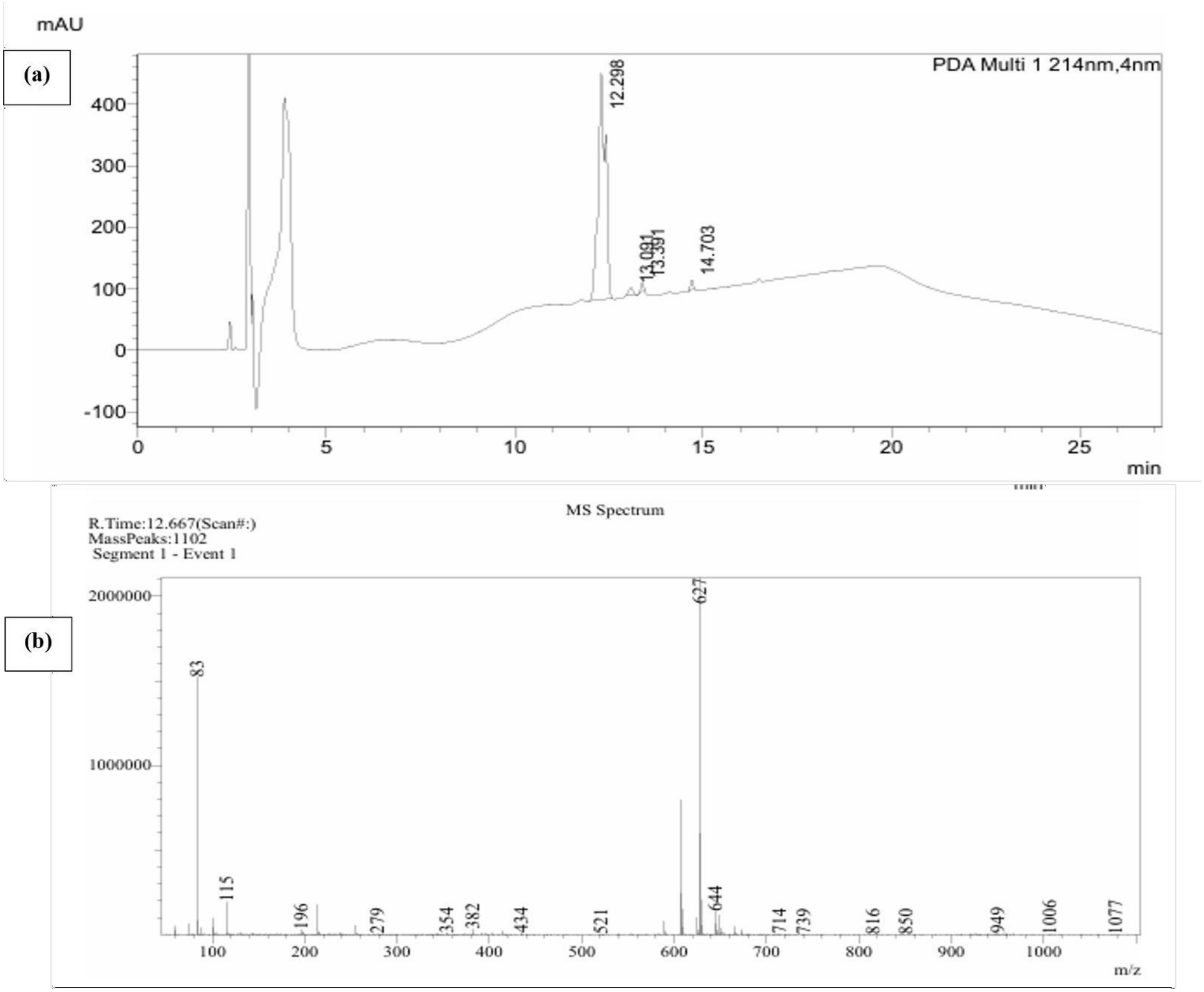

Figure S25: (a) HPLC chromatogram (b) MS spectrum of AlF-NODAGA-HM

1.12. Lu-NODAGA-HM (^{1}H NMR spectrum, and HPLC-MS analysis)

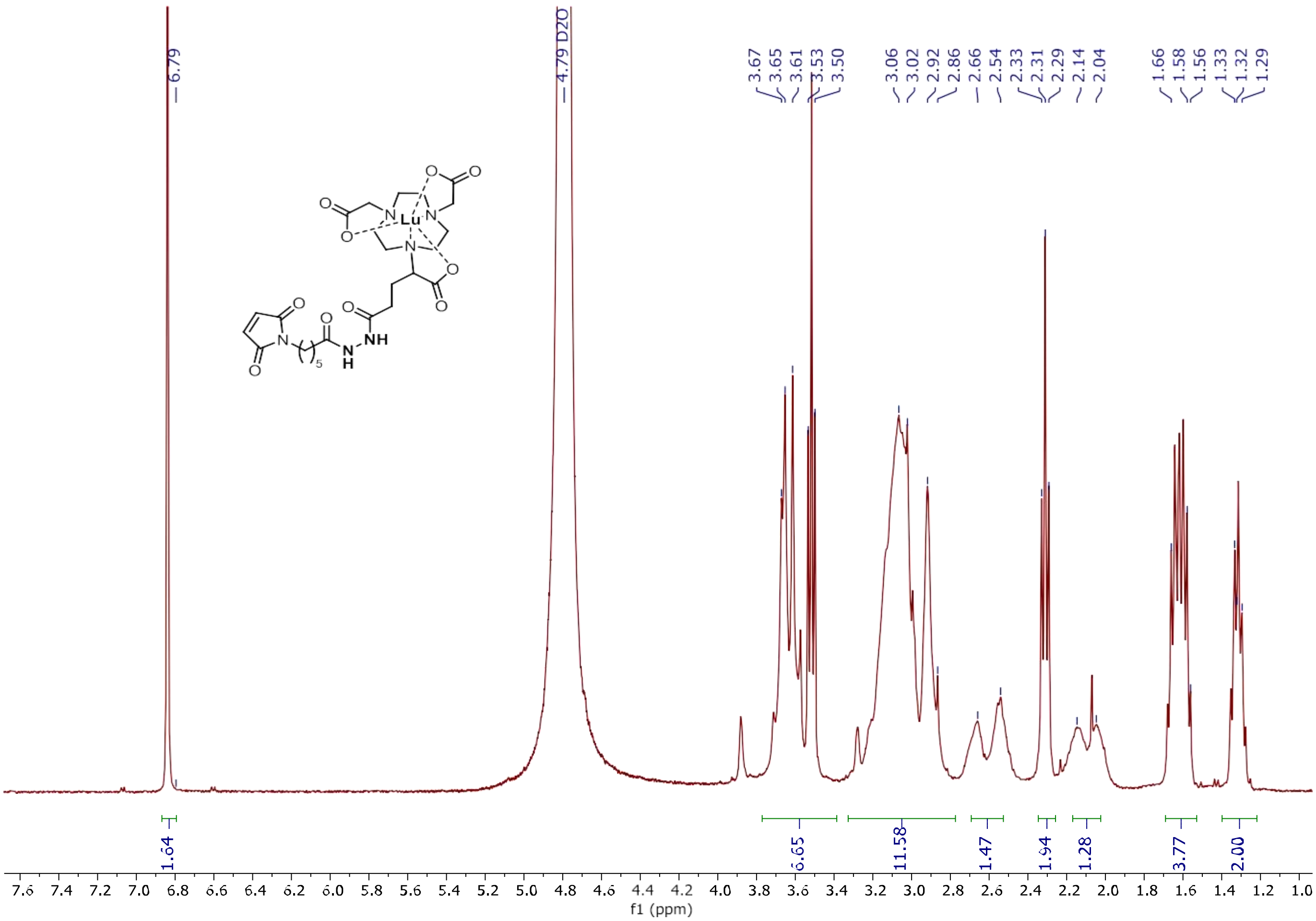

Figure S26: ^{1}H NMR (400 MHz) spectrum of Lu-NODAGA-HM (D_2O, 330 K)

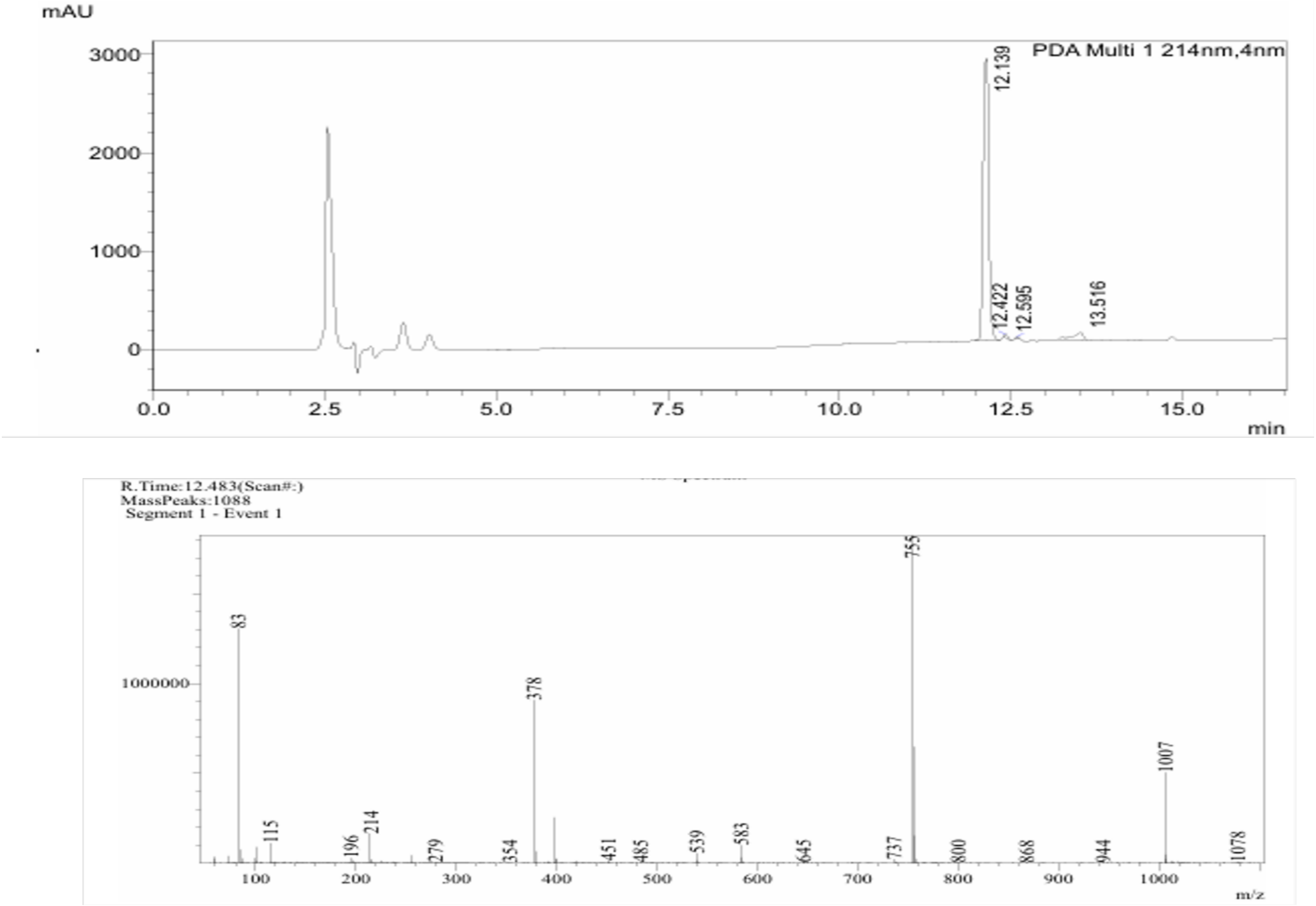

Figure S27: (a) HPLC chromatogram (b) MS spectrum of Lu-NODAGA-HM

2. Radiolabeling

2.1. [^{18}F]AlF-NODAGA-HM

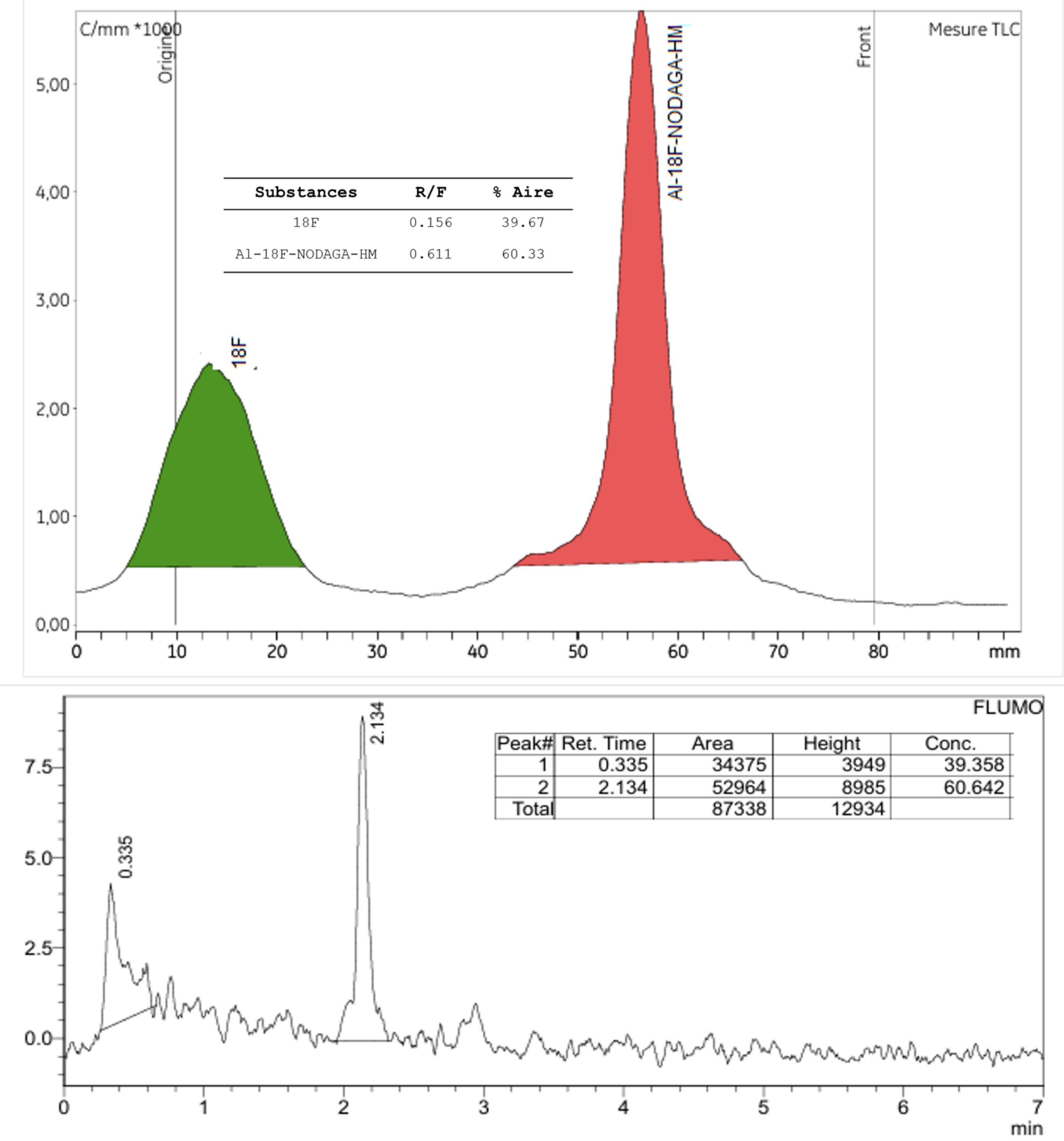

Figure S28: Radio-TLC and radio-HPLC of [^{18}F]AlF-NODAGA-HM

2.2. [^{18}F]AlF-NO2A-2C-AHM

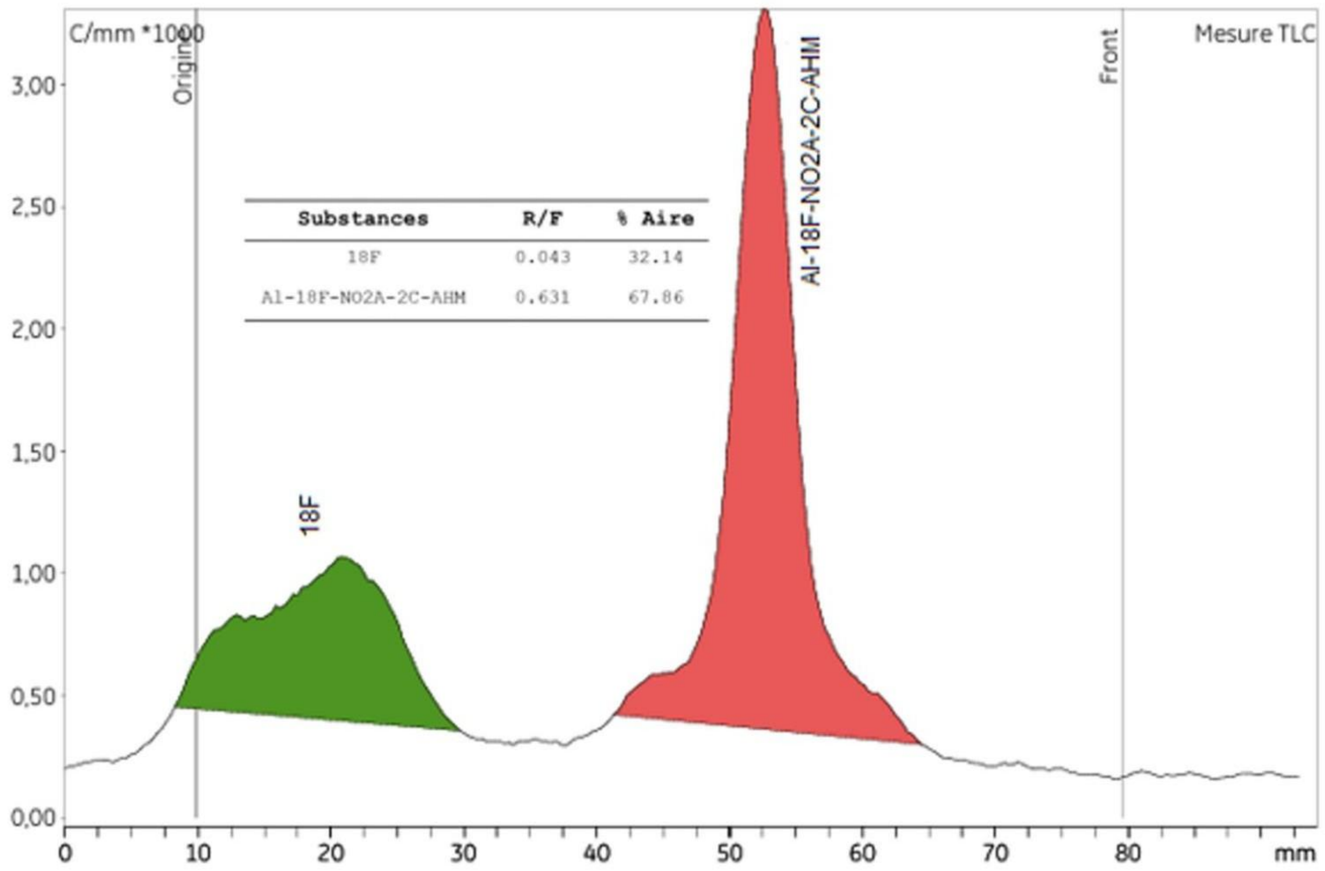

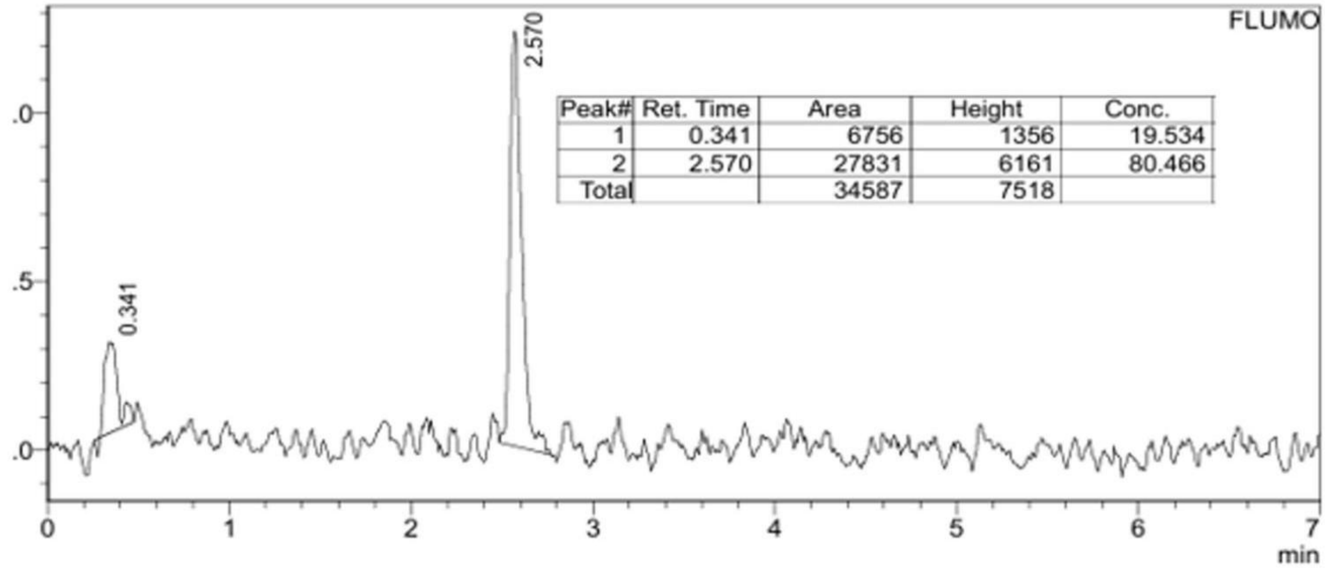

Figure S29: Radio-TLC and radio-HPLC of [^{18}F]AlF-NO2A-2C-AHM

2.3. [^{177}Lu]Lu-NODAGA-HM

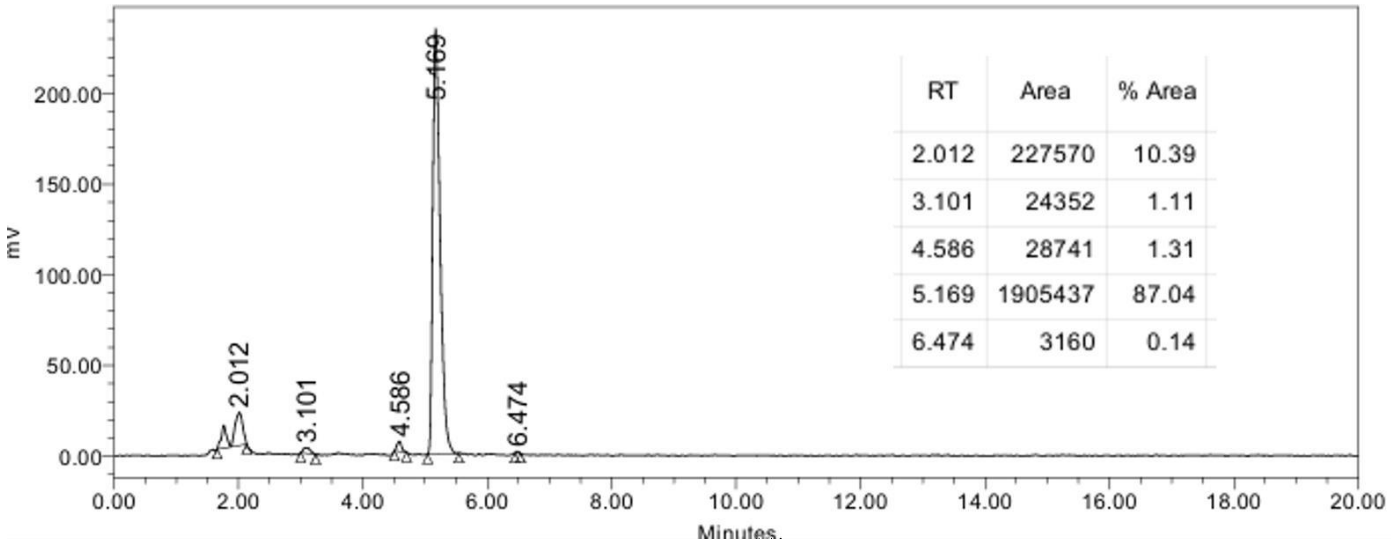

Figure S30: Radio-HPLC of [^{177}Lu]Lu-NODAGA-HM

3. Molecular Modeling Data

3.1. AlF-NO2A-2C-AHM and AlF-NODAGA-HM

Table S1: Bond lengths and bond angles for complex Al-F-NODAGA-HM and Al-F-NO2A-2C-AHM

**Al-F-NO2A-2C-AHM**				**Al-NODAGA-HM**			
**Bond length (Å)**		**Bond Angle (deg)**		**Bond length (Å)**		**Bond Angle (deg)**	
Al-N1	2.11582	N1-Al-O1	81.79618	Al-N1	2.19849	N1-Al-O1	81.04049
Al-N3	2.07701	N1-Al-N2	82.11775	Al-N2	2.15242	N1-Al-N2	79.61694
Al-N2	2.08814	N1-Al-N3	83.43094	Al-N3	2.19612	N1-Al-N3	82.36580
Al-F	1.70347	O1-Al-O2	97.83937	Al-F	1.70416	O1-Al-O2	98.28839
Al-O1	1.83902	O1-Al-F	107.79315	Al-O1	1.80088	O1-Al-F	96.34479
Al-O2	1.89355	O1-Al-N2	93.61492	Al-O2	1.84123	O1-Al-N2	80.02897
		O1-Al-N3	166.92922			O1-Al-N3	161.09540
		O2-Al-N2	81.32064			O2-Al-N2	160.64218
		O2-Al-N3	91.61492			O2-Al-N3	95.59088
		F-Al-N2	88.23642			F-Al-N2	93.32270
		F-Al-N3	90.06801			F-Al-N3	92.36772
		N2-Al-N3	84.52361			N2-Al-N3	82.61659
		N1-Al-F	169.29502			N1-Al-F	172.40168
		O2-Al-F	94.17282			O2-Al-F	94.34479

3.2. Lu-NODAGA-HM

Table S2: Bond lengths and bond angles for complex Lu-NODAGA-HM

Hexa-Lu-NODAGA-HM			
**Bond length (Å)**		**Bond Angle (deg)**	
Lu-N1	2.52467	N1-Lu-O1	70.91897
Lu-N2	2.52711	N1-Lu-N2	70.81049
Lu-N3	2.51219	N1-Lu-N3	72.97034
Lu-O1	2.12646	N1-Lu-O3	107.02957
Lu-O2	2.13438	N1-Lu-O2	141.40721
Lu-O3	2.10602	O1-Lu-O2	120.19126
		O1-Lu-O3	108.89035
		O1-Lu-N2	103.59307
		O1-Lu-N3	142.09789
		O2-Lu-O3	103.40286
		O2-Lu-N2	72.60415
		O2-Lu-N3	95.37899
		O3-Lu-N2	143.70876
		O3-Lu-N3	71.35386
		N2-Lu-N3	73.14629
